# Supplementary material for: Reliability and agreement of a novel portable laser height metre
Source: PLoS One. 2020 Apr 8;15(4):e0231449. doi: 10.1371/journal.pone.0231449 (PMC7141692; doi:10.1371/journal.pone.0231449)
Supplement: S1 Appendix — (PDF) [file pone.0231449.s001.pdf]

Appendix 1

Codebook for variables

Histograms and QQ-plots used to check assumptions for paired t-test for:

- Intrarater reliability
- Interrater reliability
- Method comparison

Histograms and QQ-plots used to check distribution for:

- Age
- Log_age

Codebook for variables

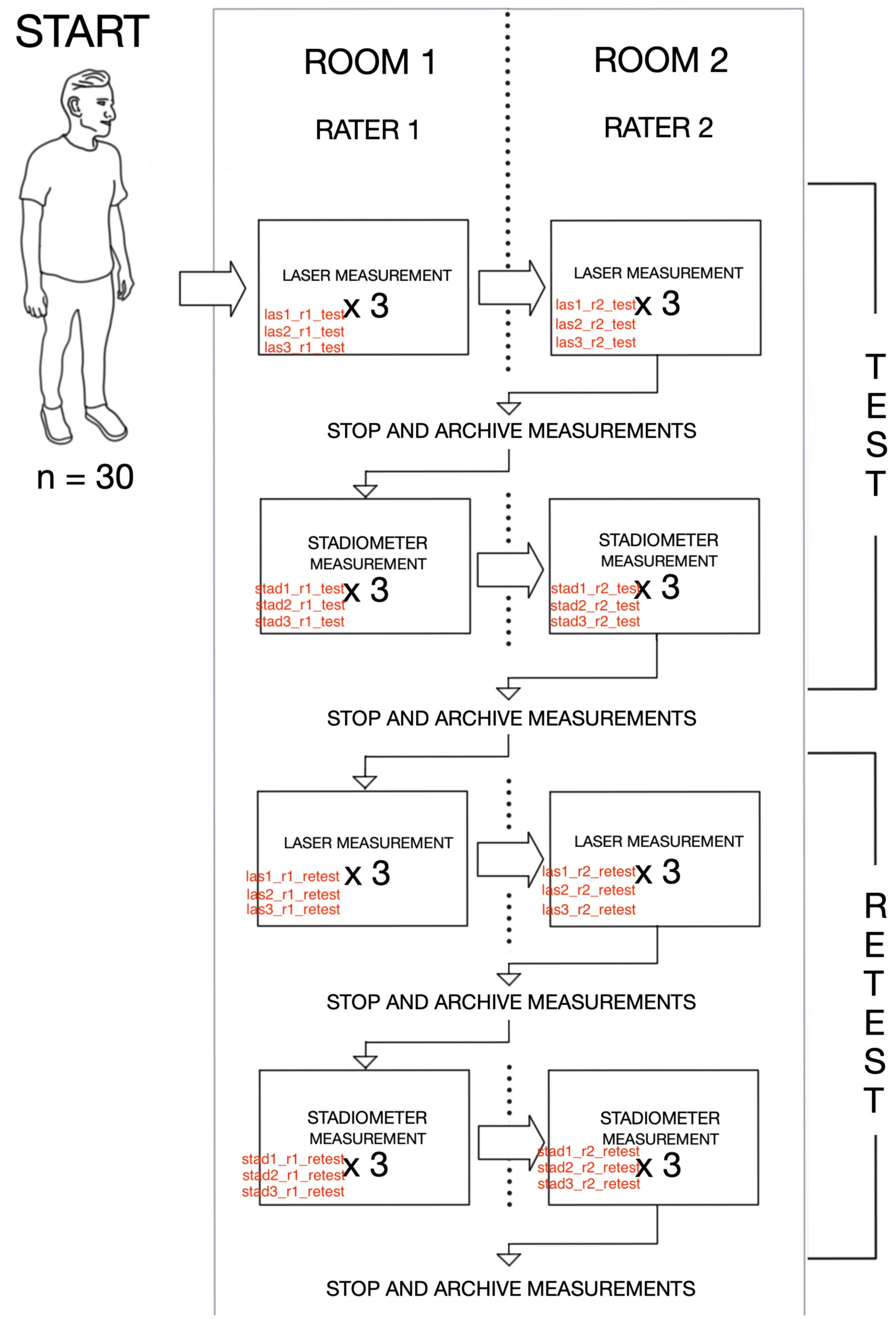
START
n = 30
ROOM 1
RATER 1
ROOM 2
RATER 2
LASER MEASUREMENT
x 3
las1_r1_test
las2_r1_test
las3_r1_test
LASER MEASUREMENT
x 3
las1_r2_test
las2_r2_test
las3_r2_test
STOP AND ARCHIVE MEASUREMENTS
STADIOMETER MEASUREMENT
x 3
stad1_r1_test
stad2_r1_test
stad3_r1_test
STADIOMETER MEASUREMENT
x 3
stad1_r2_test
stad2_r2_test
stad3_r2_test
STOP AND ARCHIVE MEASUREMENTS
LASER MEASUREMENT
x 3
las1_r1_retest
las2_r1_retest
las3_r1_retest
LASER MEASUREMENT
x 3
las1_r2_retest
las2_r2_retest
las3_r2_retest
STOP AND ARCHIVE MEASUREMENTS
STADIOMETER MEASUREMENT
x 3
stad1_r1_retest
stad2_r1_retest
stad3_r1_retest
STADIOMETER MEASUREMENT
x 3
stad1_r2_retest
stad2_r2_retest
stad3_r2_retest
STOP AND ARCHIVE MEASUREMENTS
TEST
RETEST

Histograms and QQ-plots used to check assumptions for paired t-test for:

- Intrarater reliability
- Interrater reliability
- Method comparison

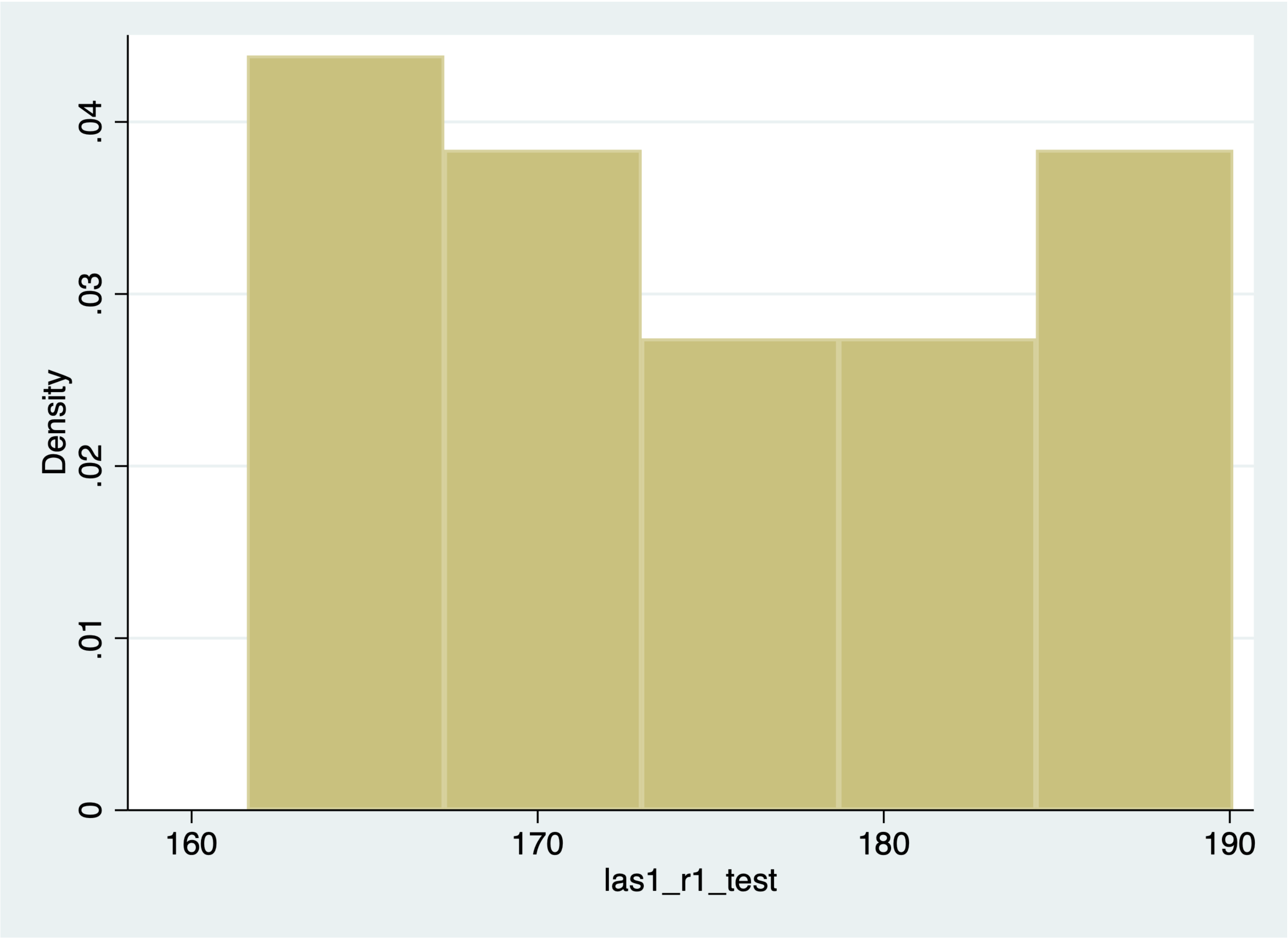

Density
0
.01
.02
.03
.04
160
170
180
190
las1_r1_test

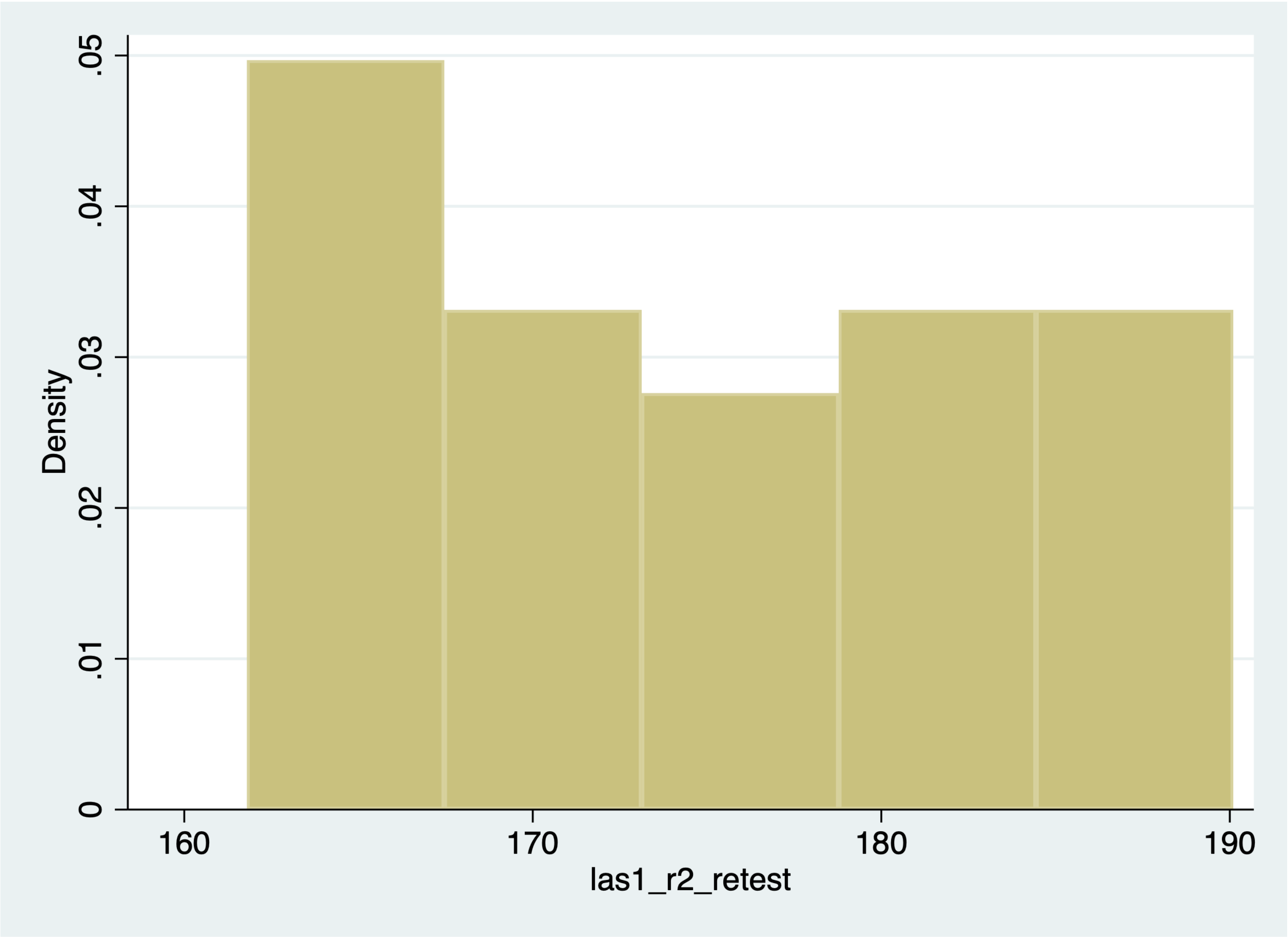

Density
0
.01
.02
.03
.04
.05
160
170
180
190
las1_r2_retest

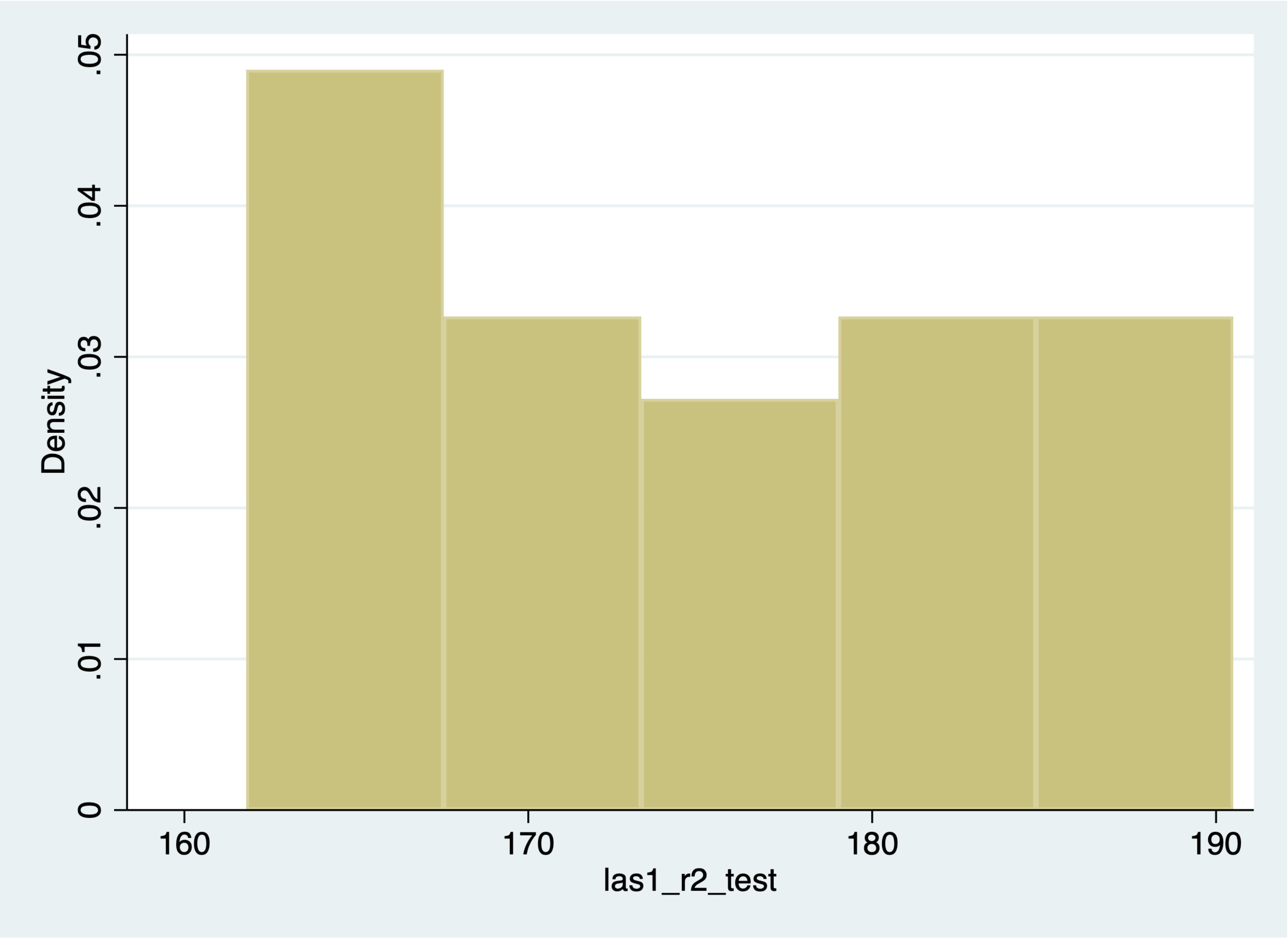

Density
0
.01
.02
.03
.04
.05
160
170
180
190
las1_r2_test

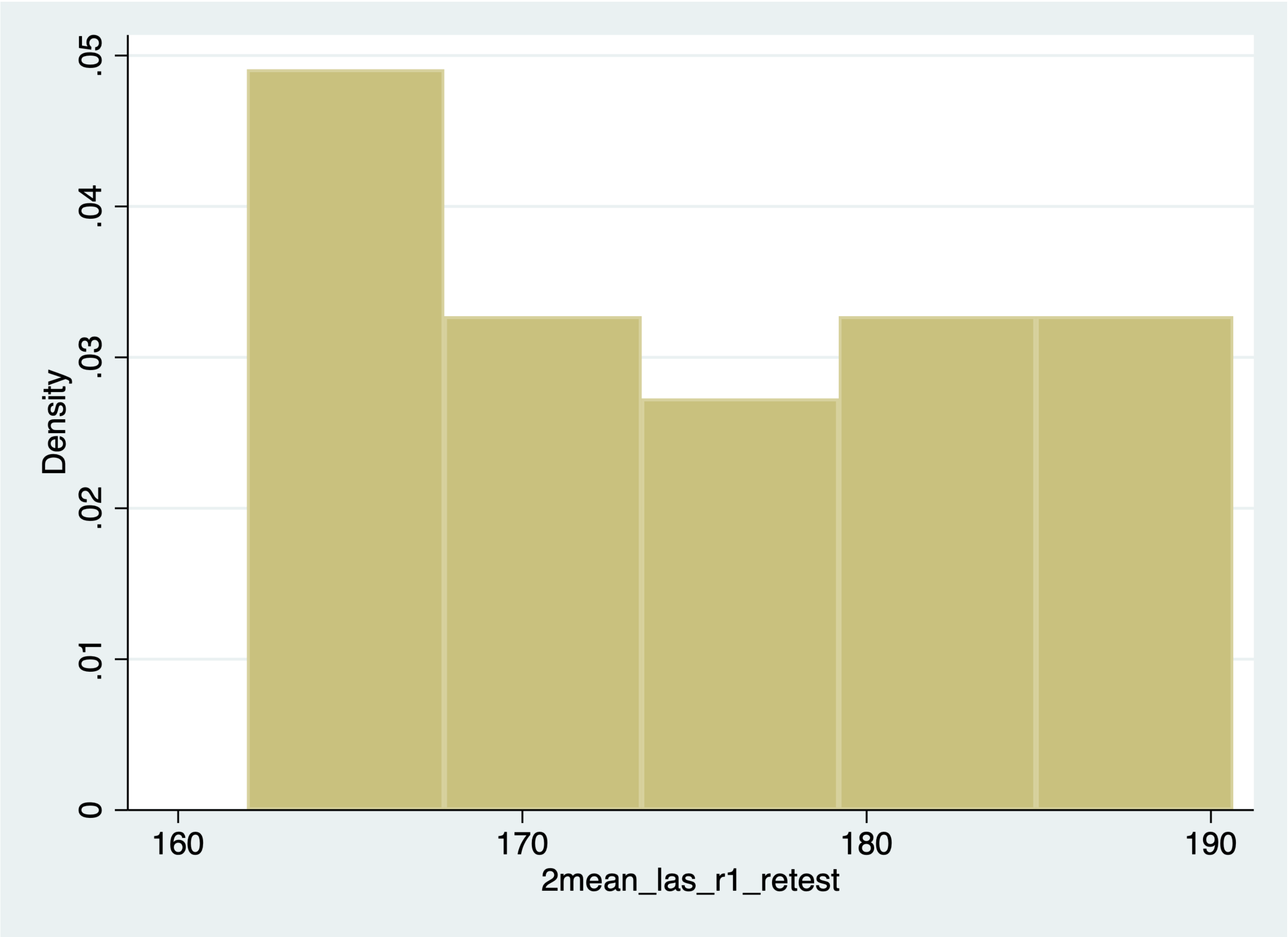
.05
.04
.03
.02
.01
0
Density
160
170
180
190
2mean_las_r1_retest

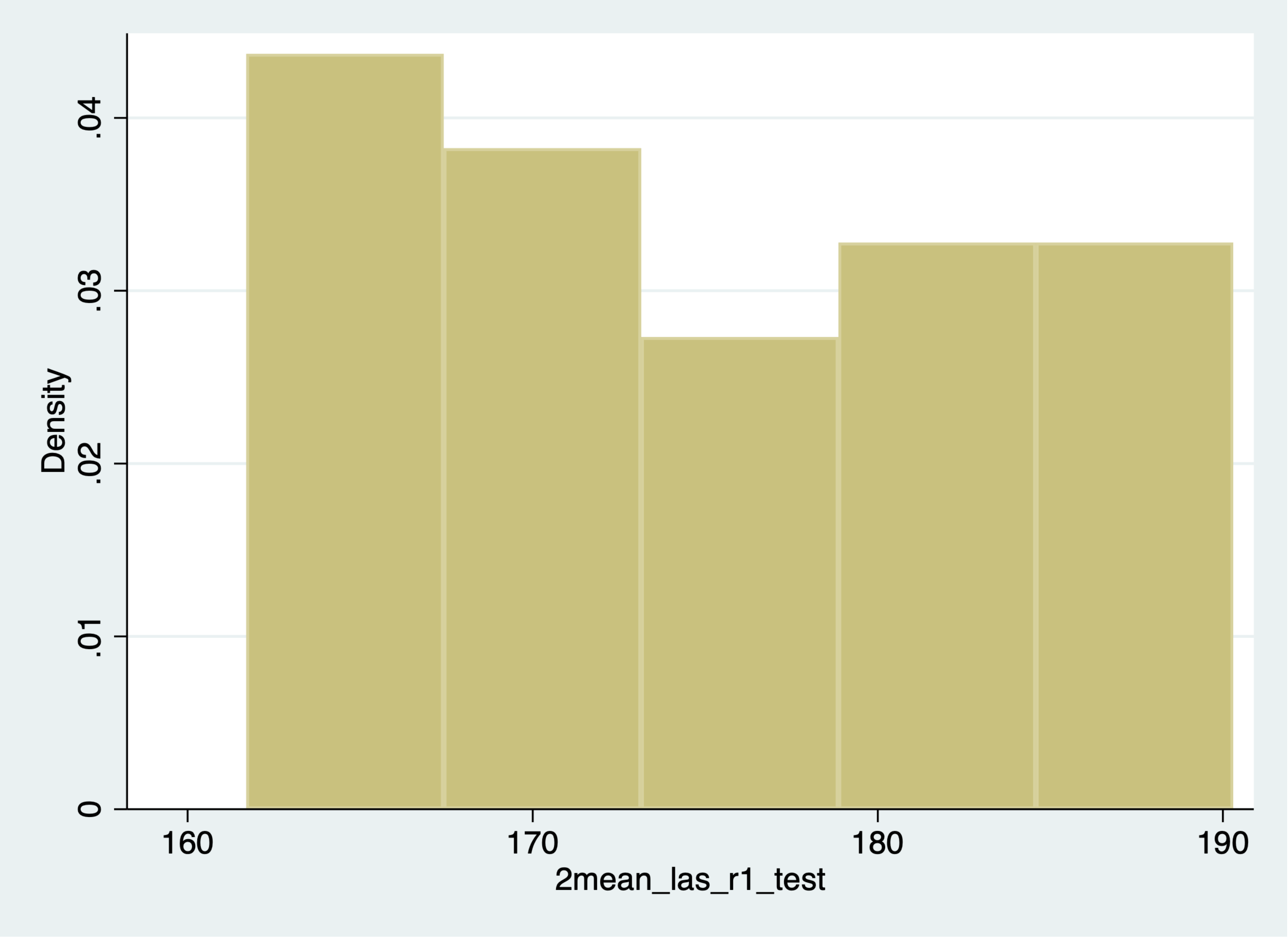

Density
0
.01
.02
.03
.04
160
170
180
190
2mean_las_r1_test

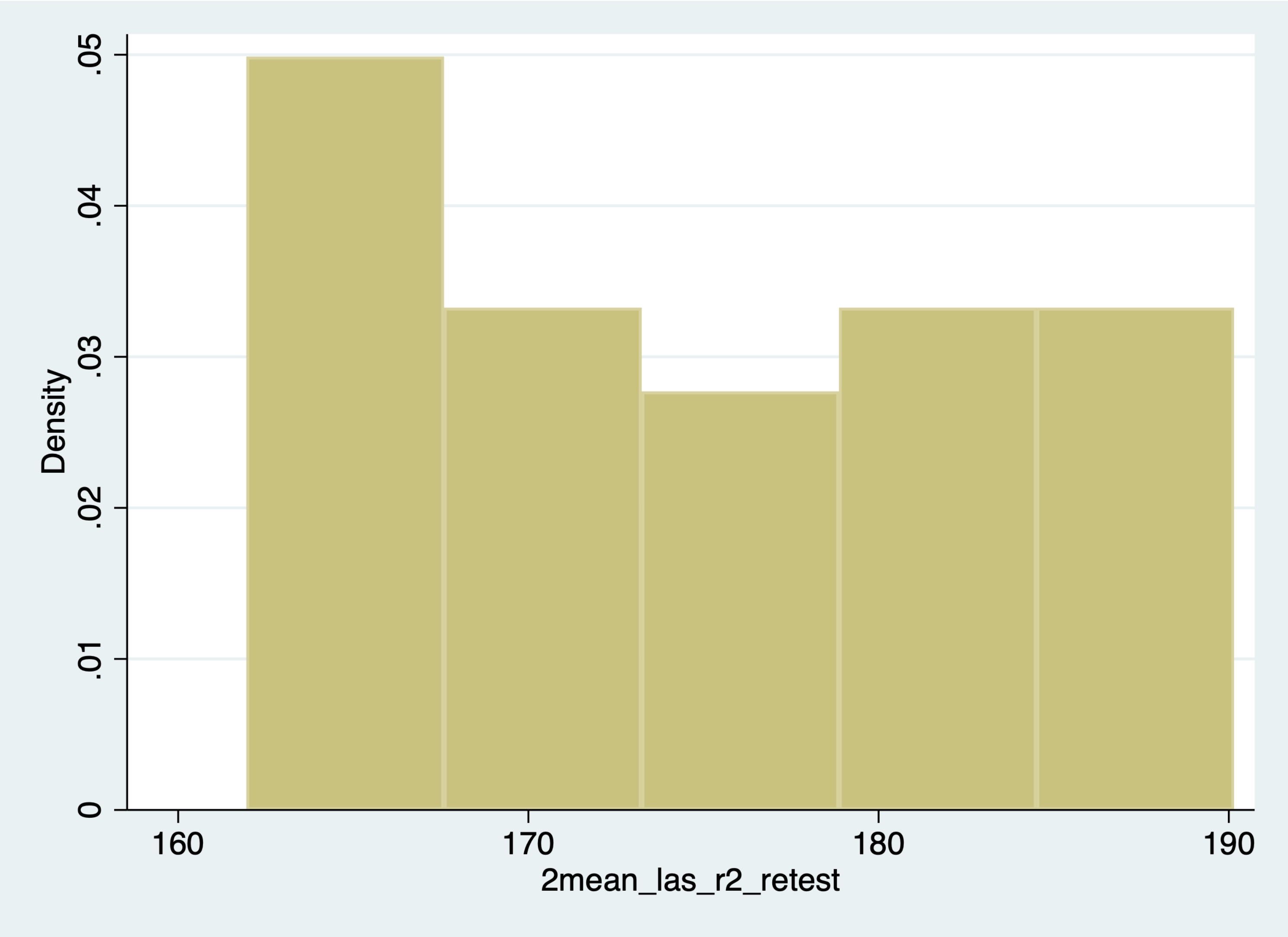
Density
0
.01
.02
.03
.04
.05
160
170
180
190
2mean_las_r2_retest

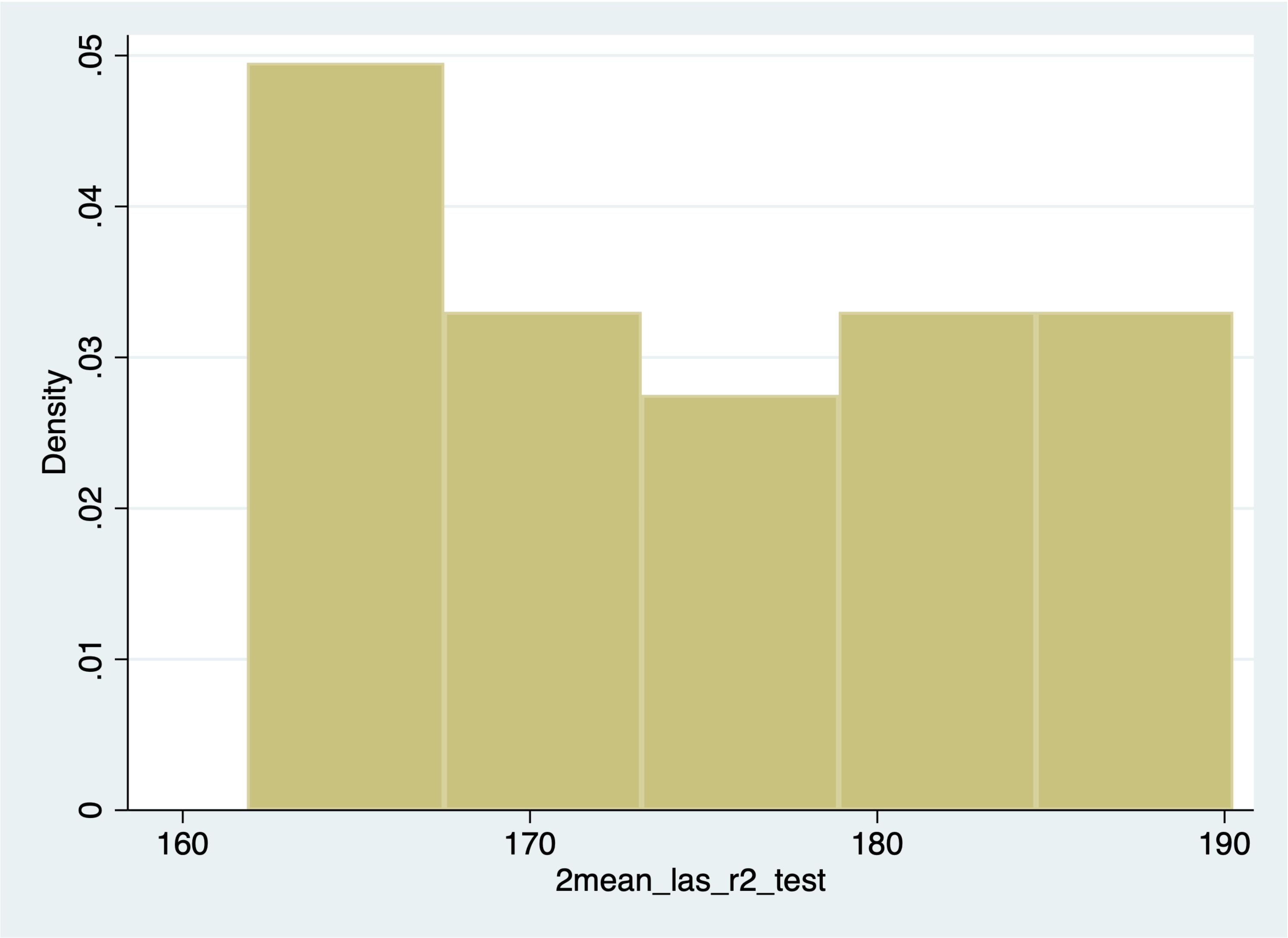
Density
0
.01
.02
.03
.04
.05
160
170
180
190
2mean_las_r2_test

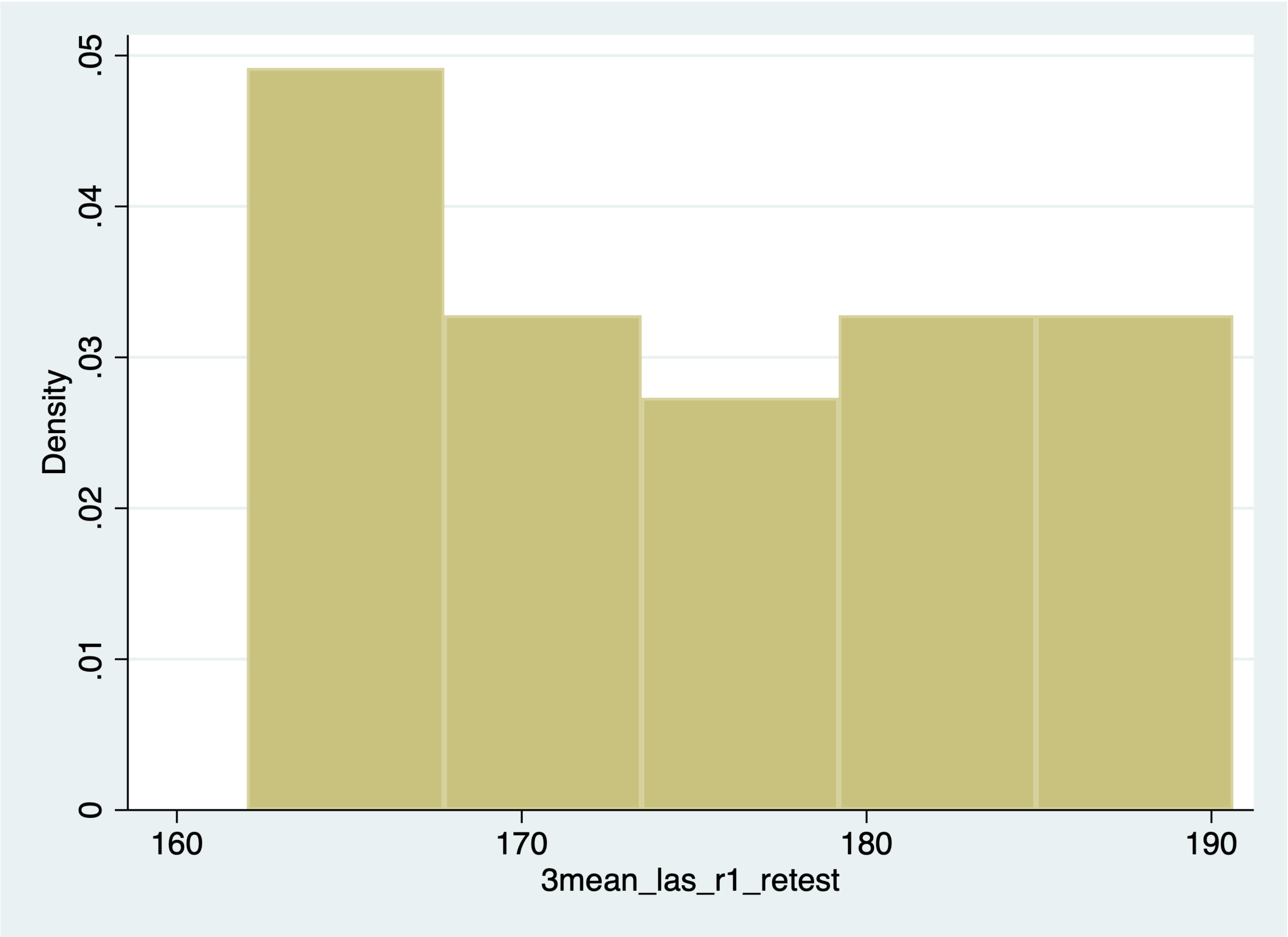
Density
0
.01
.02
.03
.04
.05
160
170
180
190
3mean_las_r1_retest

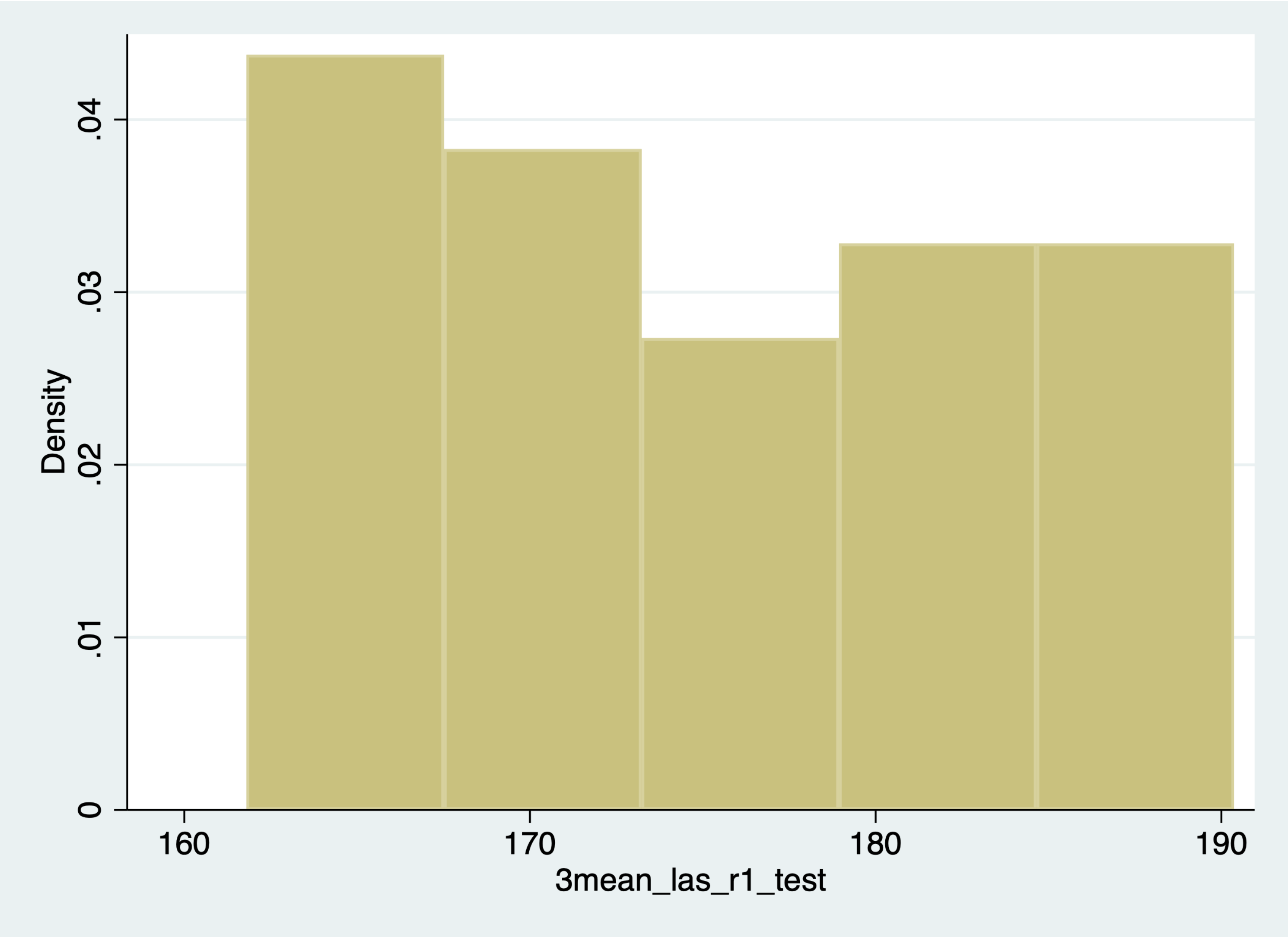
Density
.04
.03
.02
.01
0
160
170
180
190
3mean_las_r1_test

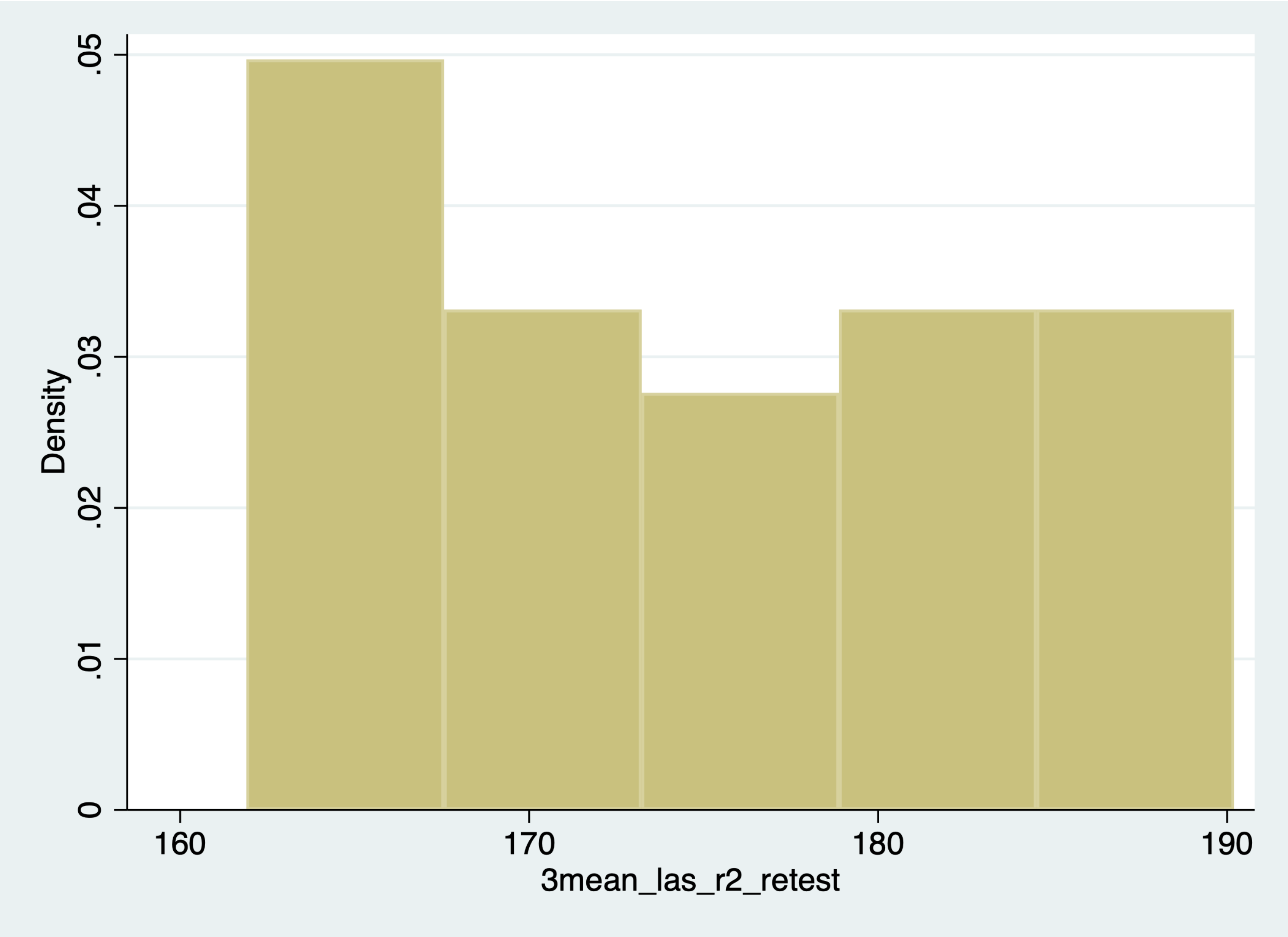

Density
0
.01
.02
.03
.04
.05
160
170
180
190
3mean_las_r2_retest

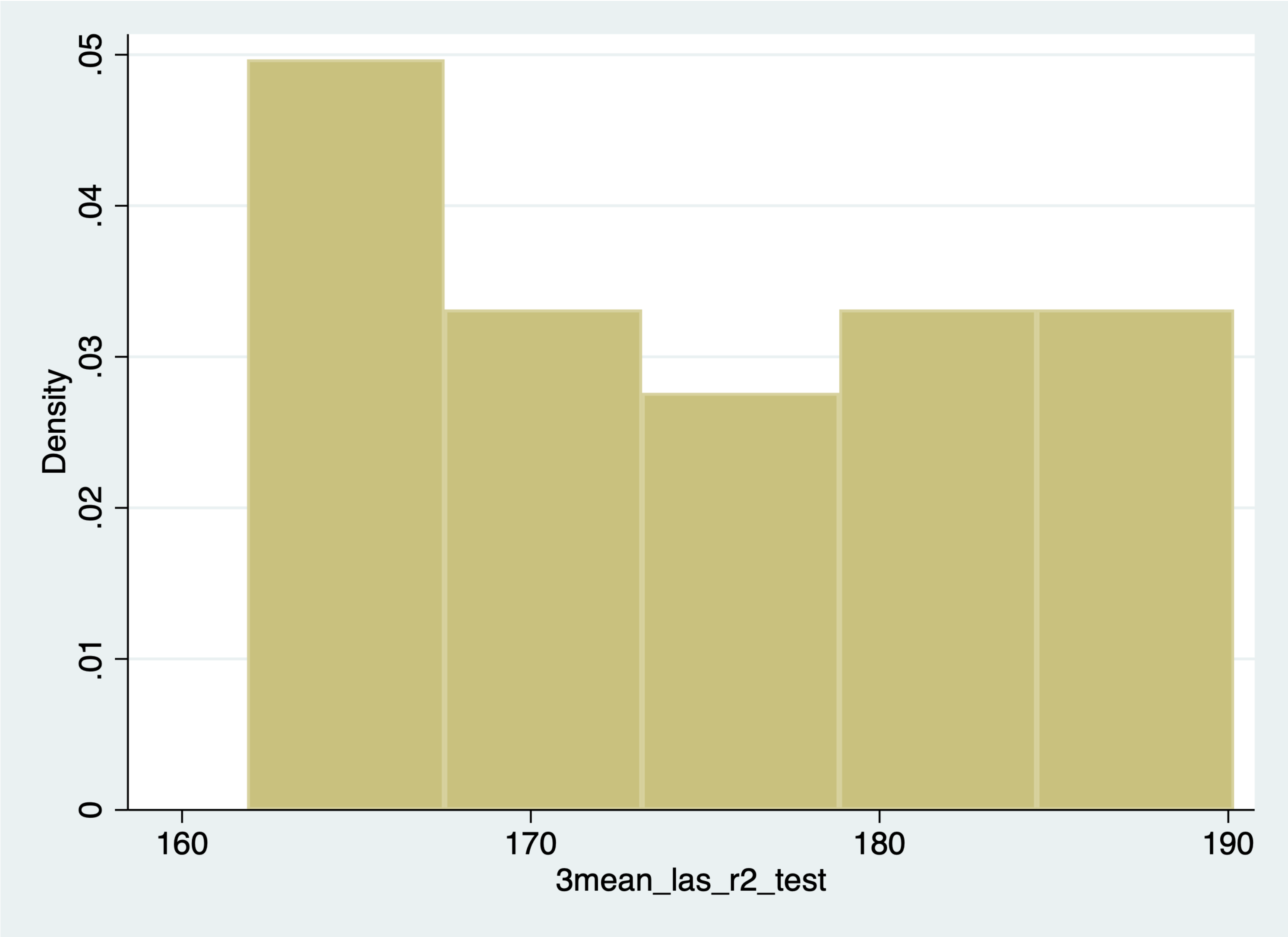
Density
.05
.04
.03
.02
.01
0
160
170
180
190
3mean_las_r2_test

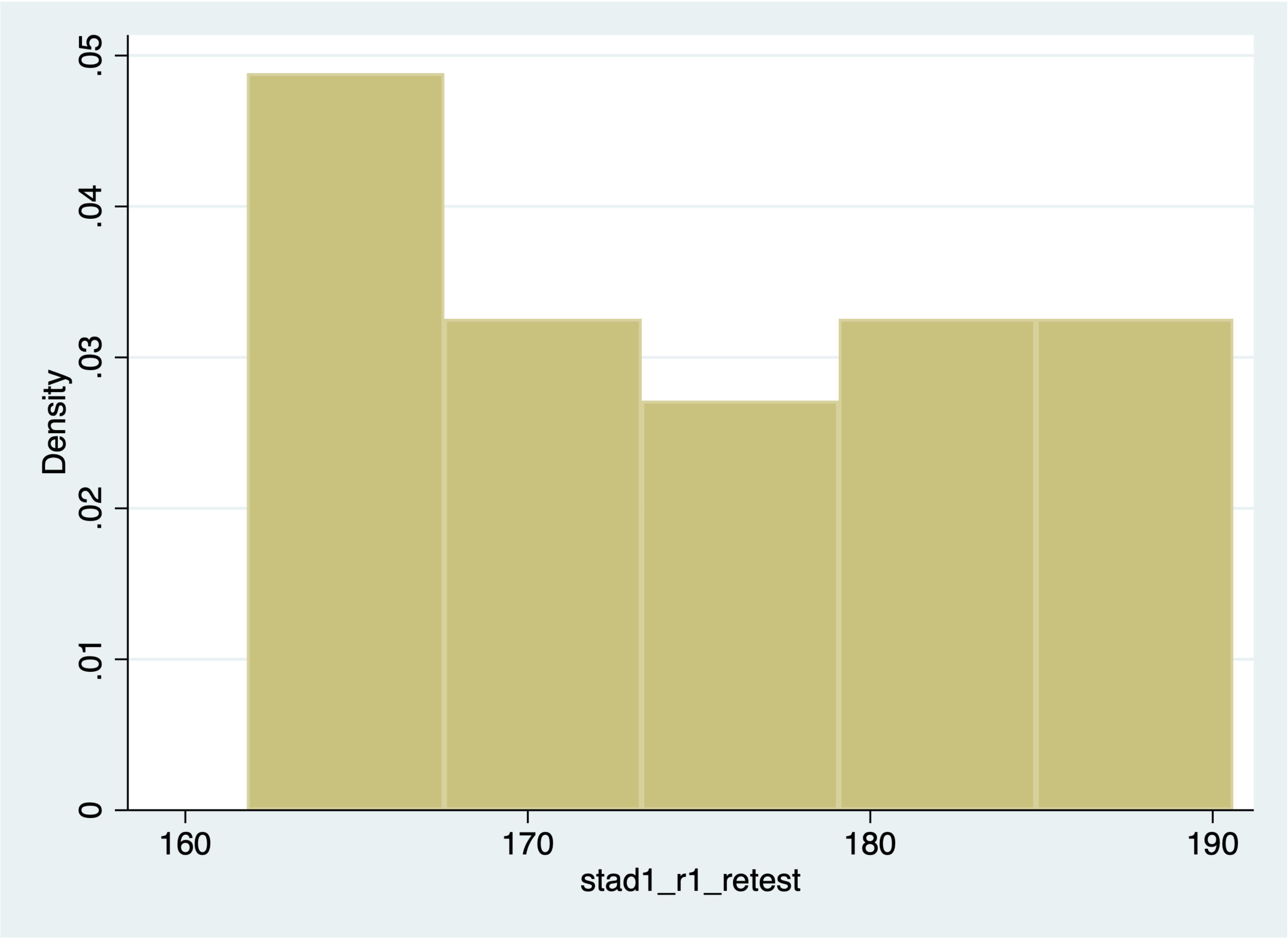

Density
.05
.04
.03
.02
.01
0
160
170
180
190
stad1_r1_retest

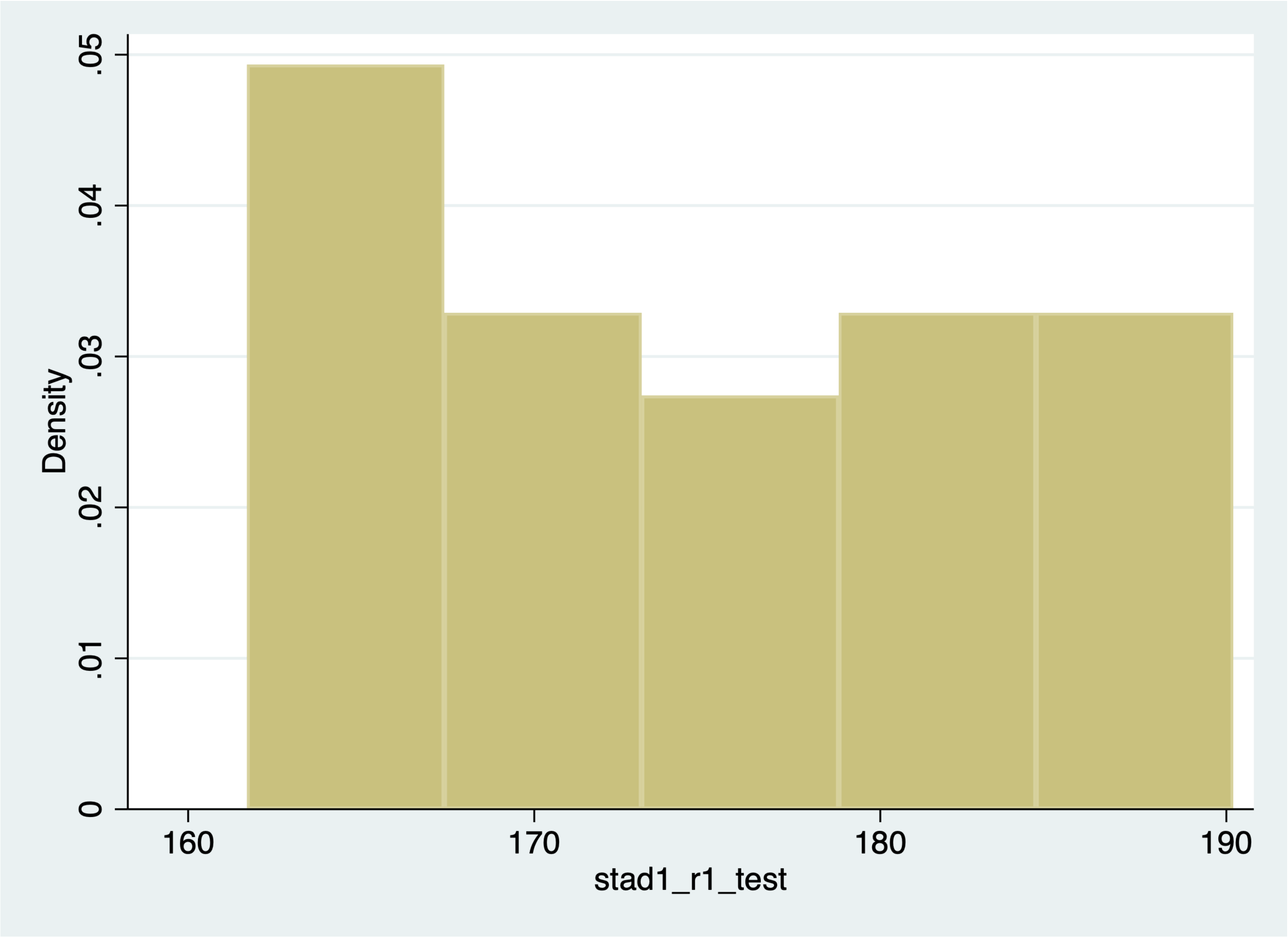

Density
0
.01
.02
.03
.04
.05
160
170
180
190
stad1_r1_test

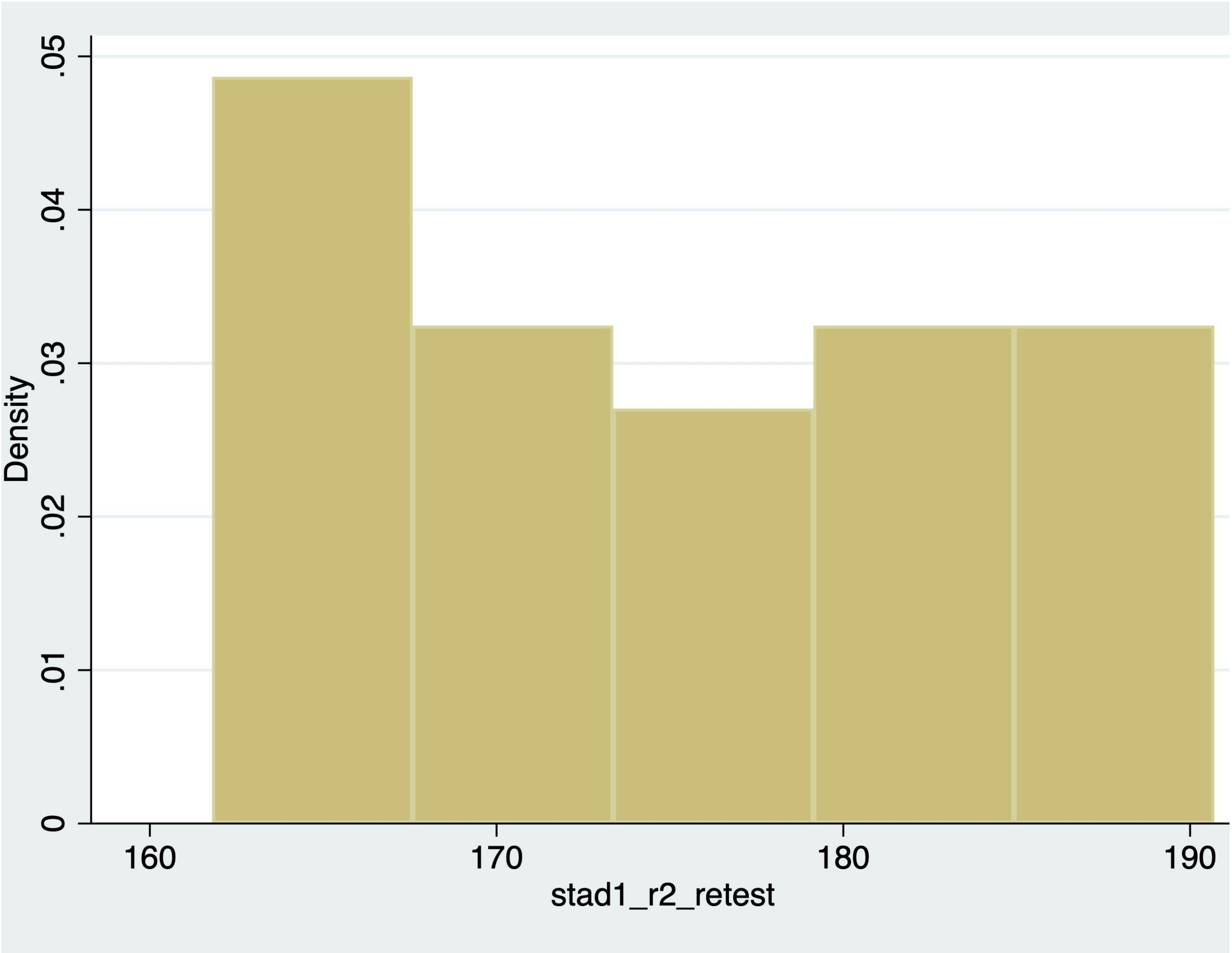

Density
0
.01
.02
.03
.04
.05
160
170
180
190
stad1_r2_retest

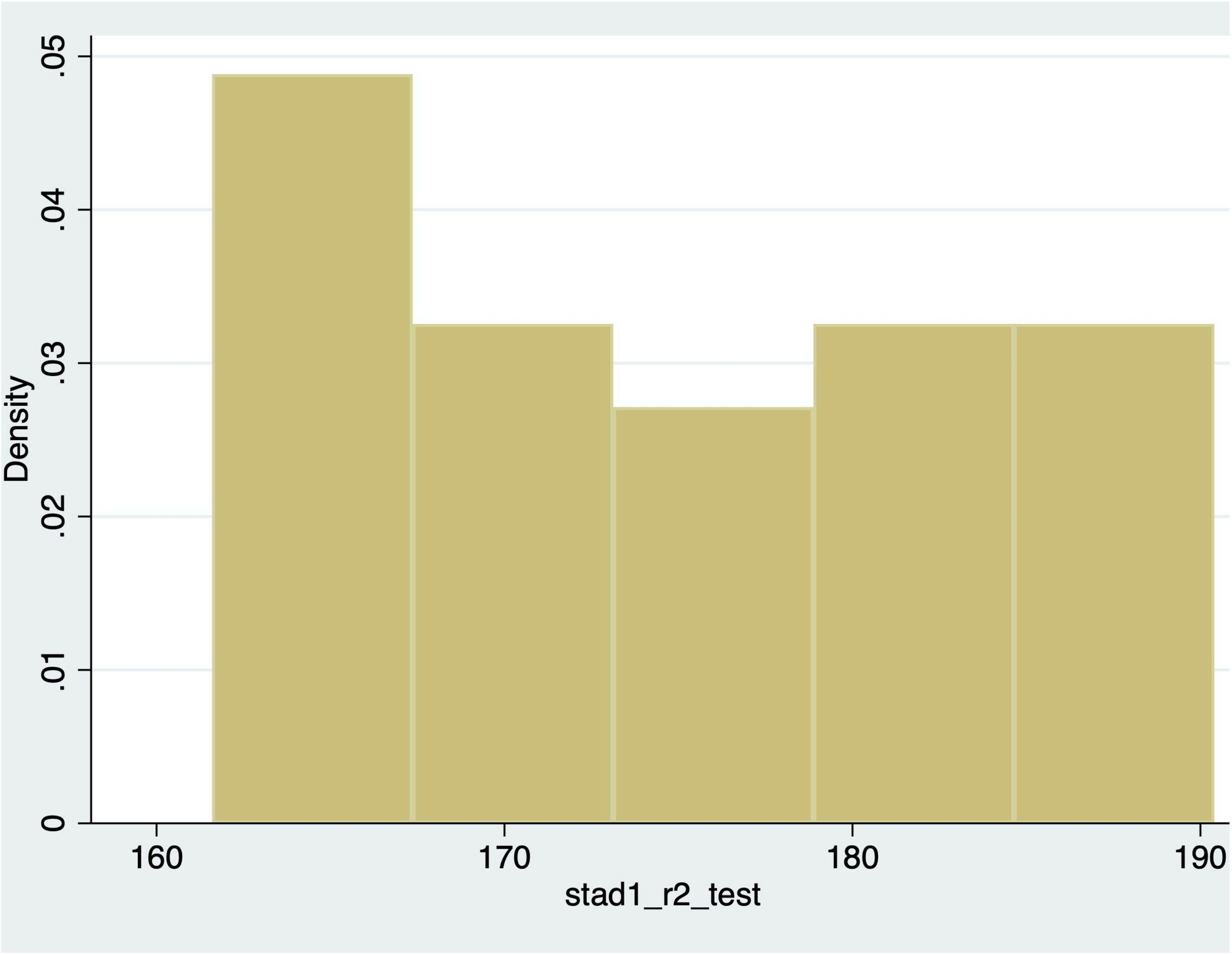

Density
0
.01
.02
.03
.04
.05
160
170
180
190
stad1_r2_test

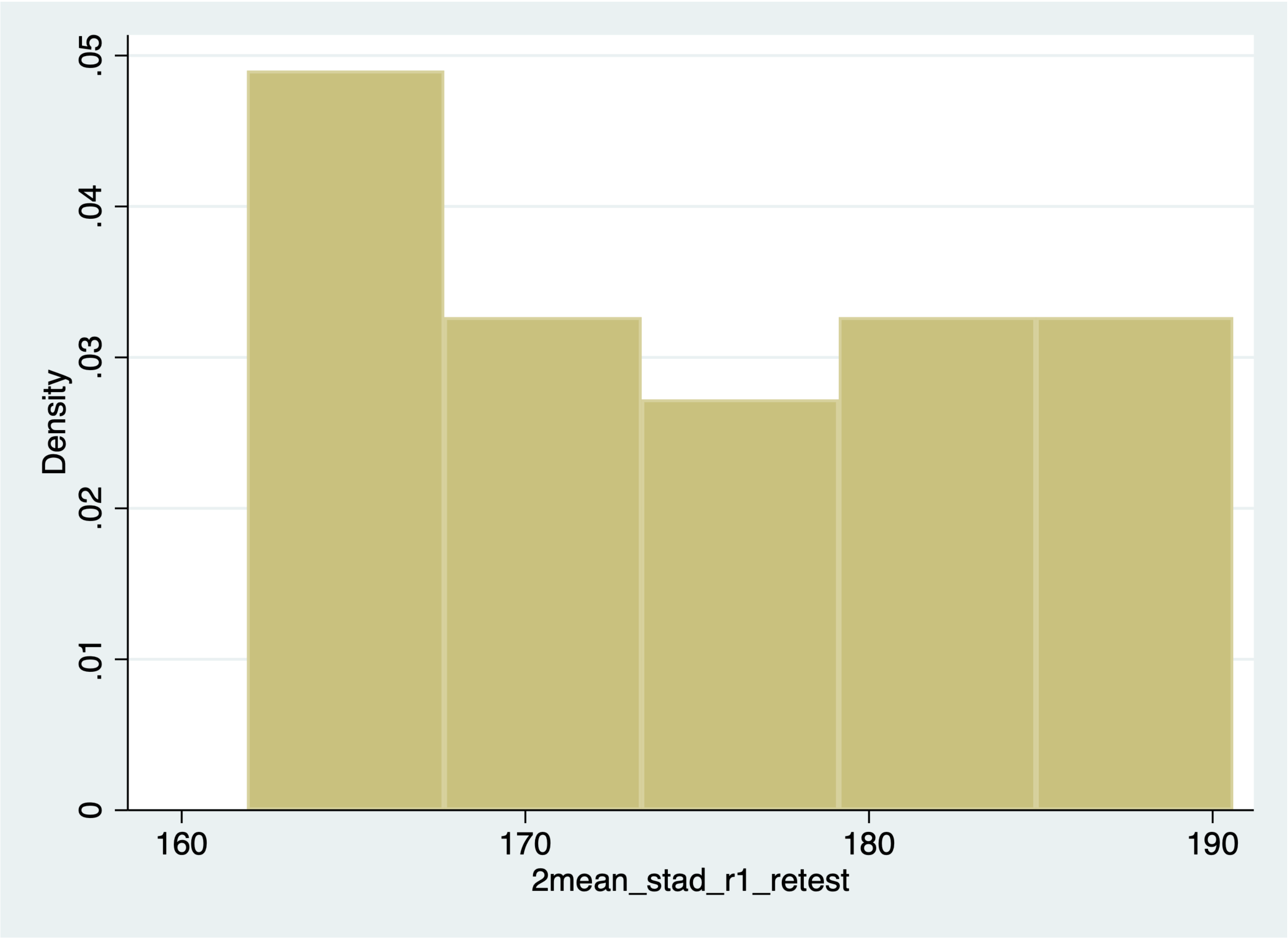

Density
0
.01
.02
.03
.04
.05
160
170
180
190
2mean_stad_r1_retest

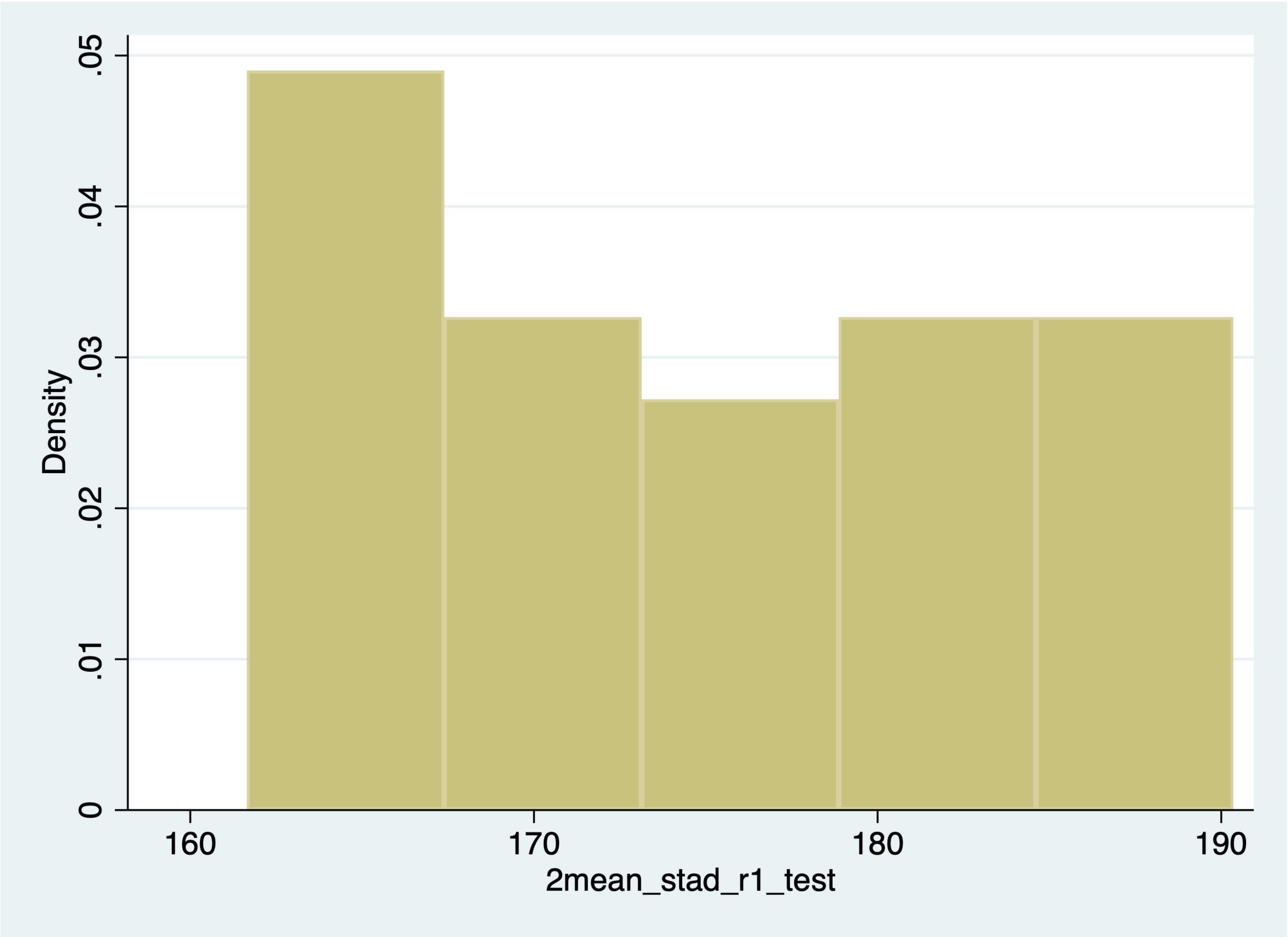
Density
0
.01
.02
.03
.04
.05
160
170
180
190
2mean_stad_r1_test

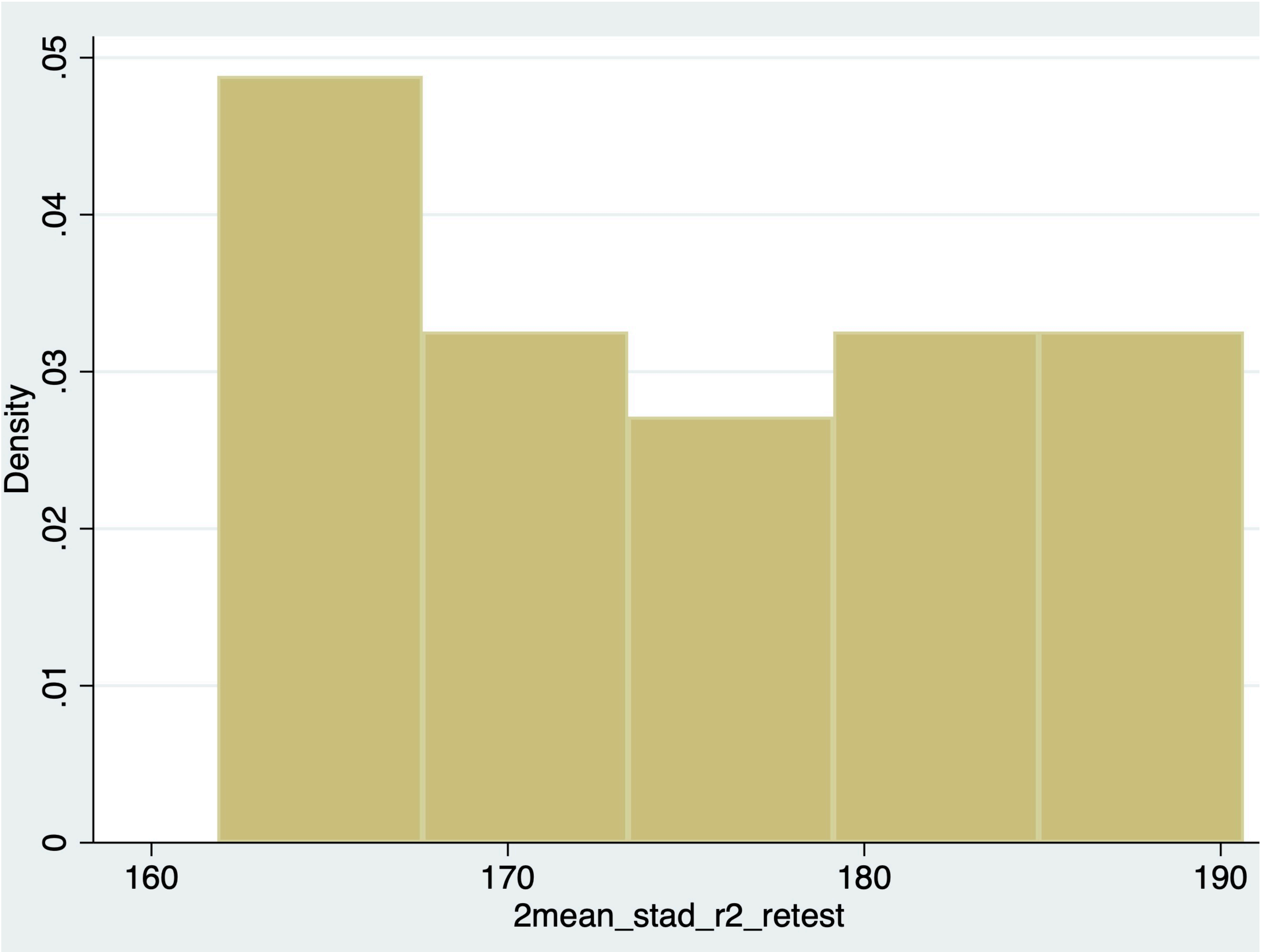

Density
0
.01
.02
.03
.04
.05
160
170
180
190
2mean_stad_r2_retest

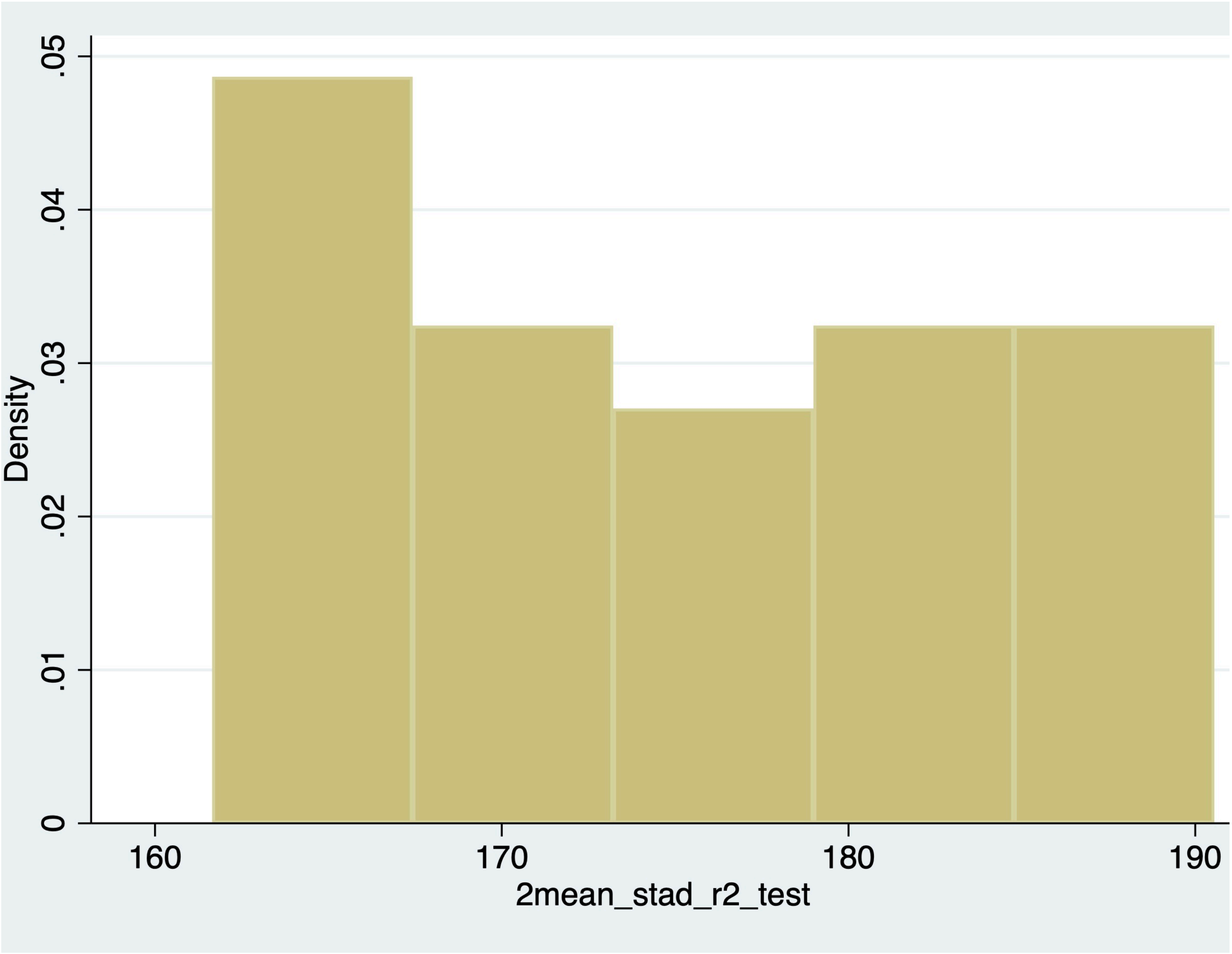

Density
0
.01
.02
.03
.04
.05
160
170
180
190
2mean_stad_r2_test

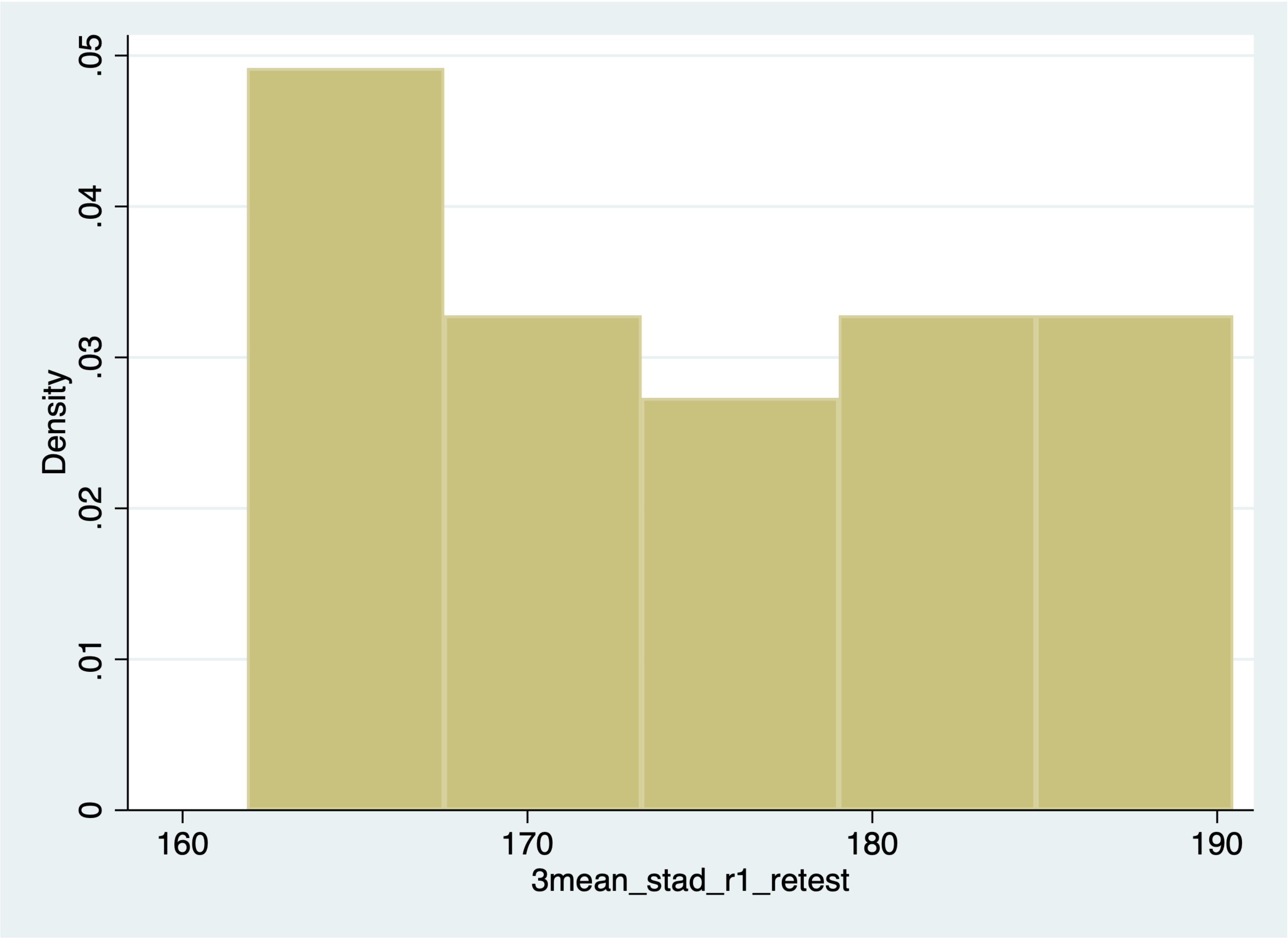

Density
0
.01
.02
.03
.04
.05
160
170
180
190
3mean_stad_r1_retest

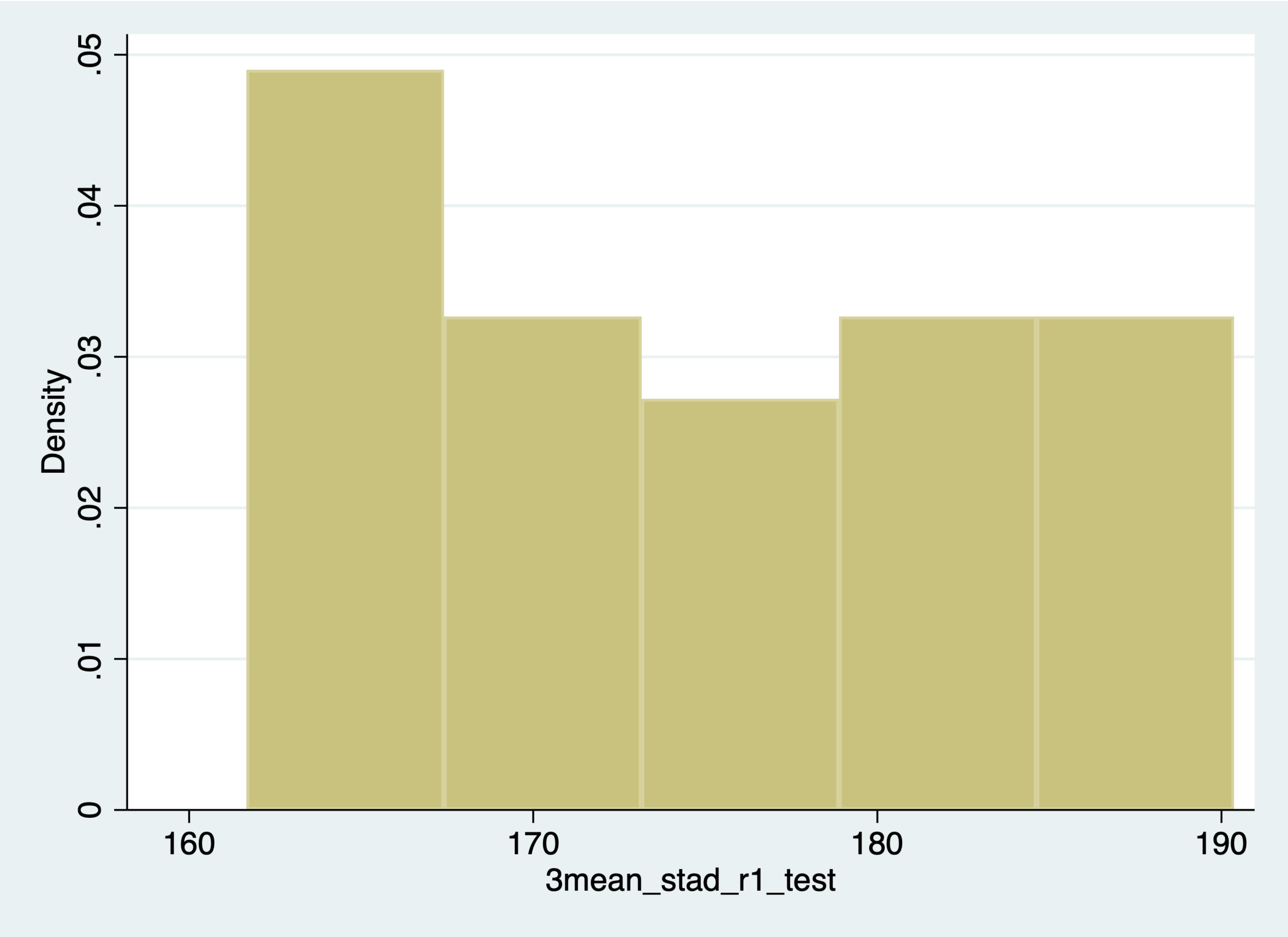

Density
.05
.04
.03
.02
.01
0
160
170
180
190
3mean_stad_r1_test

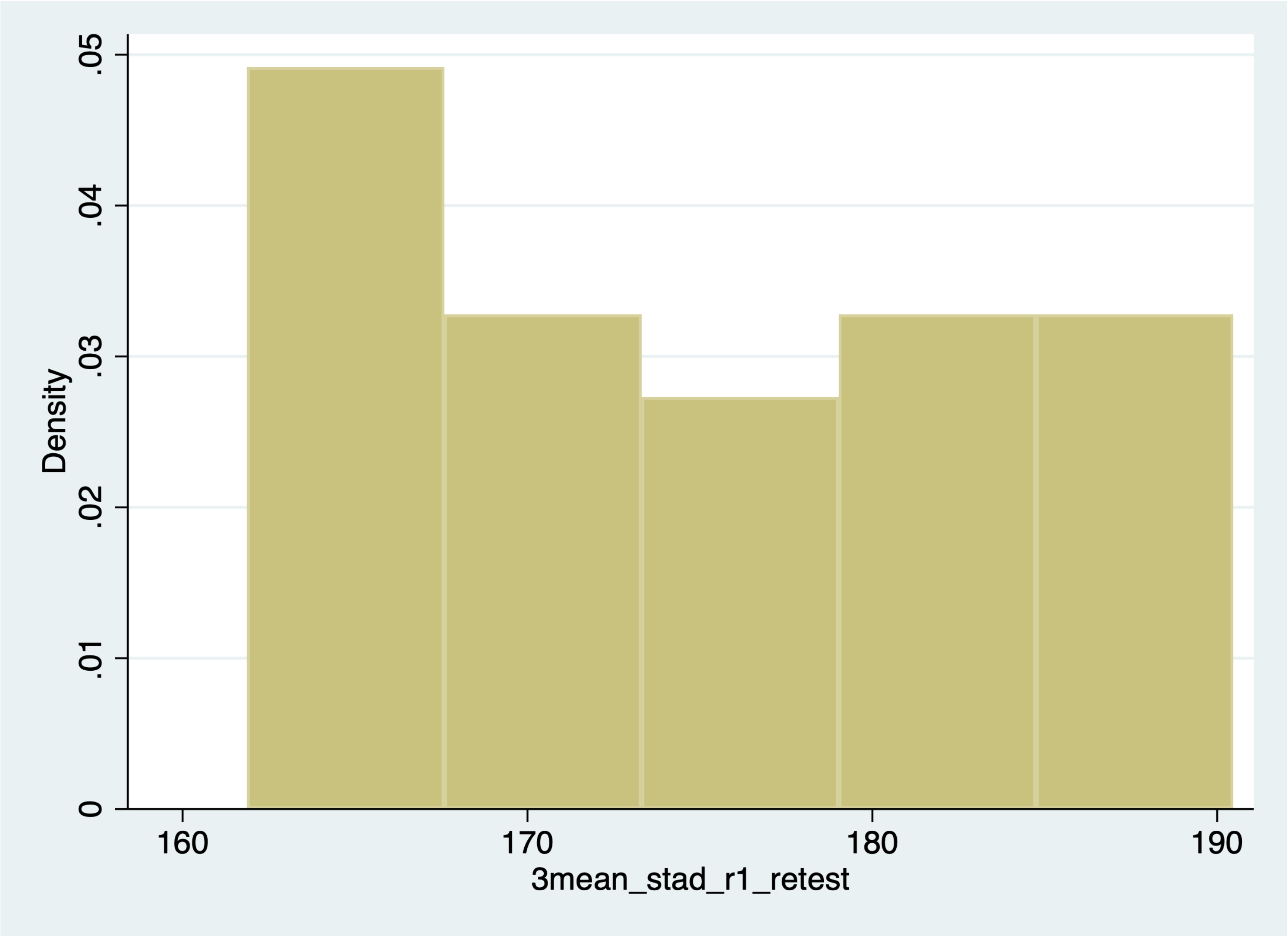

Density
0
.01
.02
.03
.04
.05
160
170
180
190
3mean_stad_r1_retest

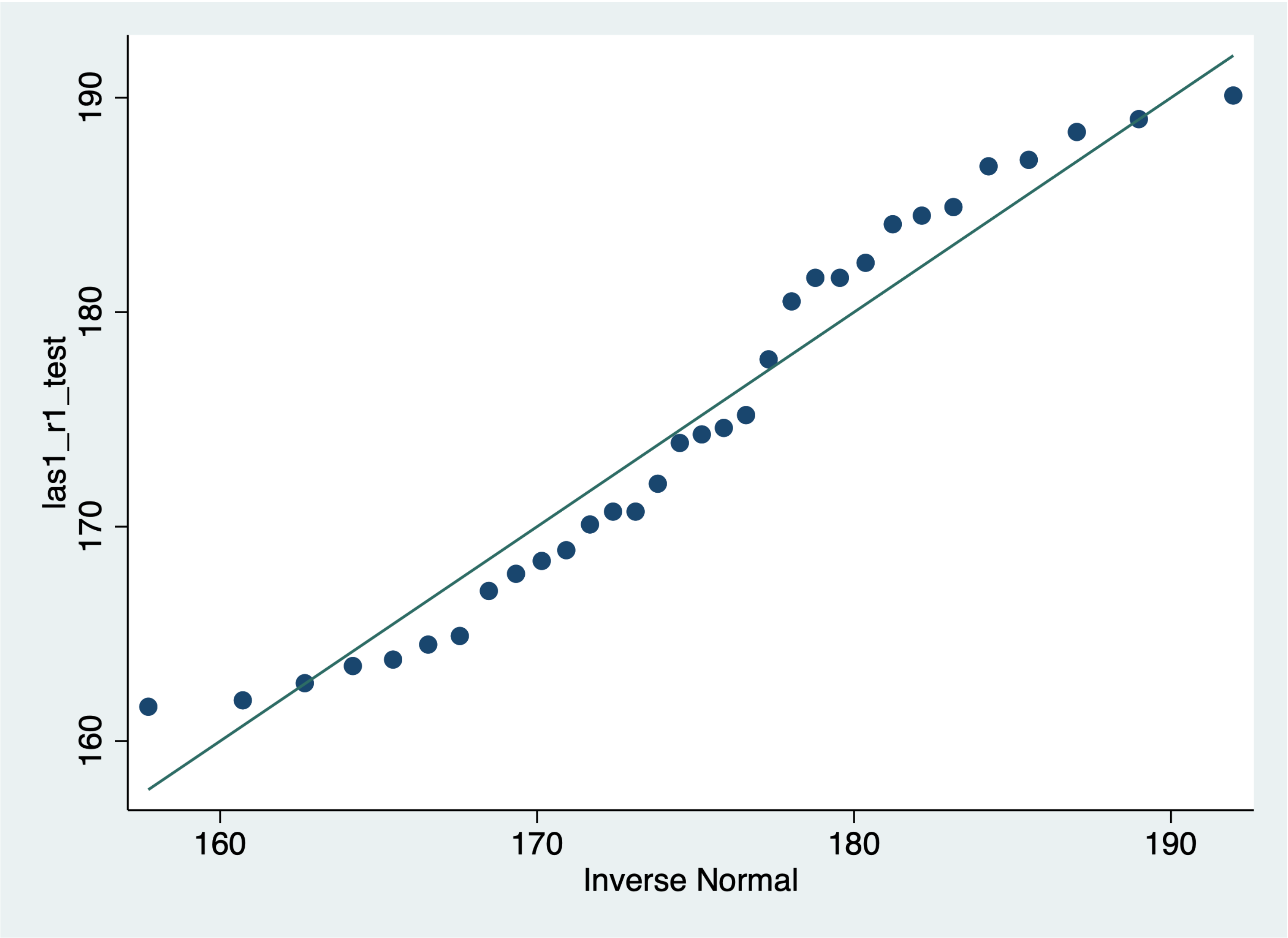

las1_r1_test
160
170
180
190
Inverse Normal

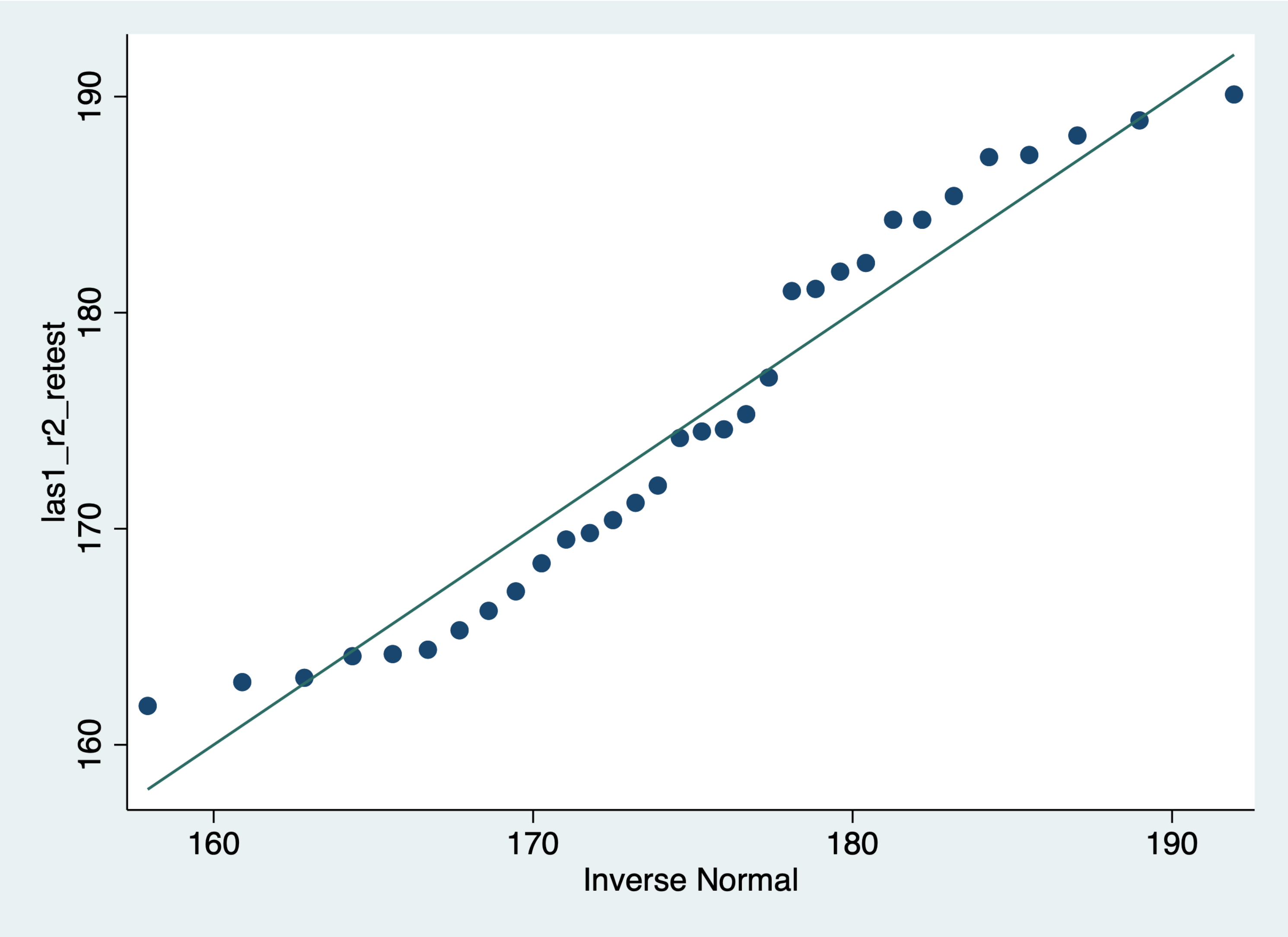

las1_r2_retest
160
170
180
190
160
170
180
190
Inverse Normal

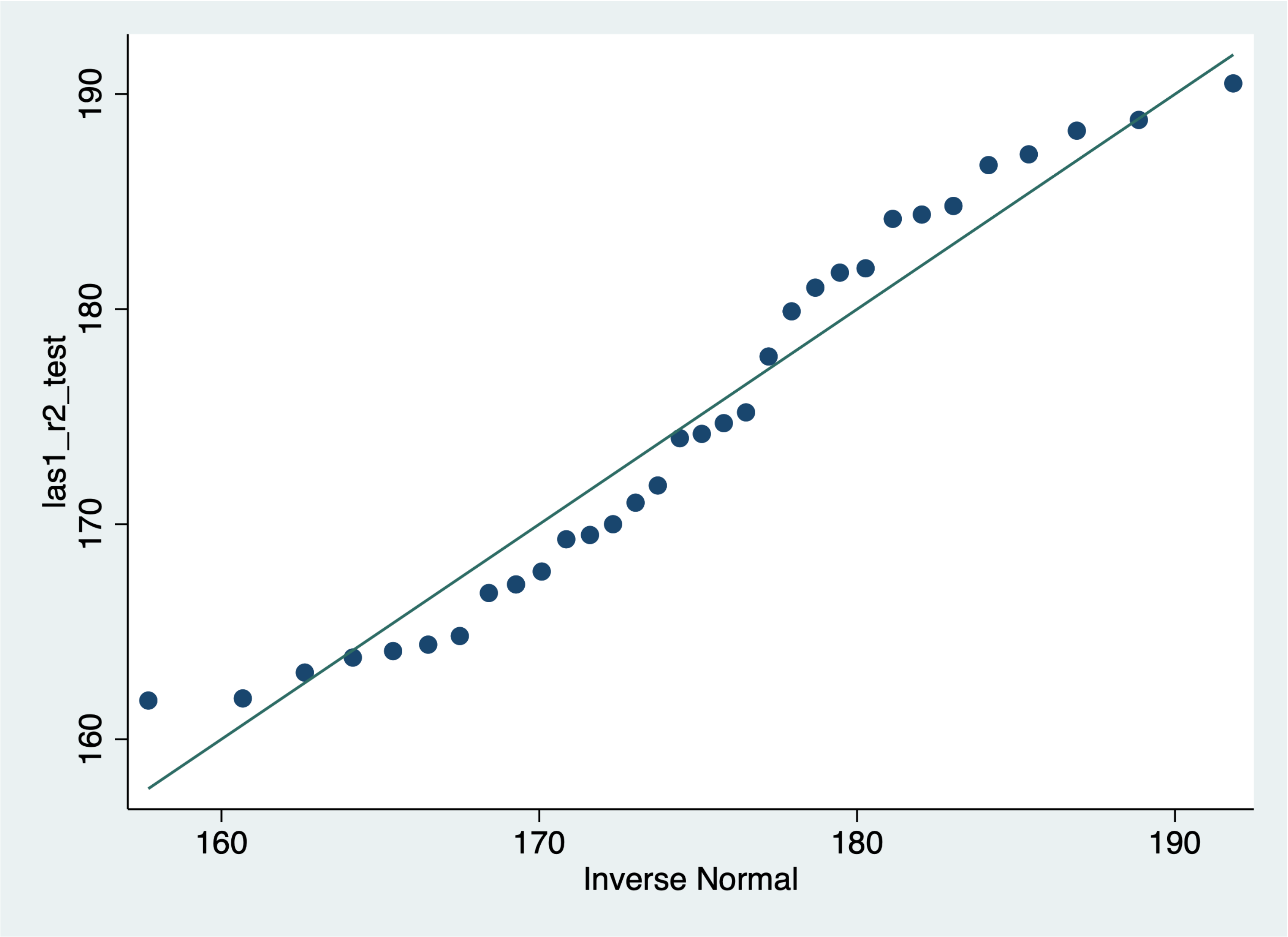

190
180
170
160
las1_r2_test
160
170
180
190
Inverse Normal

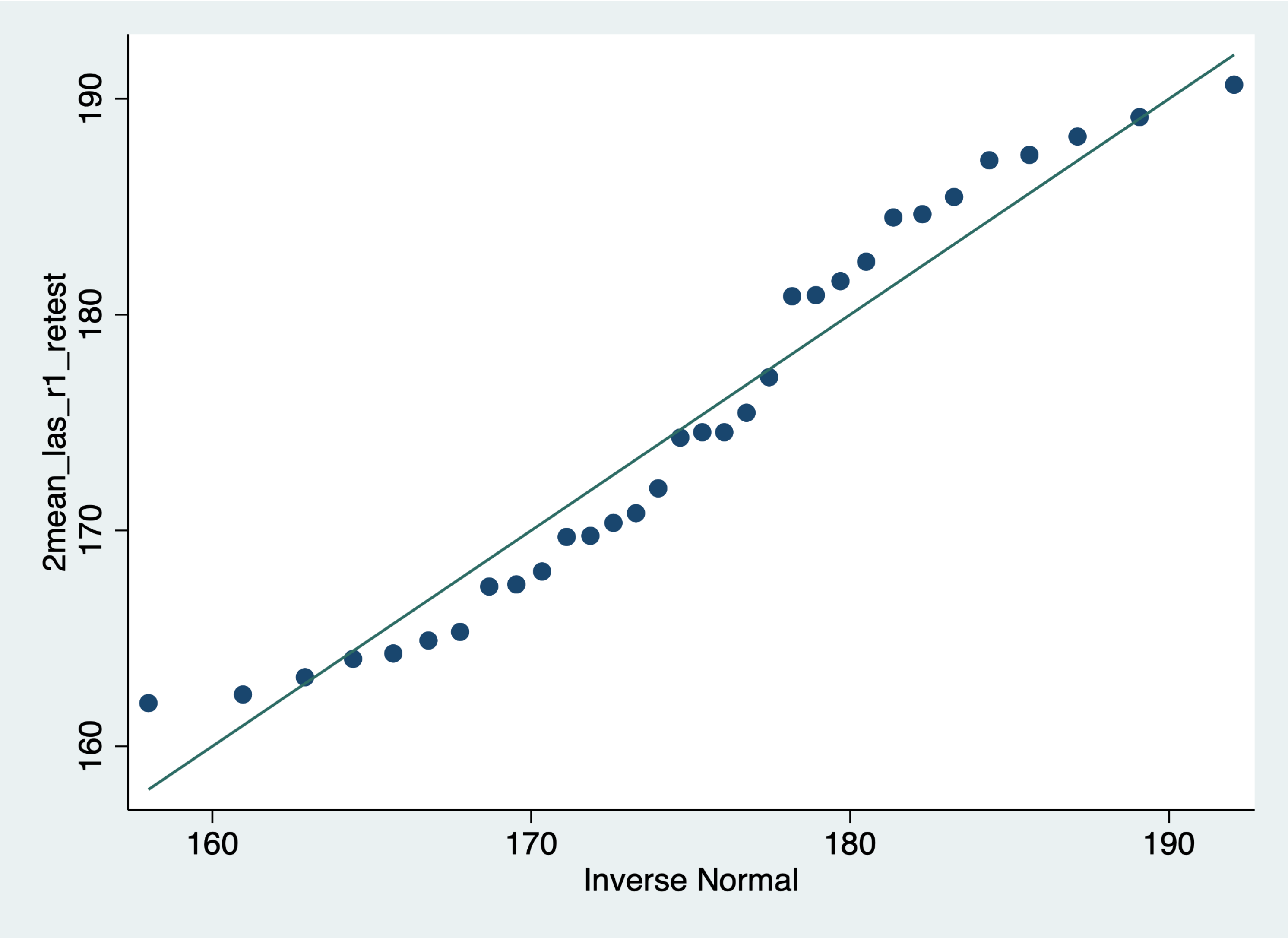
2mean_las_r1_retest
160
170
180
190
160
170
180
190
Inverse Normal

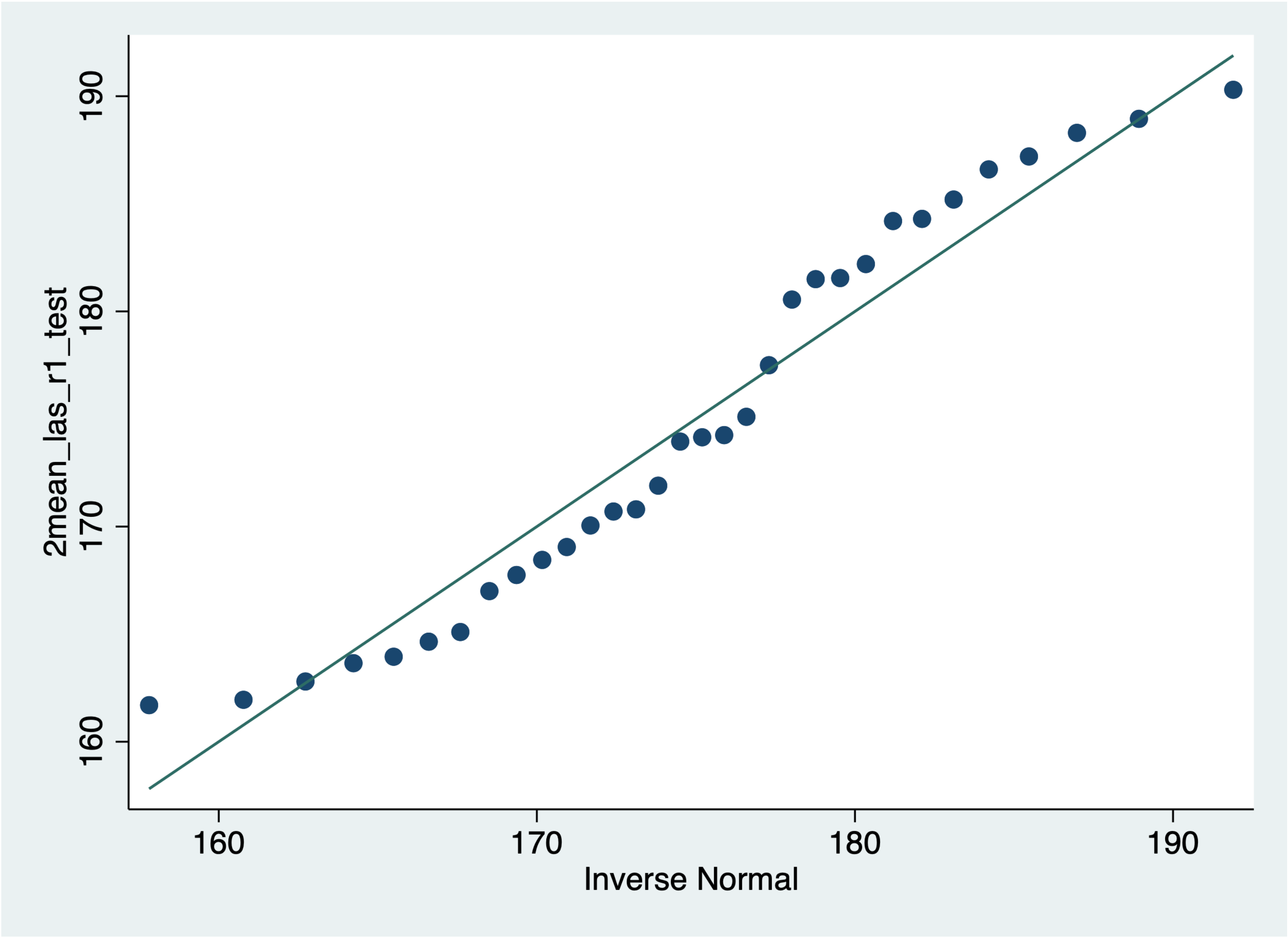

2mean_las_r1_test
160
170
180
190
Inverse Normal

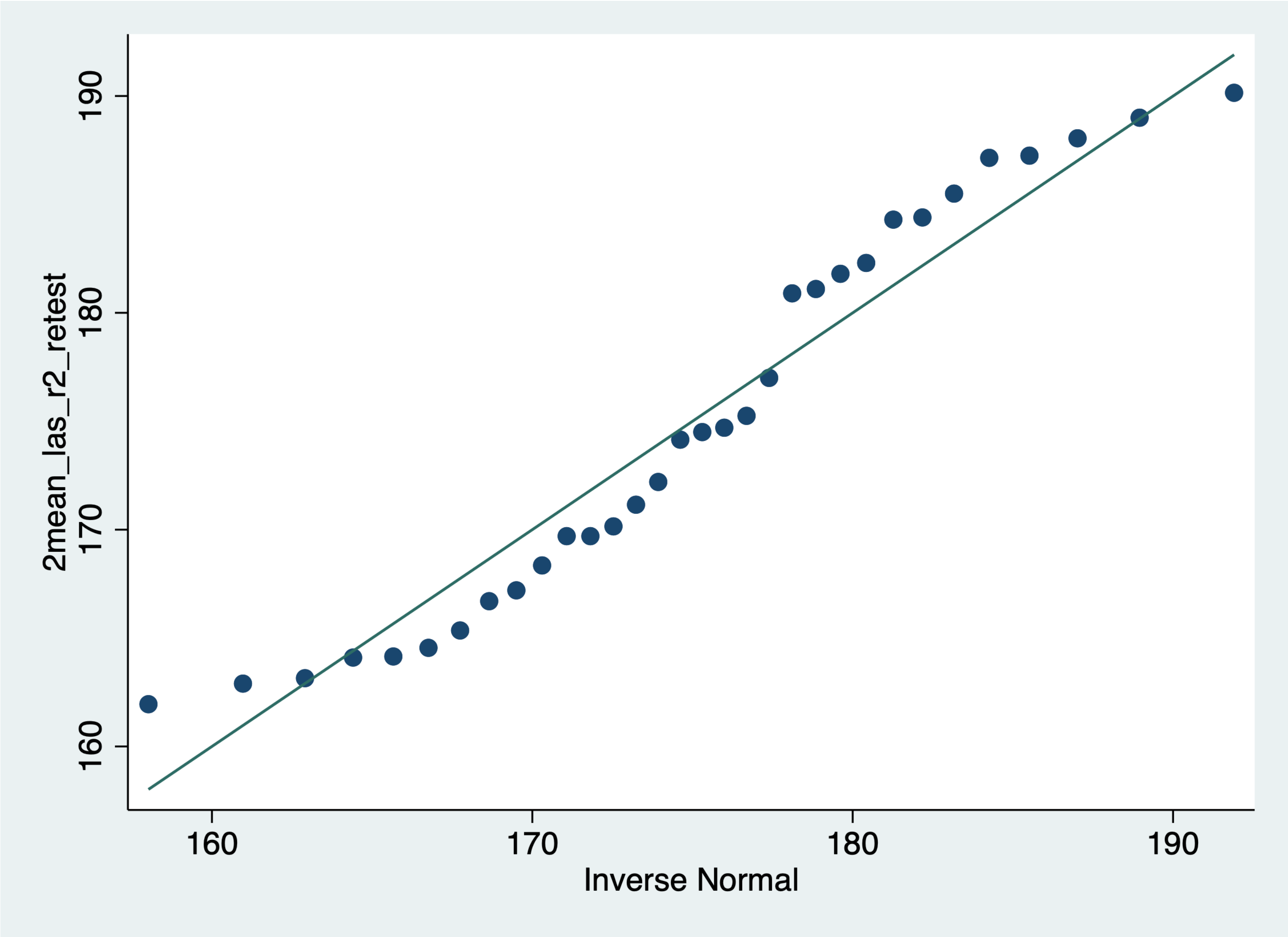

2mean_las_r2_retest
160
170
180
190
160
170
180
190
Inverse Normal

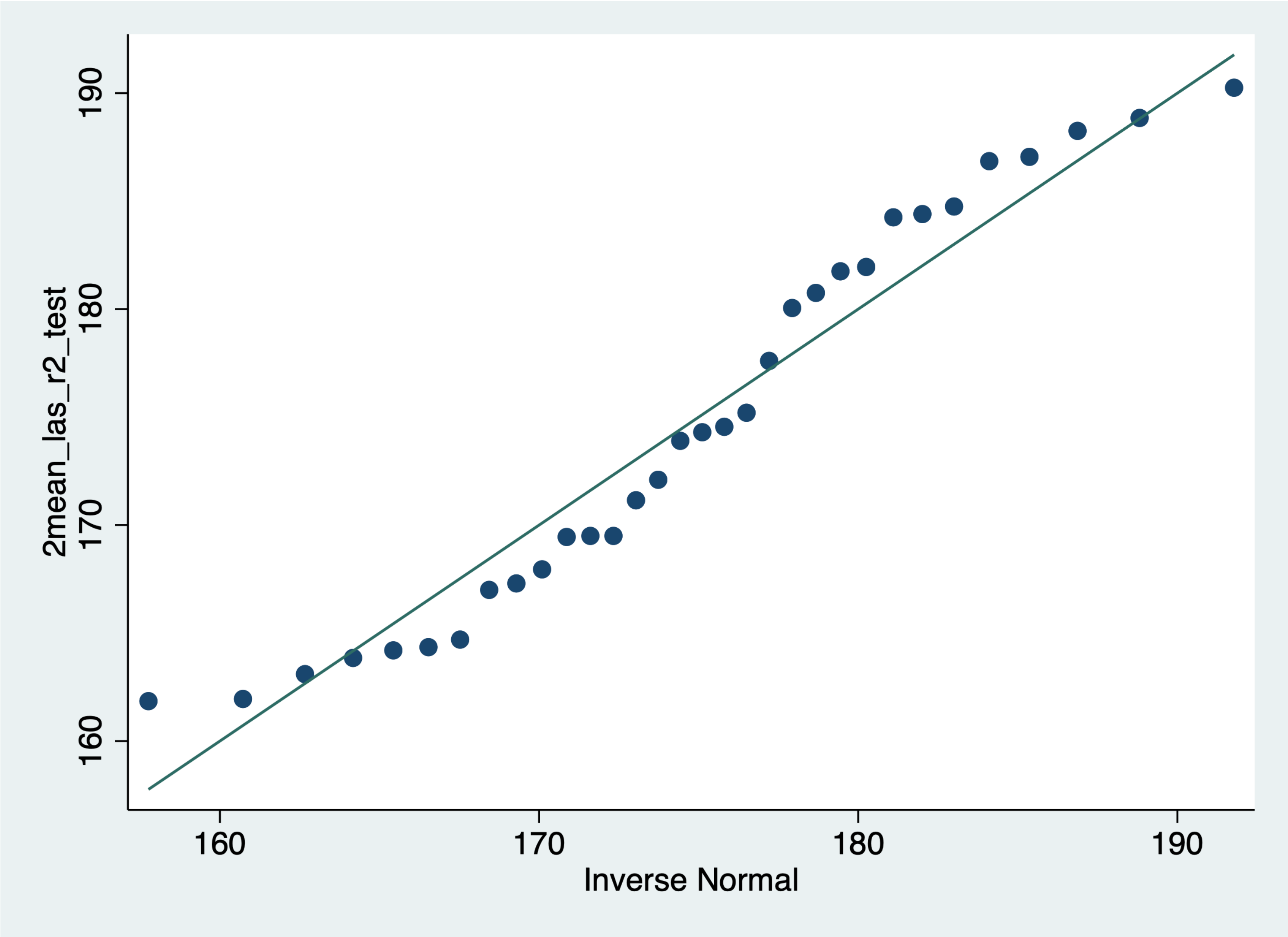
2mean_las_r2_test
190
180
170
160
160
170
180
190
Inverse Normal

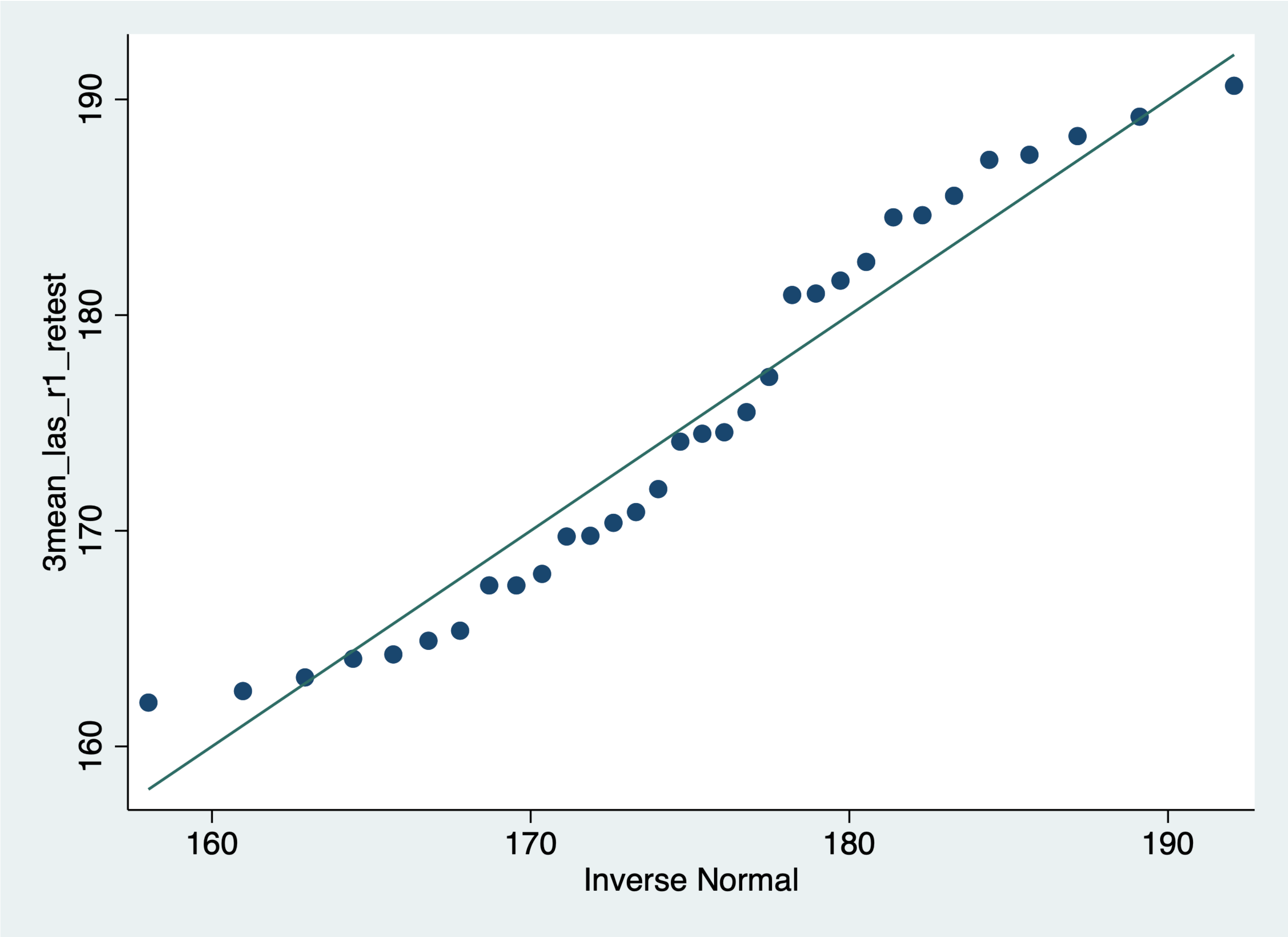
3mean_las_r1_retest
160
170
180
190
160
170
180
190
Inverse Normal

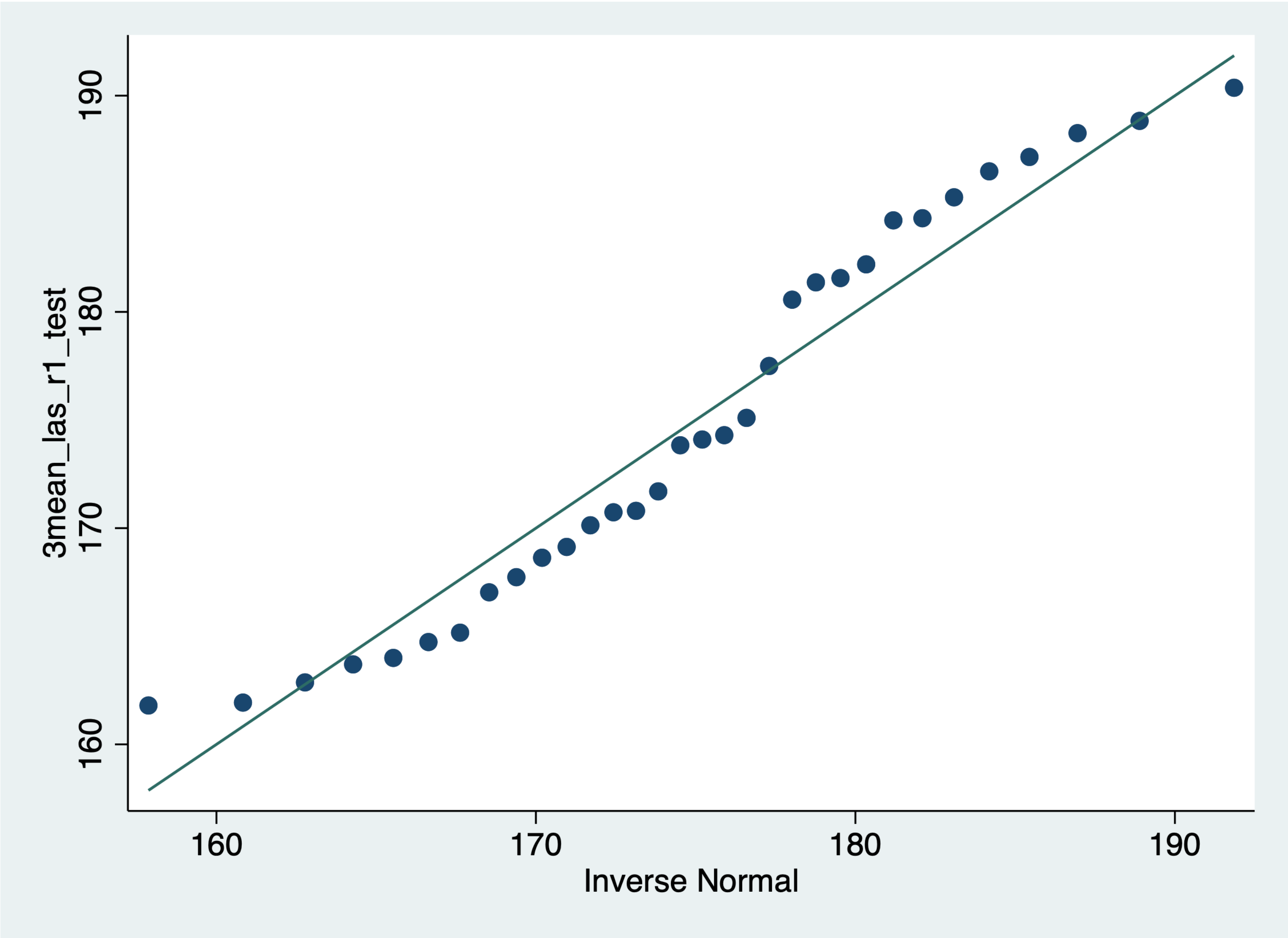

3mean_las_r1_test
190
180
170
160
160
170
180
190
Inverse Normal

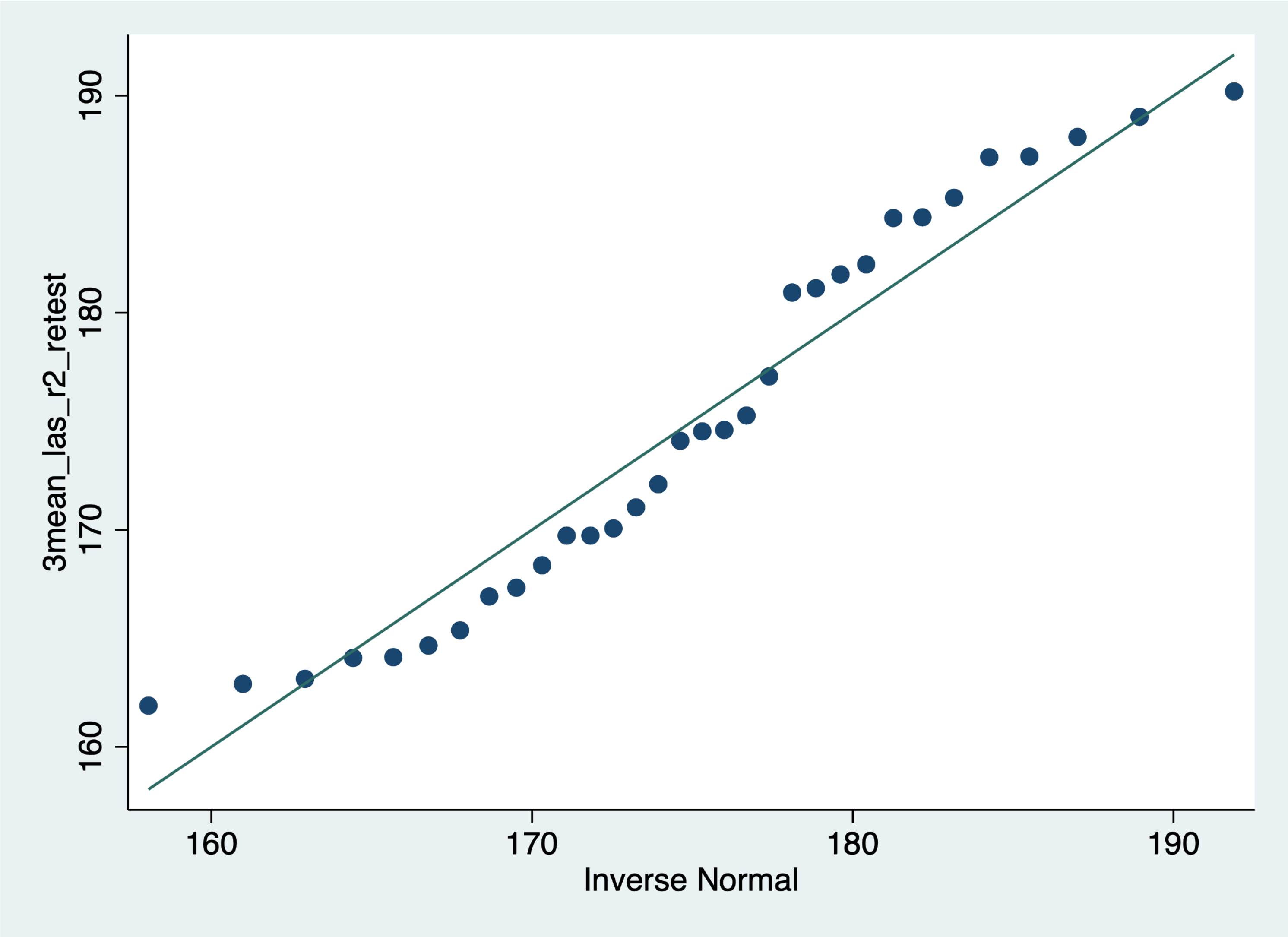

190
180
170
160
3mean_las_r2_retest
160
170
180
190
Inverse Normal

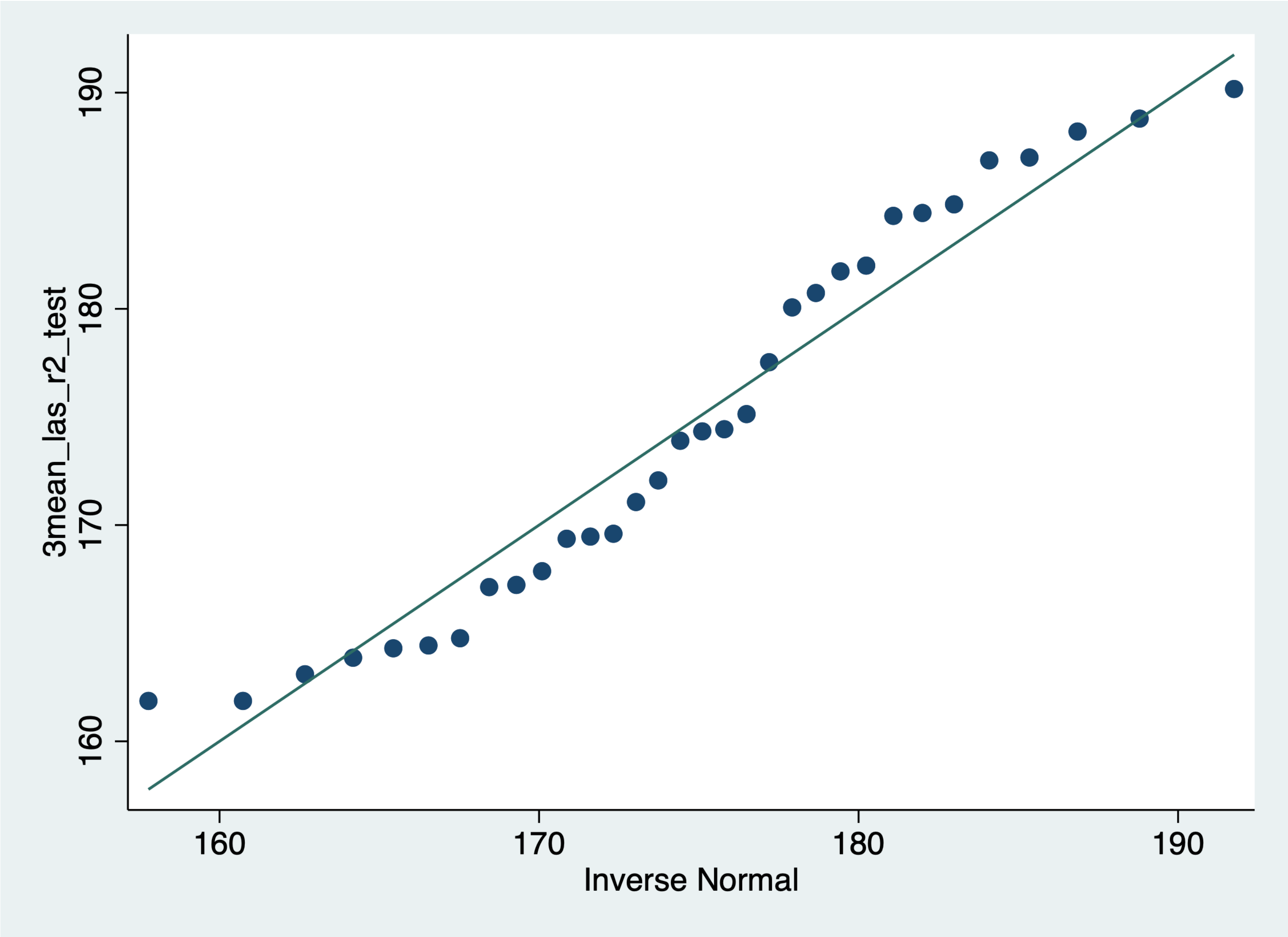

3mean_las_r2_test
160
170
180
190
Inverse Normal

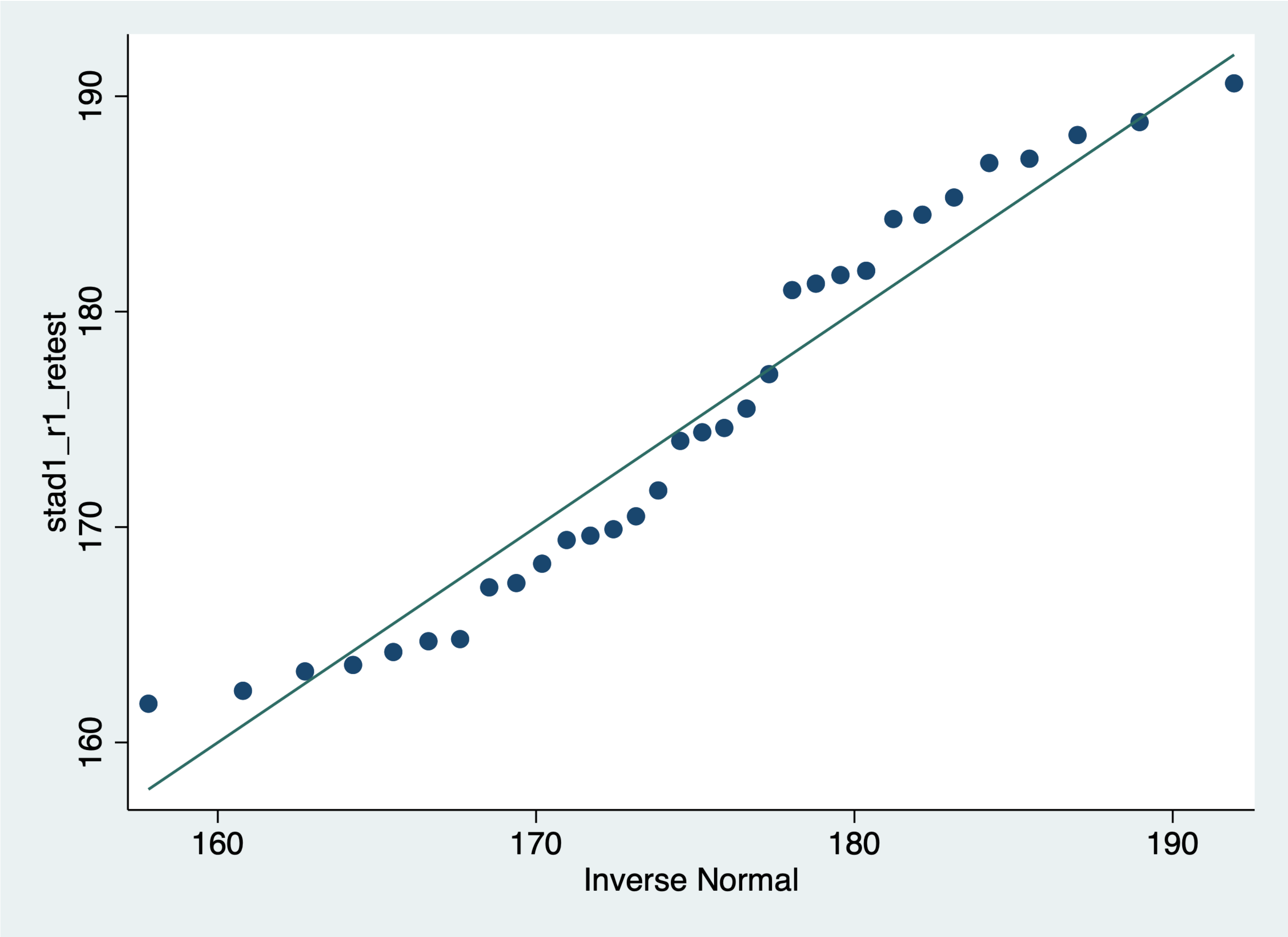

stad1_r1_retest
190
180
170
160
160
170
180
190
Inverse Normal

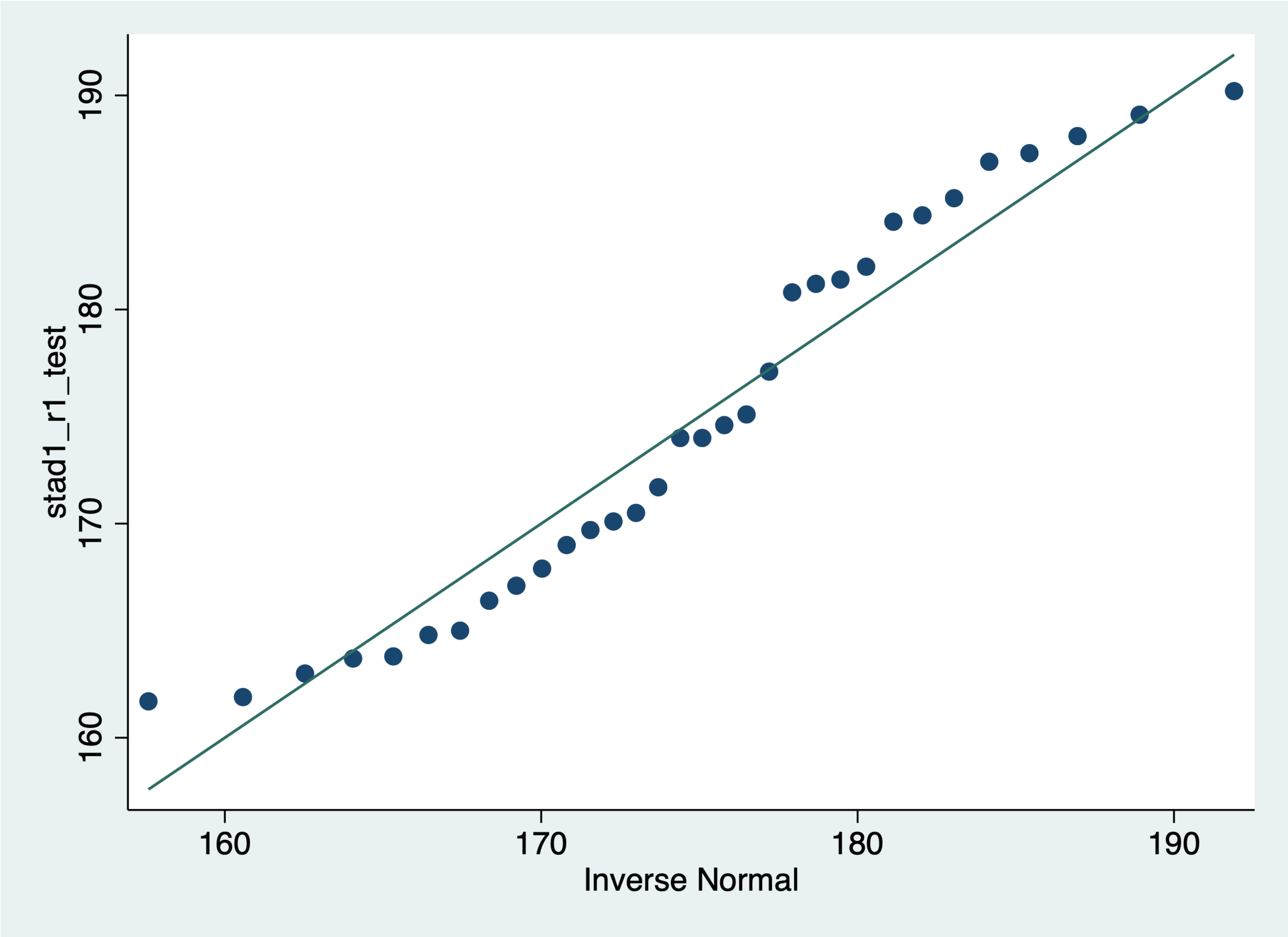

190
180
170
160
stad1_r1_test
160
170
180
190
Inverse Normal

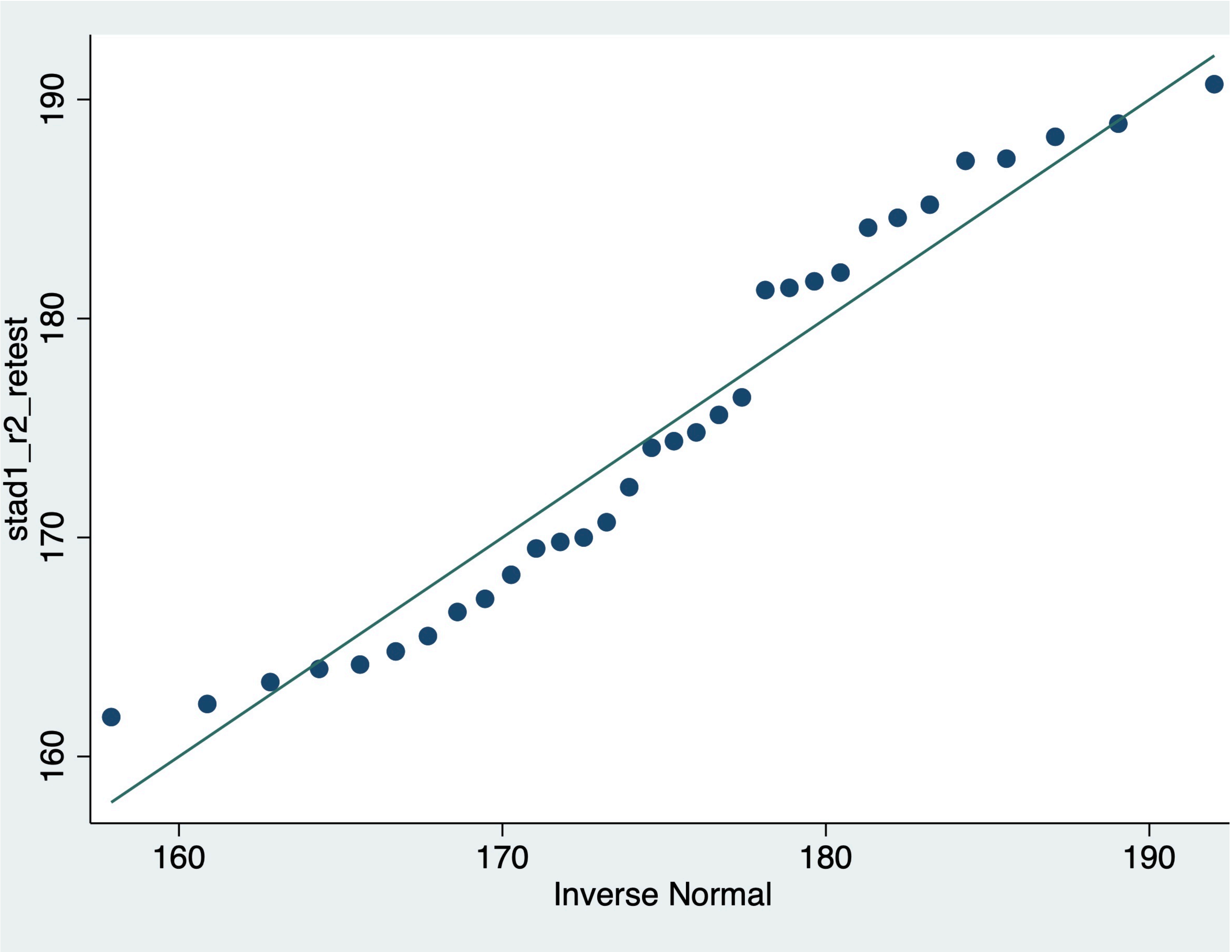

stad1_r2_retest
160
170
180
190
160
170
180
190
Inverse Normal

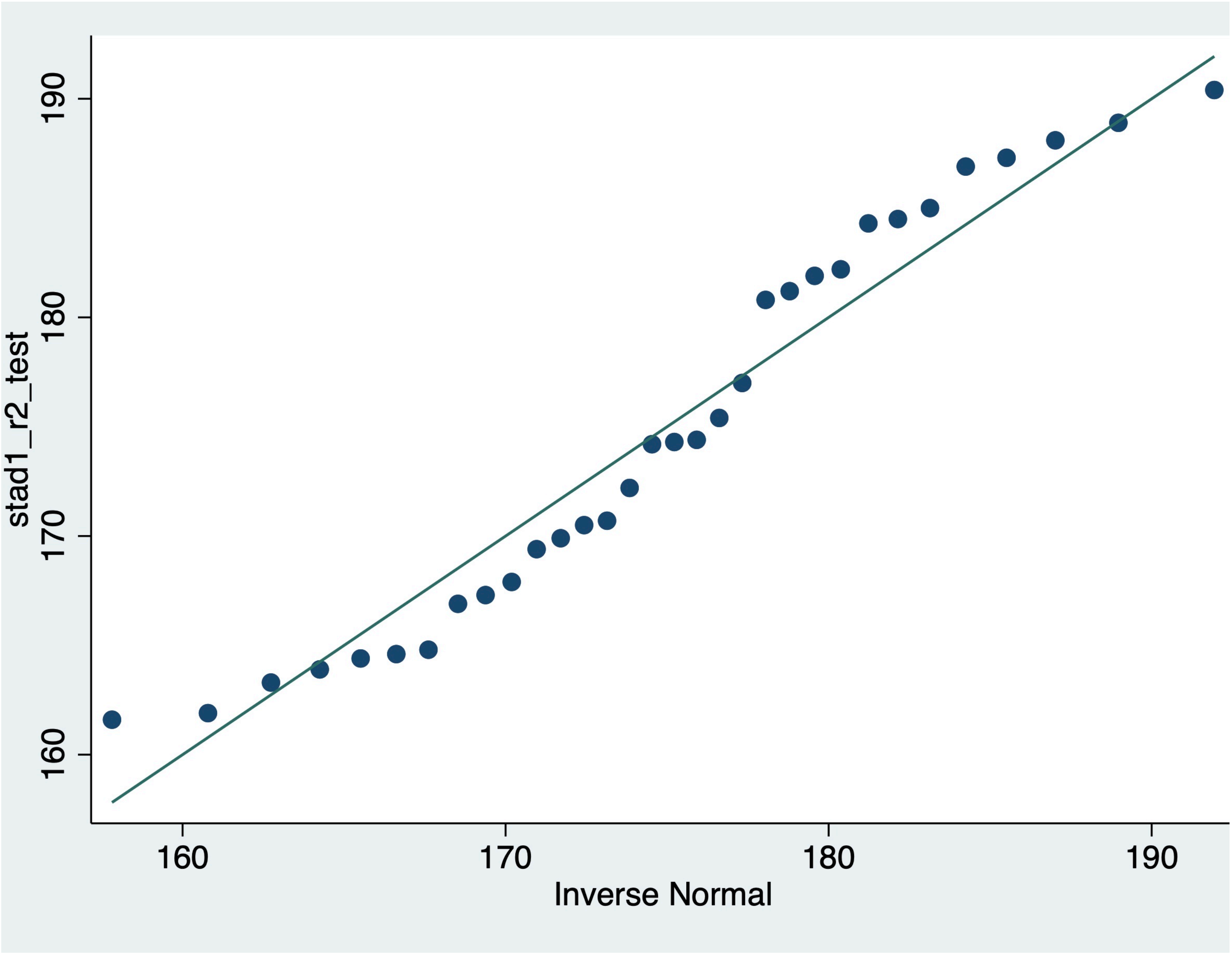

stad1_r2_test
190
180
170
160
160
170
180
190
Inverse Normal

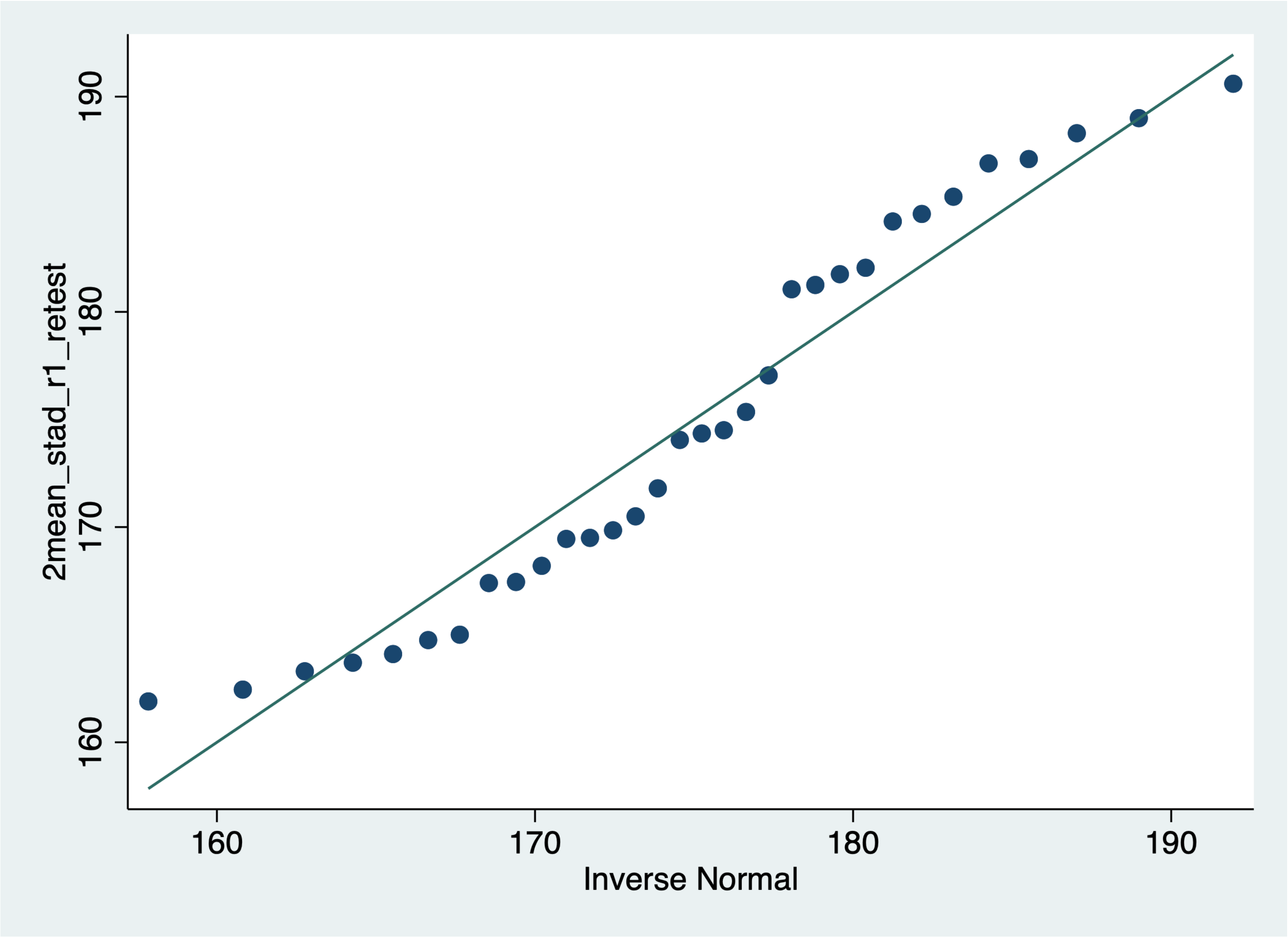

2mean_stad_r1_retest
160
170
180
190
160
170
180
190
Inverse Normal

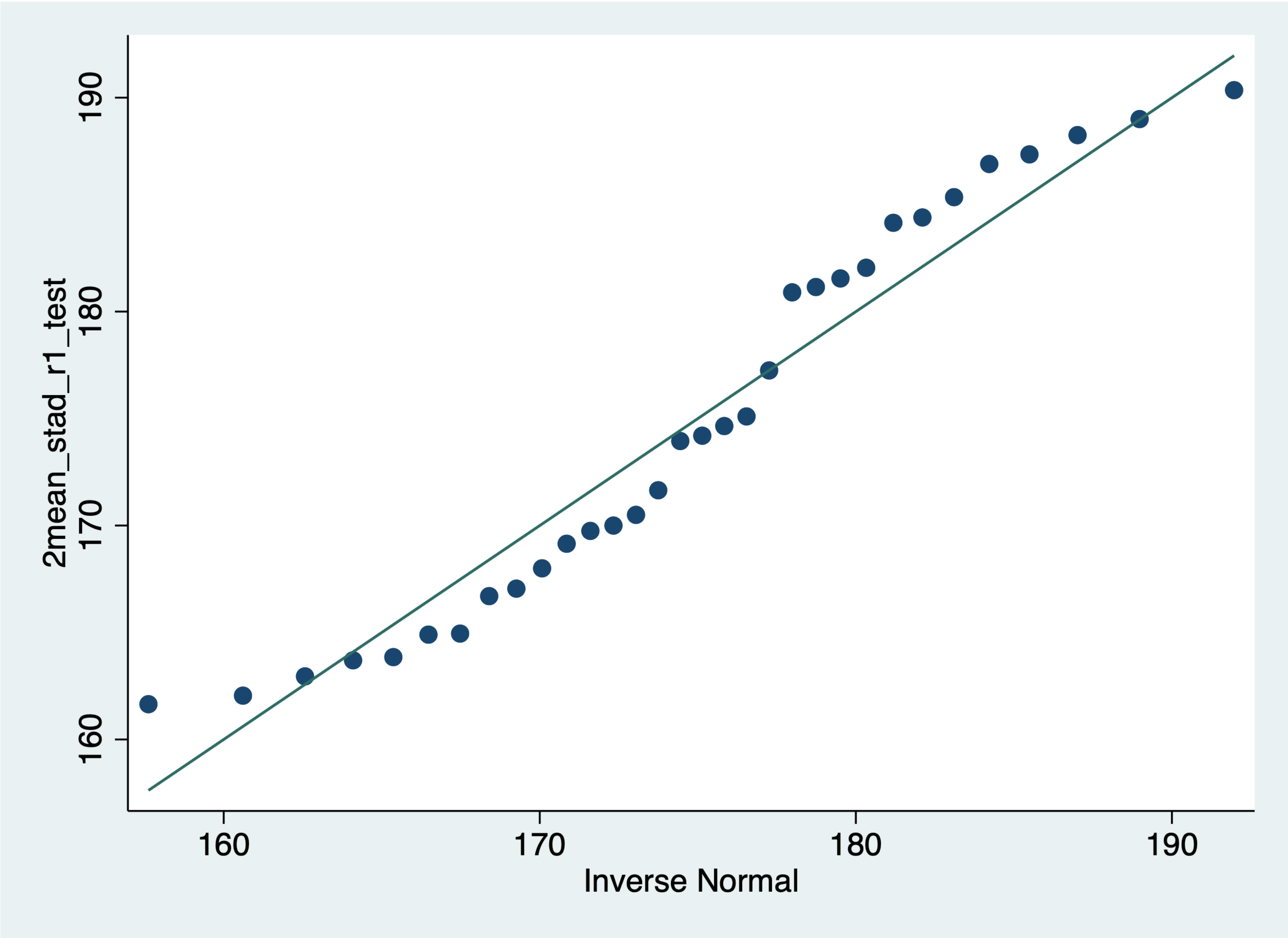

2mean_stad_r1_test
190
180
170
160
160
170
180
190
Inverse Normal

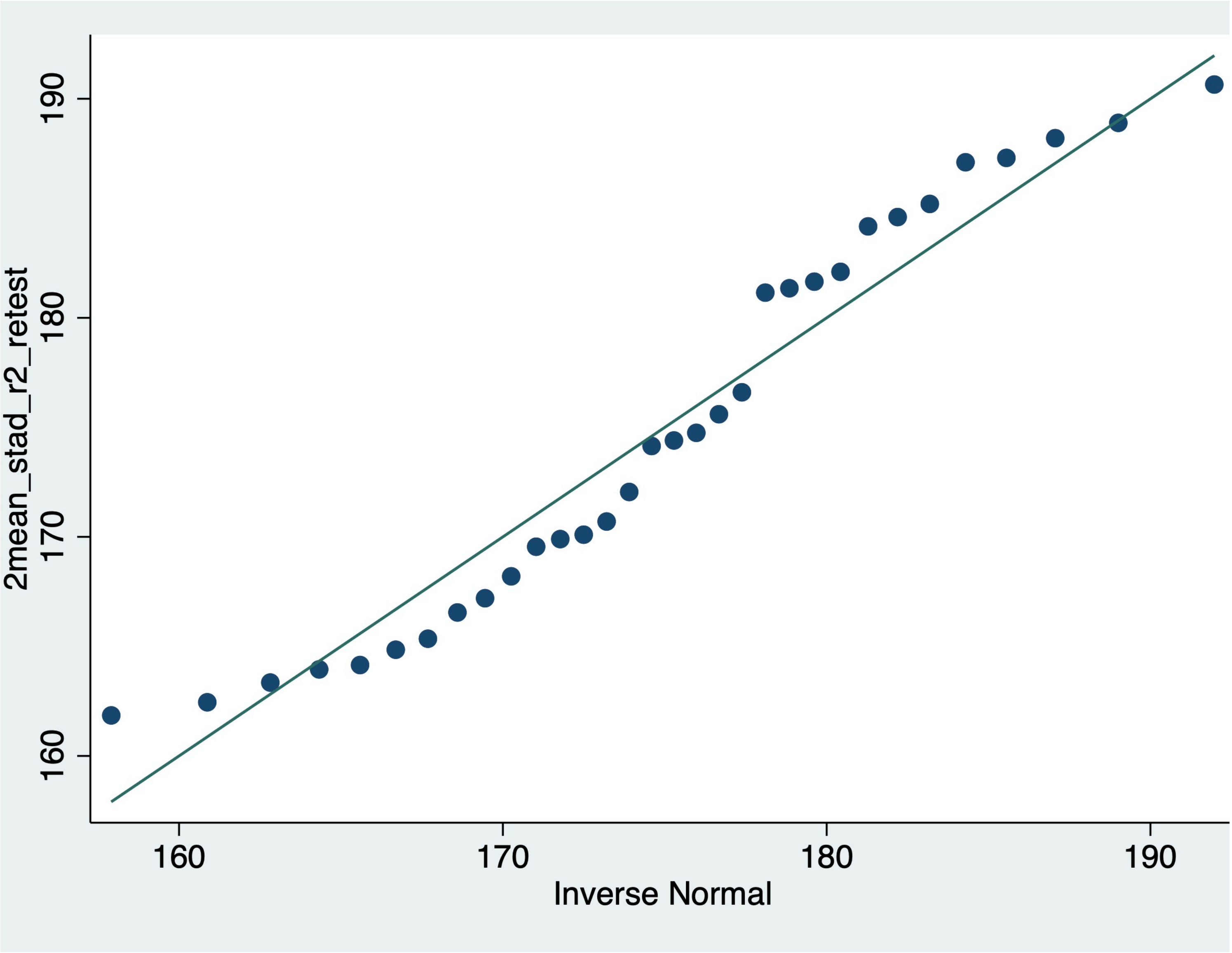
2mean_stad_r2_retest
160
170
180
190
160
170
180
190
Inverse Normal

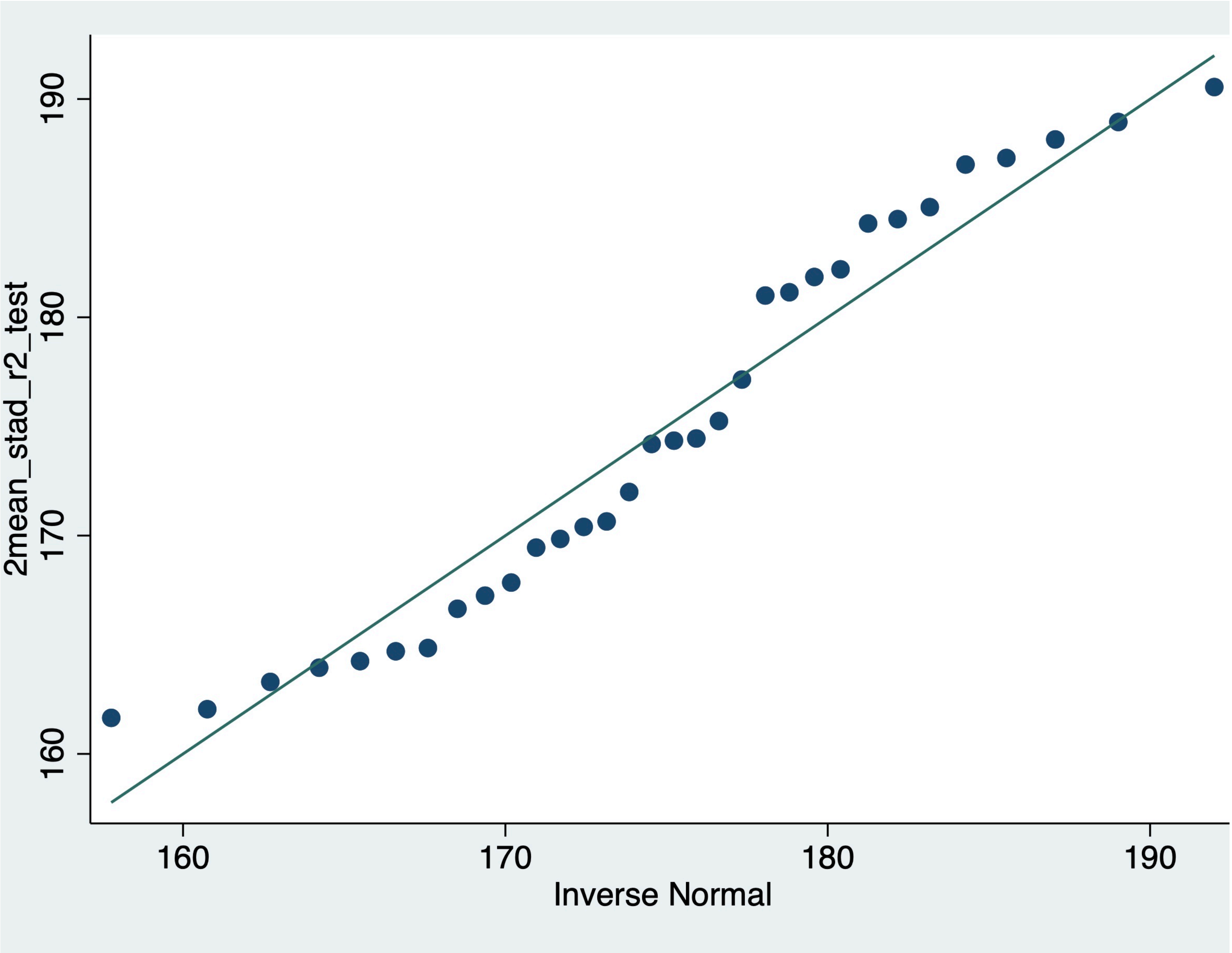
2mean_stad_r2_test
190
180
170
160
160
170
180
190
Inverse Normal

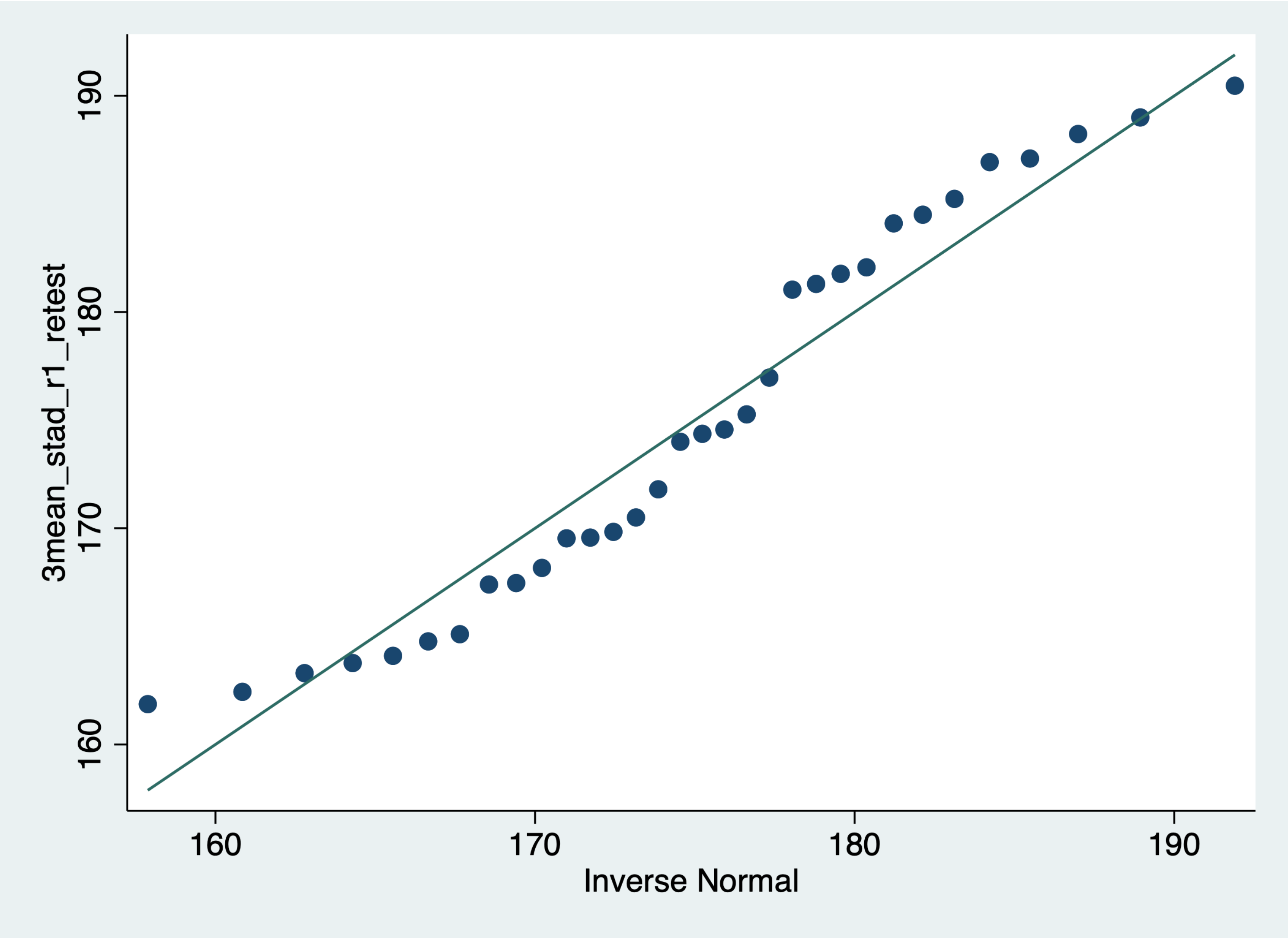
3mean_stad_r1_retest
160
170
180
190
160
170
180
190
Inverse Normal

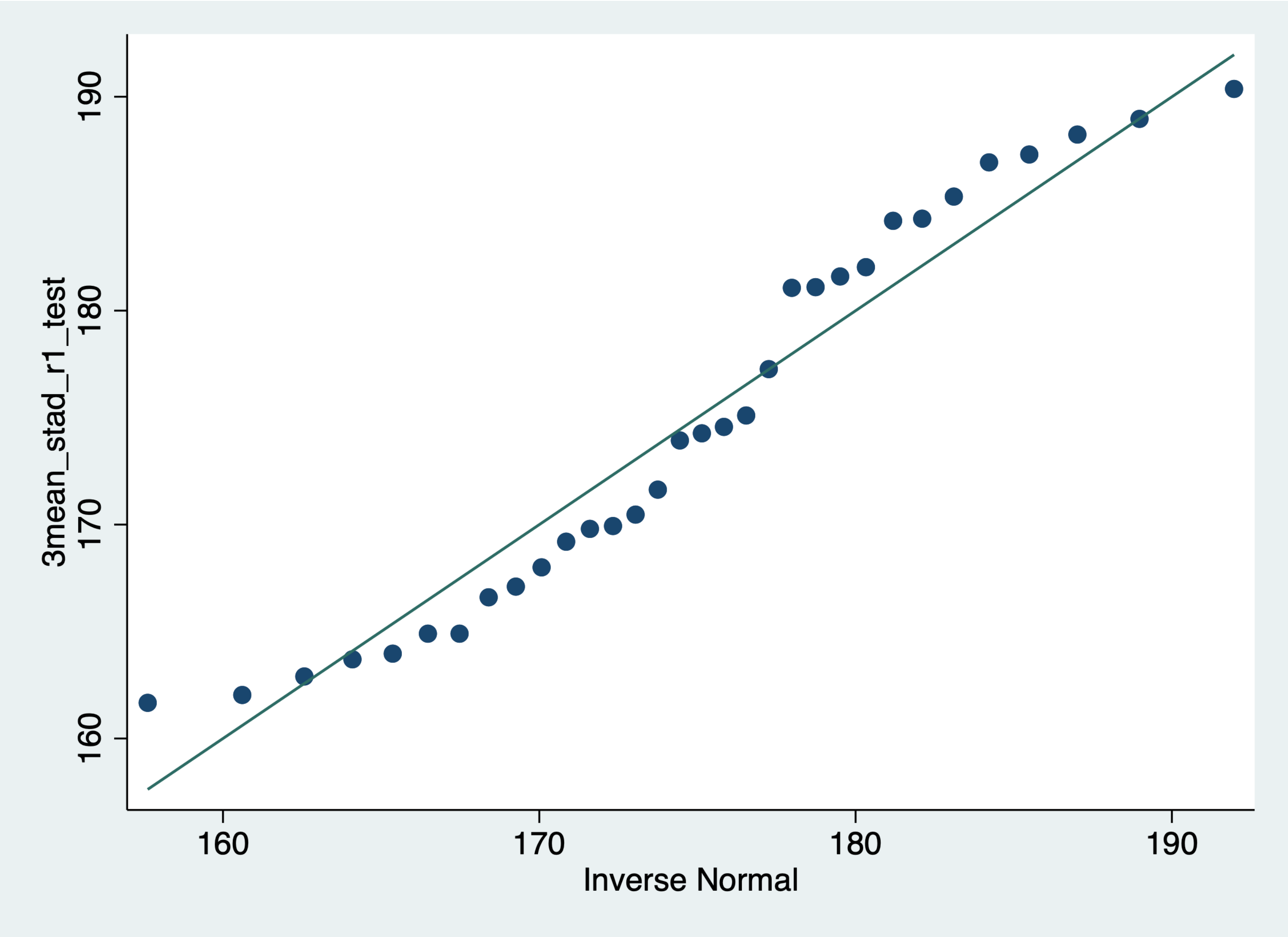

3mean_stad_r1_test
160
170
180
190
160
170
180
190
Inverse Normal

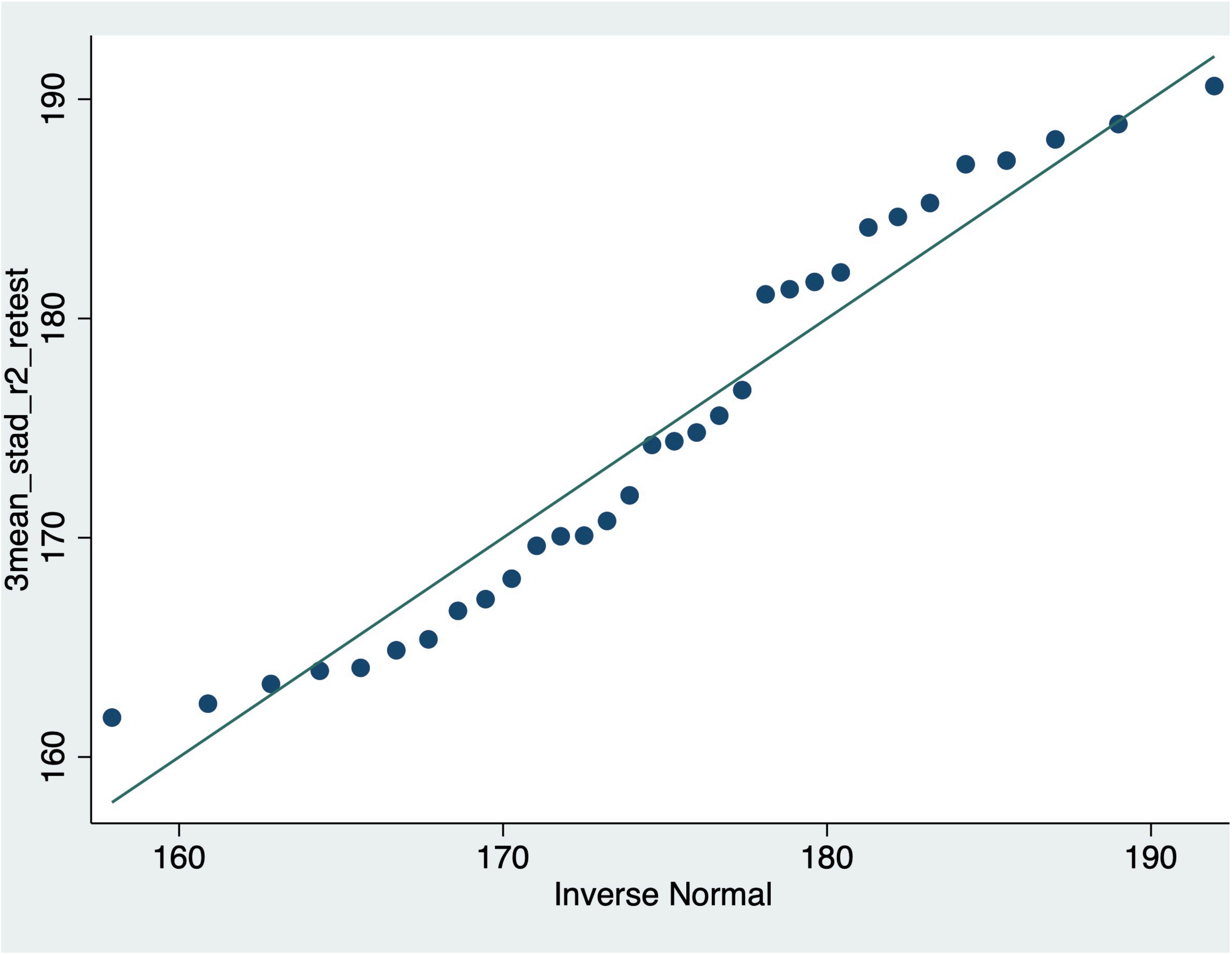

3mean_stad_r2_retest
160
170
180
190
160
170
180
190
Inverse Normal

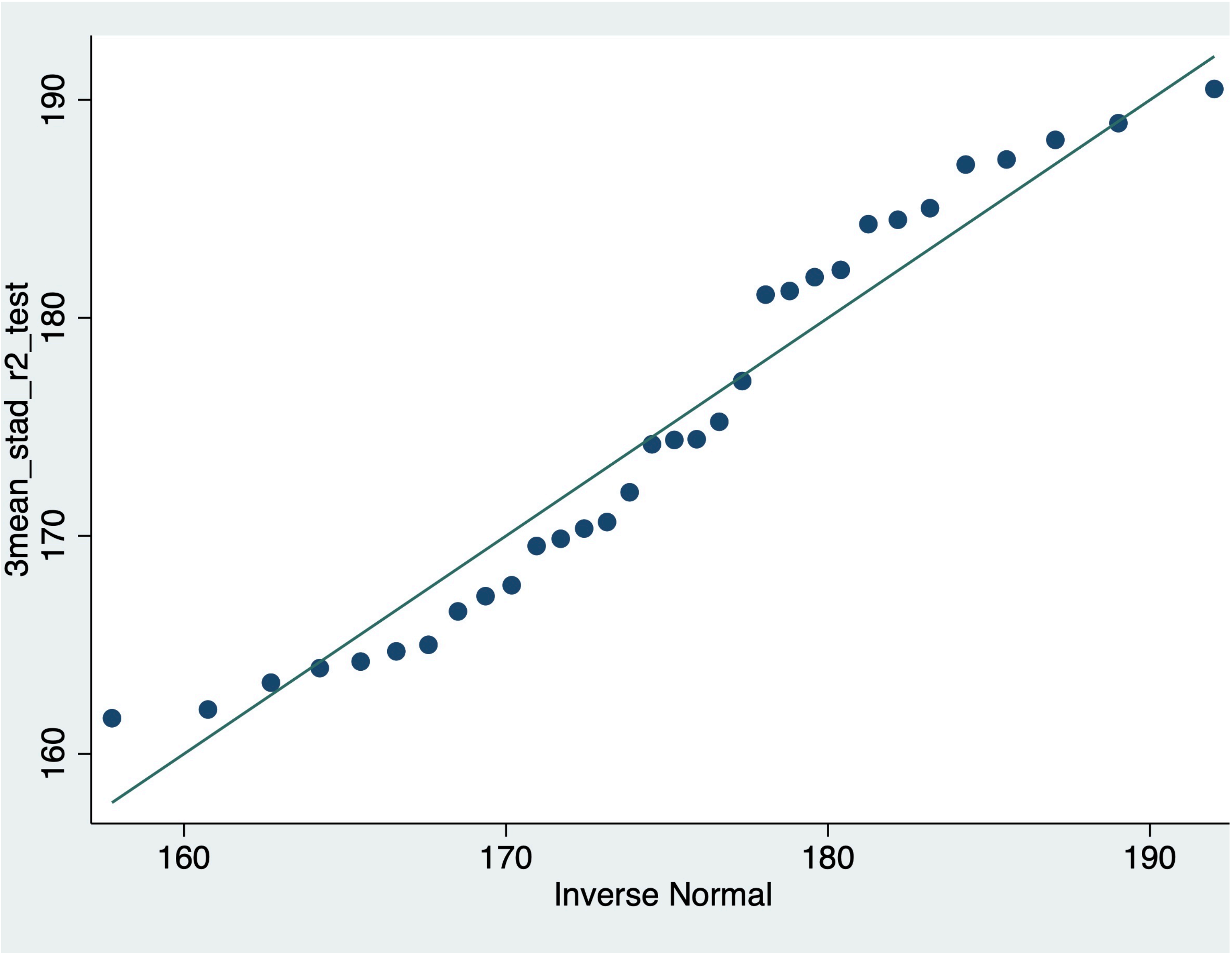

3mean_stad_r2_test
160
170
180
190
160
170
180
190
Inverse Normal

Histograms and QQ-plots used to check distribution for:

- Age
- Log_age

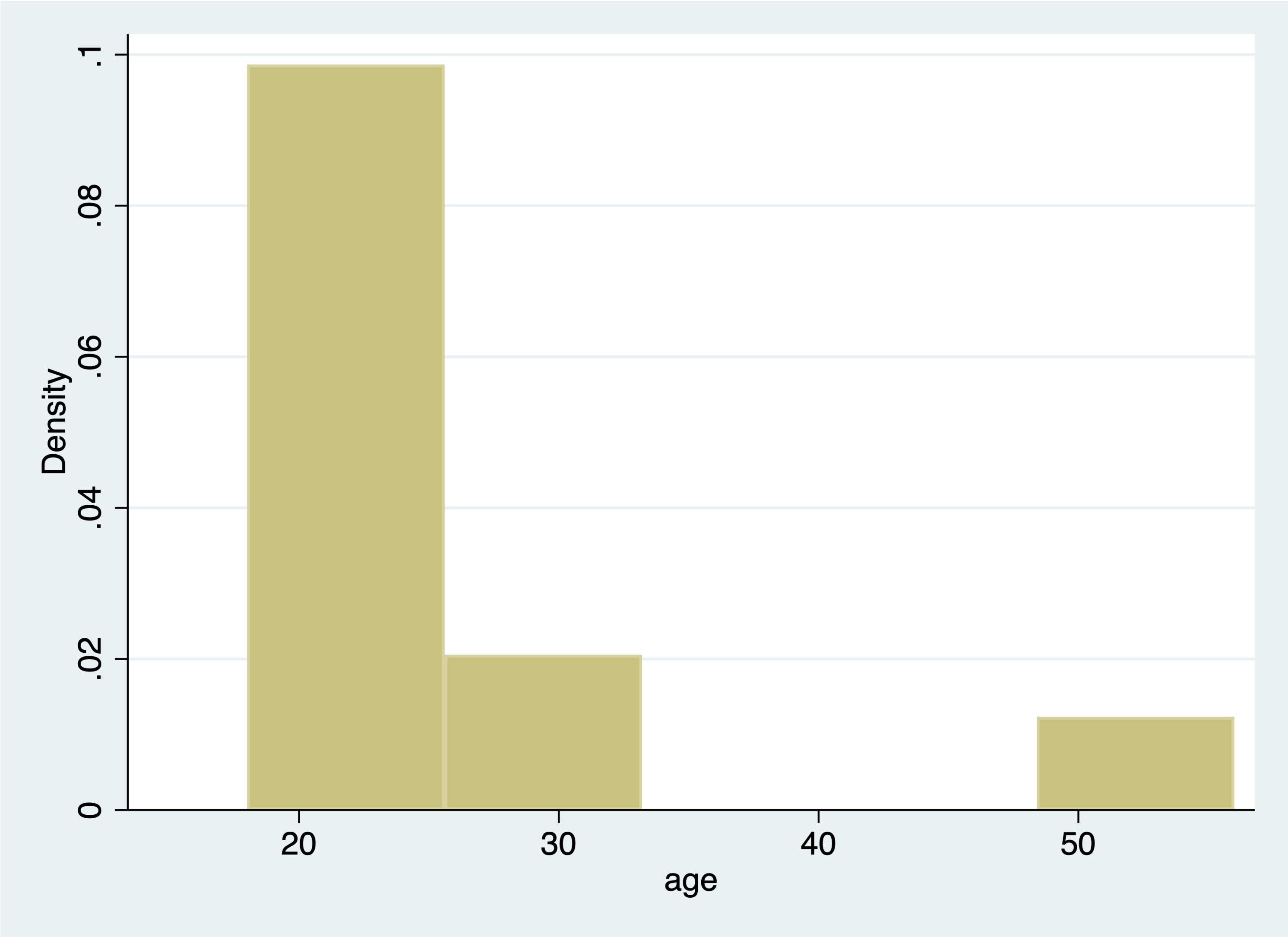

Density
0
.02
.04
.06
.08
.1
20
30
40
50
age

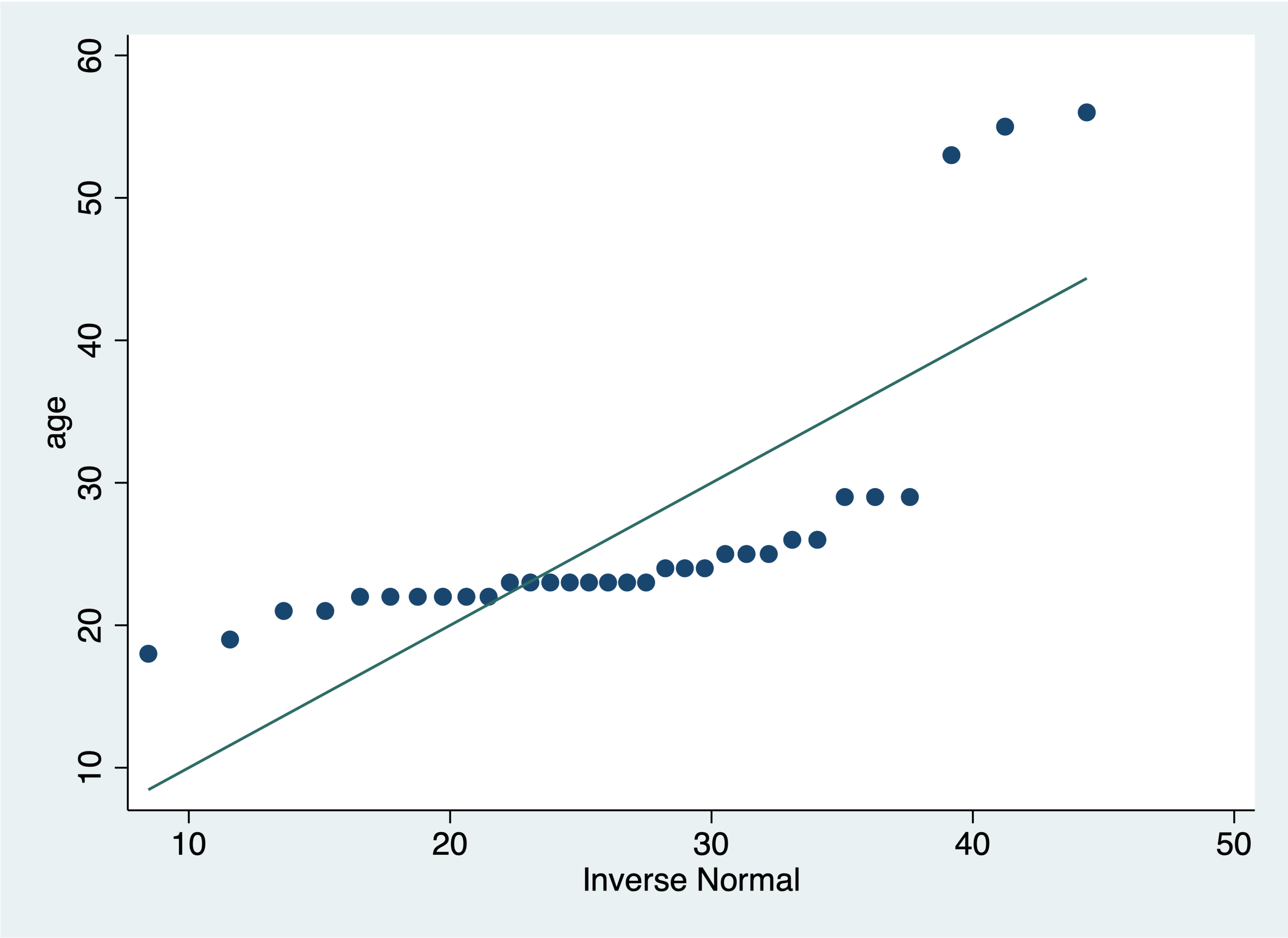

age
10
20
30
40
50
60
10
20
30
40
50
Inverse Normal

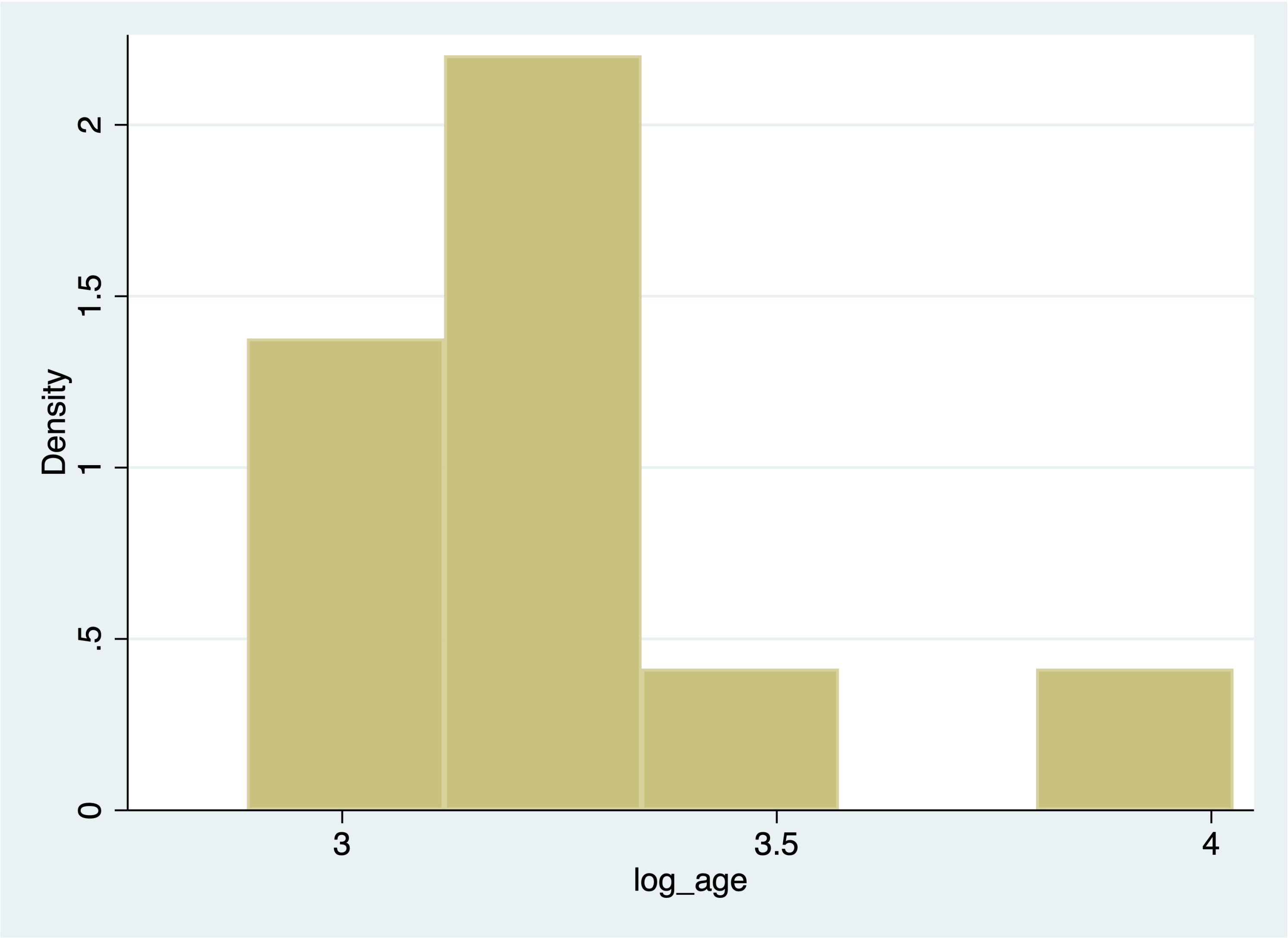

Density
0
.5
1
1.5
2
3
3.5
4
log_age

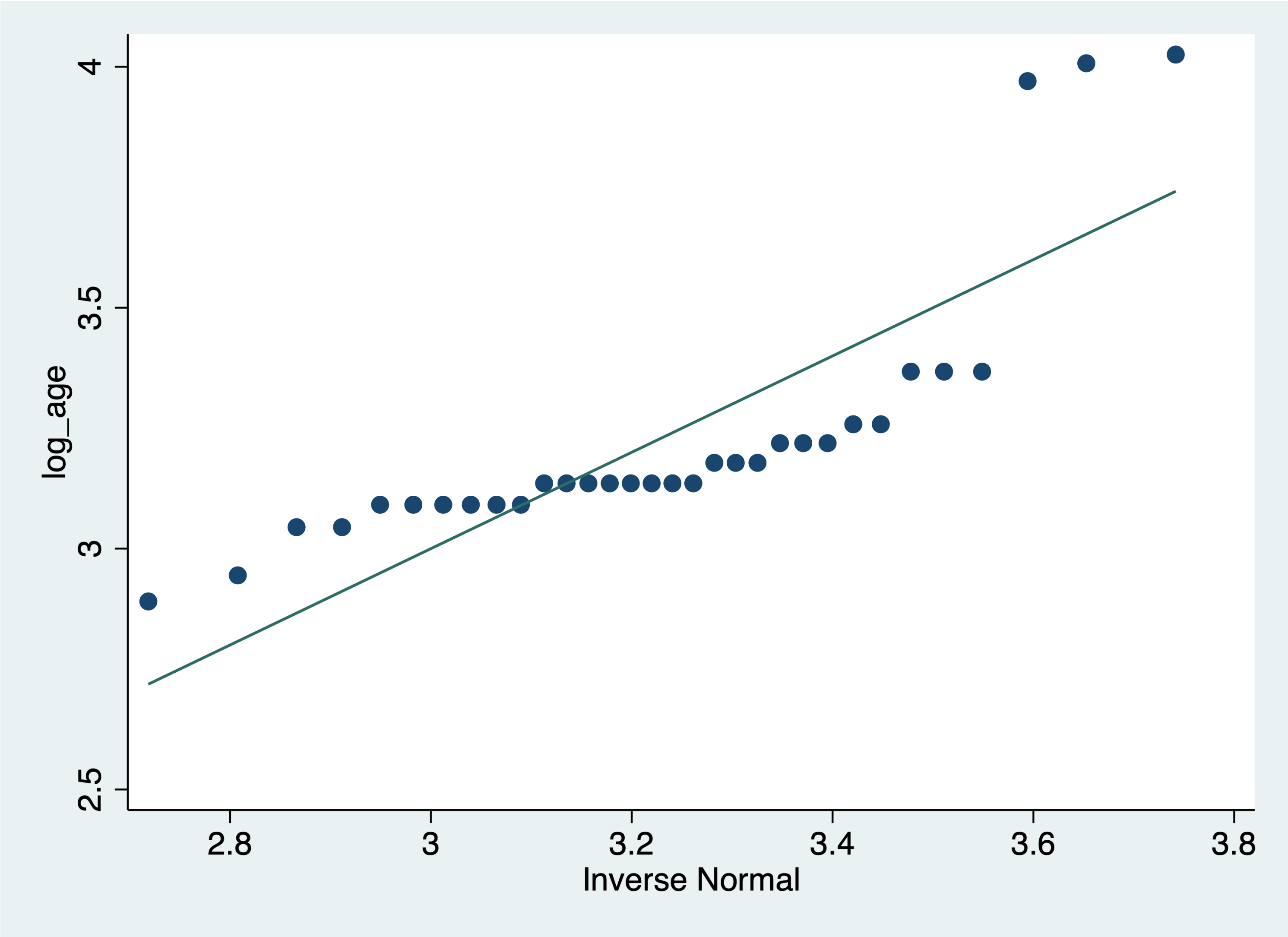

log_age
4
3.5
3
2.5
2.8
3
3.2
3.4
3.6
3.8
Inverse Normal
