# Supplementary material for: Reliability and agreement of a novel portable laser height metre
Source: PLoS One. 2020 Apr 8;15(4):e0231449. doi: 10.1371/journal.pone.0231449 (PMC7141692; doi:10.1371/journal.pone.0231449)
Supplement: S3 Appendix — (PDF) [file pone.0231449.s003.pdf]

Appendix 3

Bland-Altman plots of:

- Intrarater reliability
- Interrater reliability
- Method comparison

Legends:

- Blue line = Mean difference.
- Black lines = Upper and lower limits of agreement.
- Red lines = 95 % confidence intervals of estimates.

Intrarater reliability

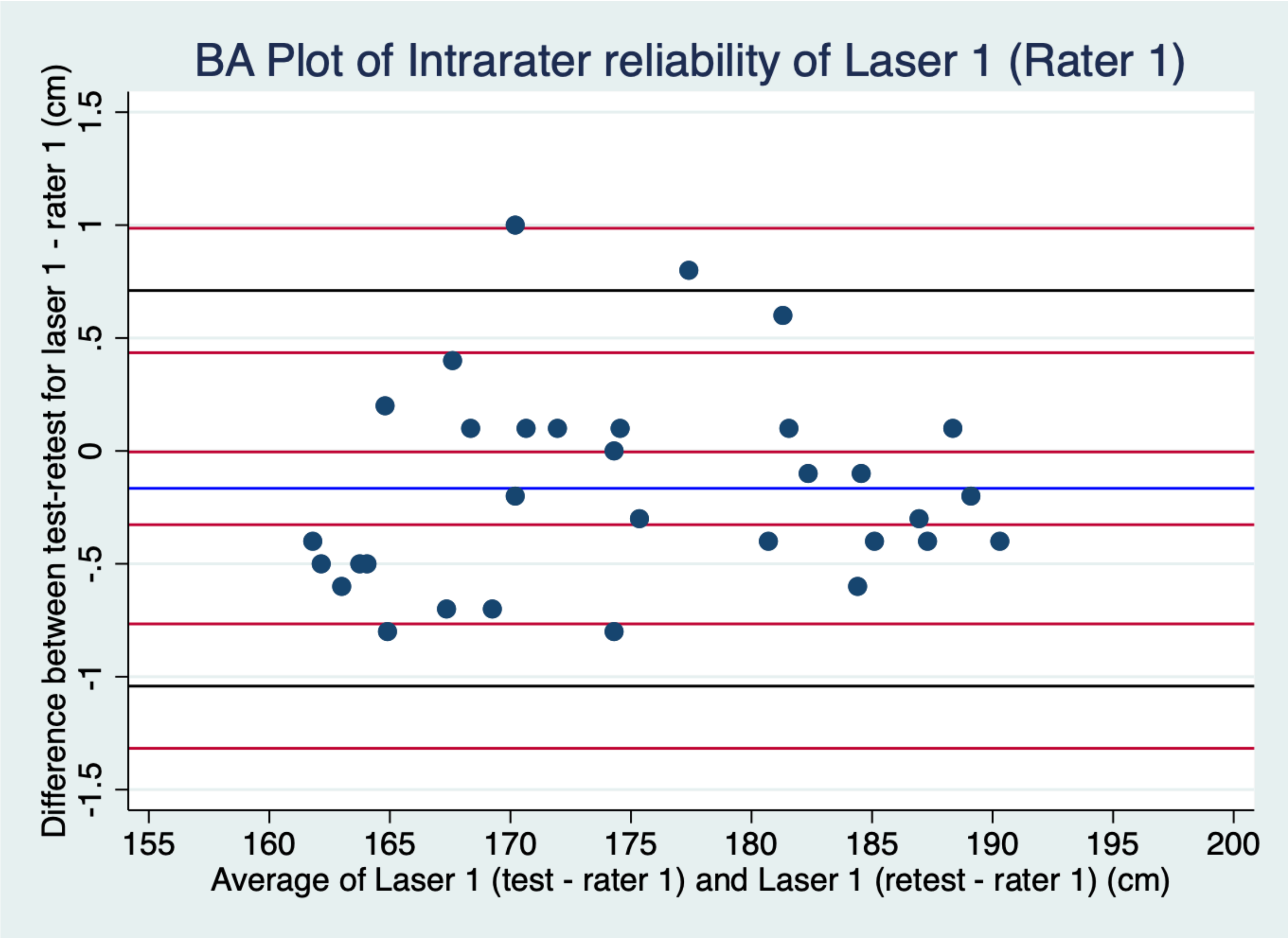
BA Plot of Intrarater reliability of Laser 1 (Rater 1)
Difference between test-retest for laser 1 - rater 1 (cm)
-1.5
-1
-.5
0
.5
1
1.5
155
160
165
170
175
180
185
190
195
200
Average of Laser 1 (test - rater 1) and Laser 1 (retest - rater 1) (cm)

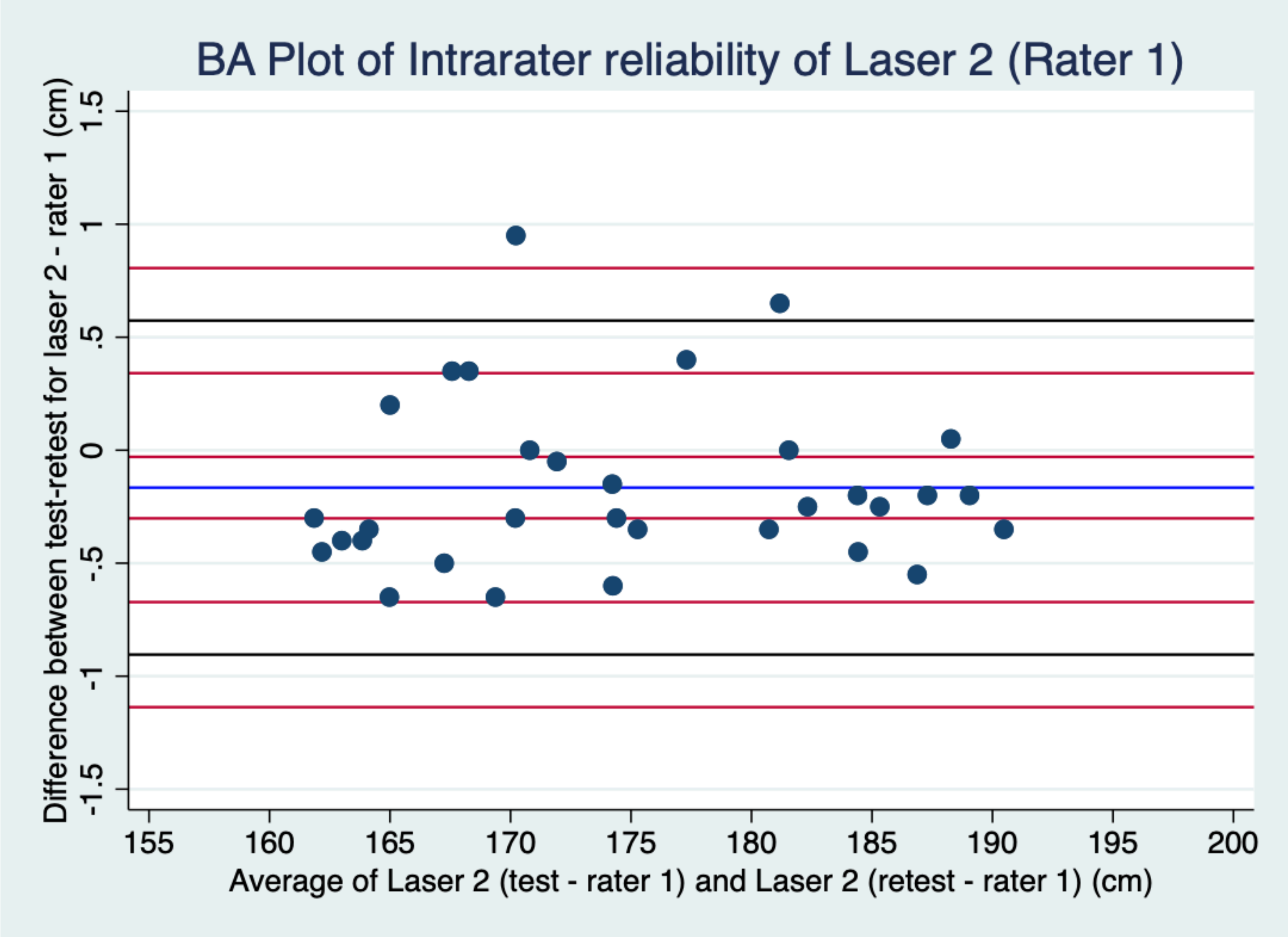

BA Plot of Intrarater reliability of Laser 2 (Rater 1)
Difference between test-retest for laser 2 - rater 1 (cm)
-1.5
-1
-.5
0
.5
1
1.5
155
160
165
170
175
180
185
190
195
200
Average of Laser 2 (test - rater 1) and Laser 2 (retest - rater 1) (cm)

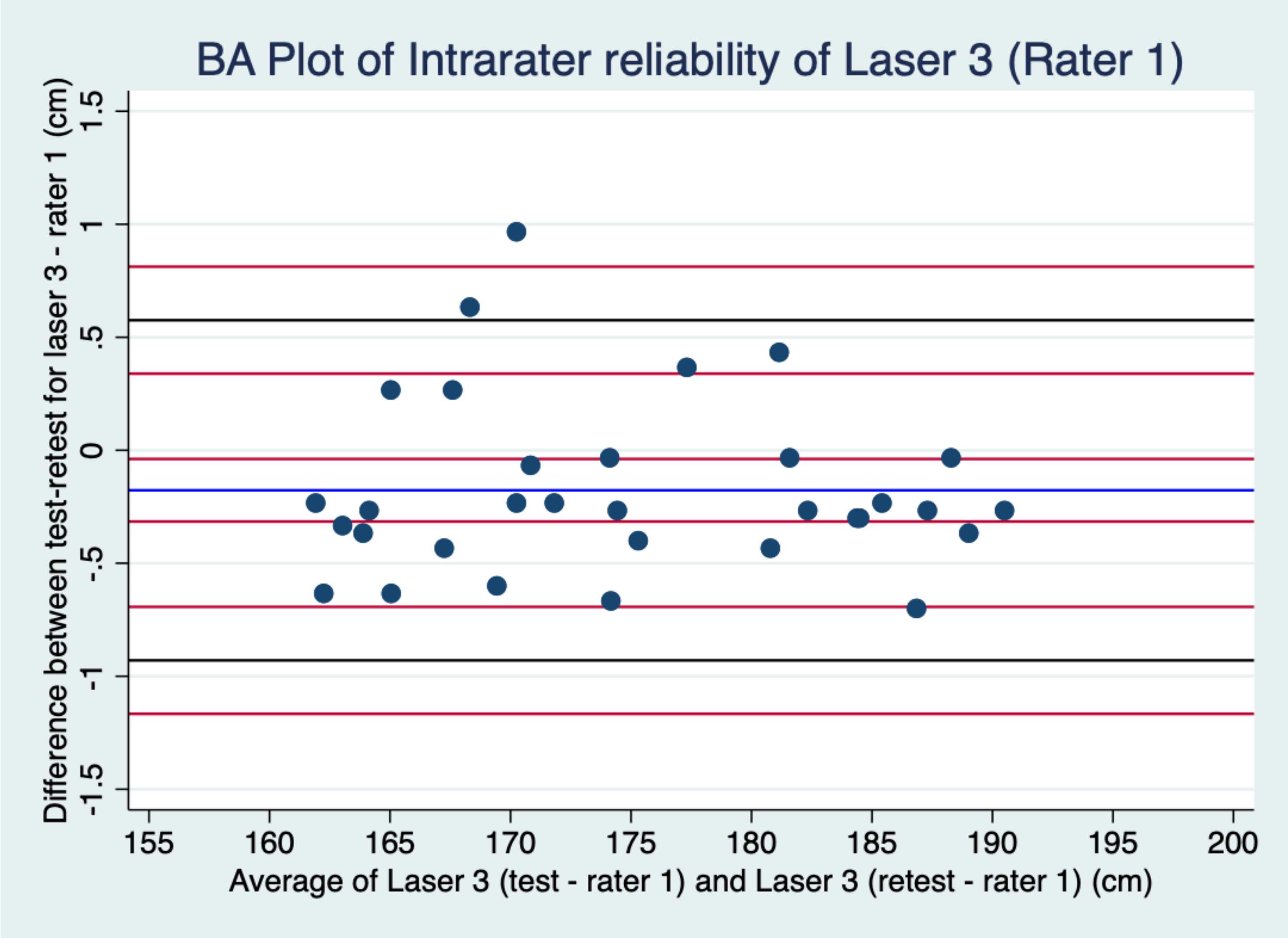
BA Plot of Intrarater reliability of Laser 3 (Rater 1)
Difference between test-retest for laser 3 - rater 1 (cm)
-1.5
-1
-.5
0
.5
1
1.5
155
160
165
170
175
180
185
190
195
200
Average of Laser 3 (test - rater 1) and Laser 3 (retest - rater 1) (cm)

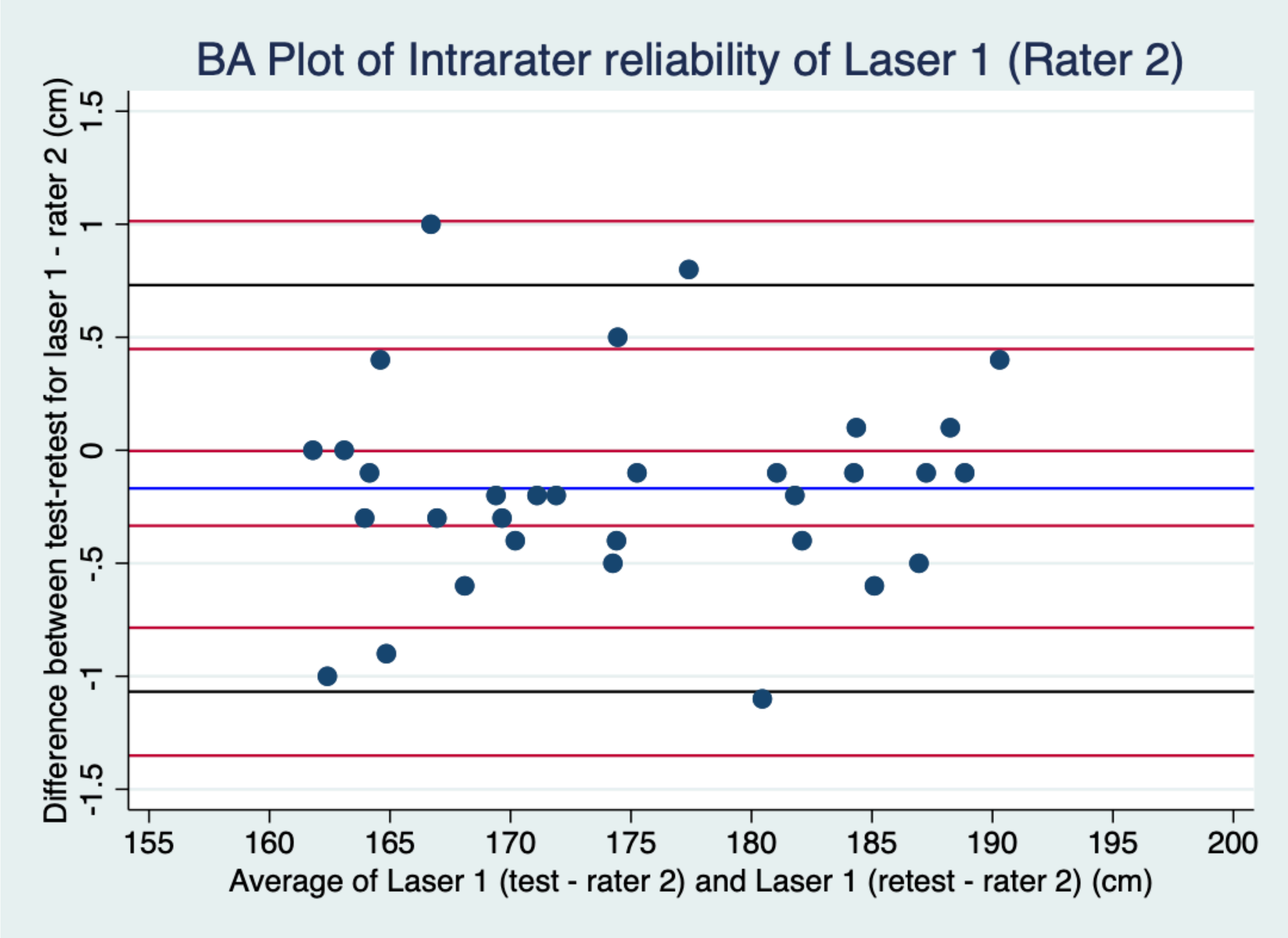
BA Plot of Intrarater reliability of Laser 1 (Rater 2)
Difference between test-retest for laser 1 - rater 2 (cm)
-1.5
-1
-.5
0
.5
1
1.5
155
160
165
170
175
180
185
190
195
200
Average of Laser 1 (test - rater 2) and Laser 1 (retest - rater 2) (cm)

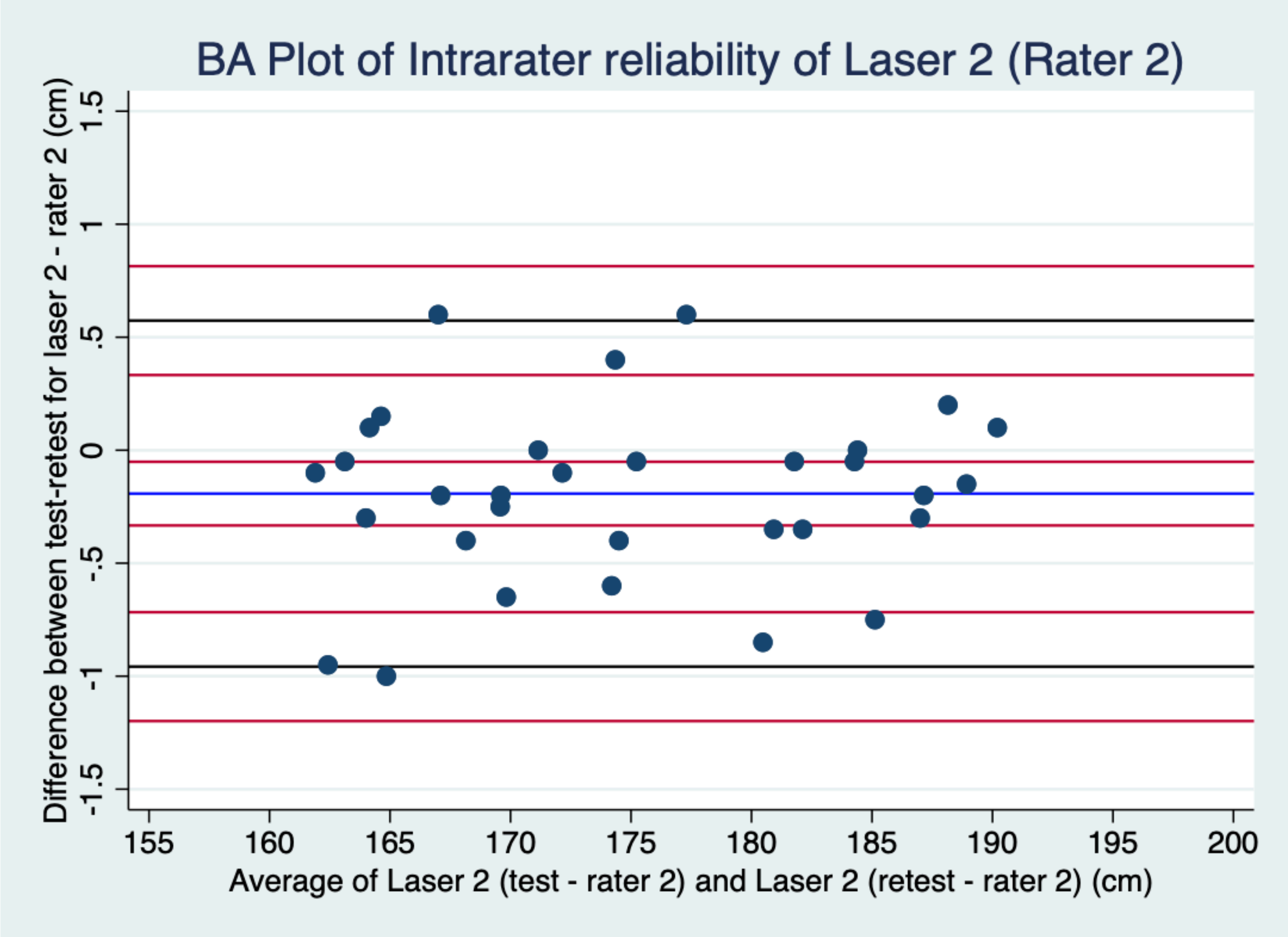
BA Plot of Intrarater reliability of Laser 2 (Rater 2)
Difference between test-retest for laser 2 - rater 2 (cm)
-1.5
-1
-.5
0
.5
1
1.5
155
160
165
170
175
180
185
190
195
200
Average of Laser 2 (test - rater 2) and Laser 2 (retest - rater 2) (cm)

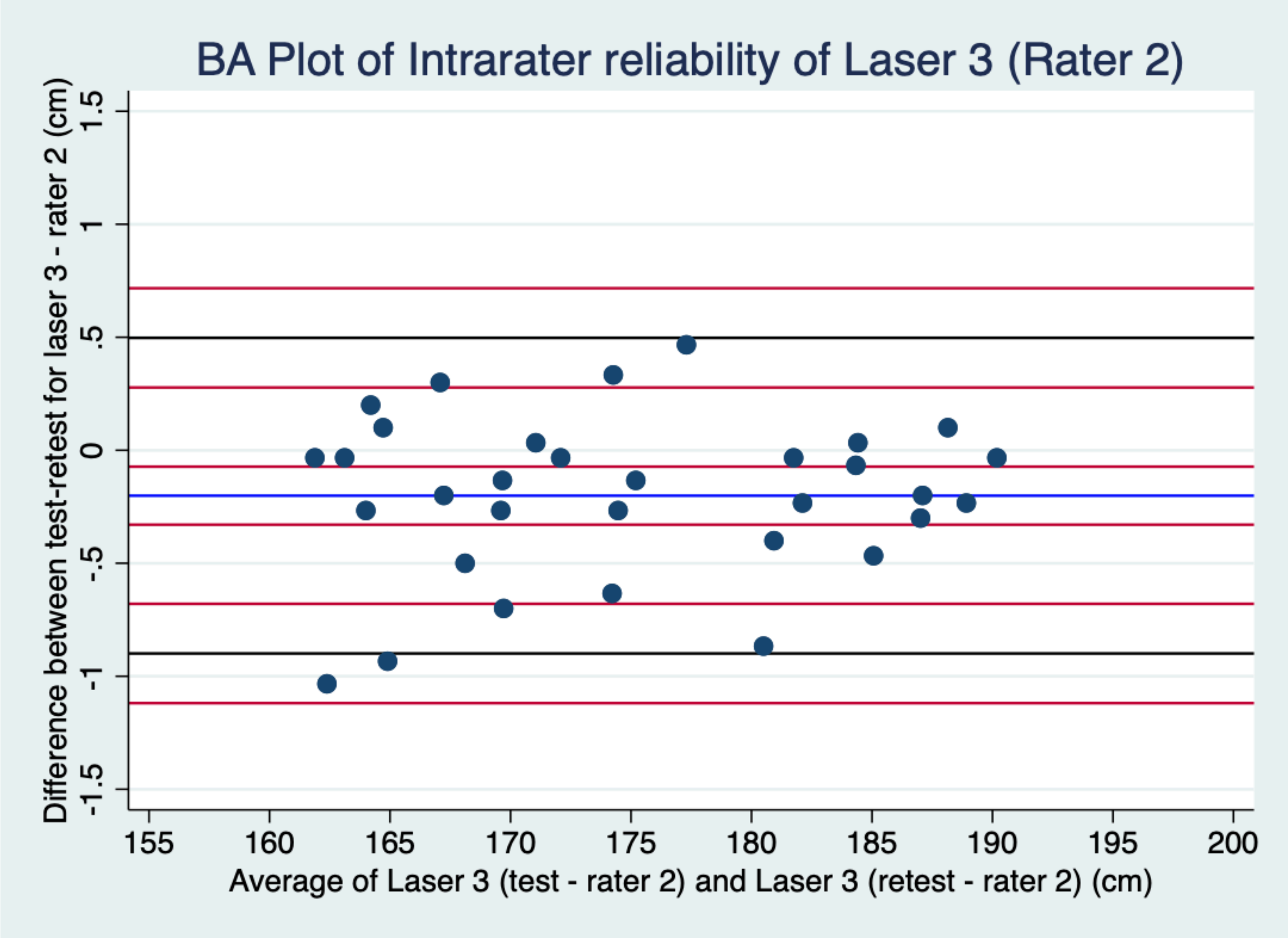

BA Plot of Intrarater reliability of Laser 3 (Rater 2)
Difference between test-retest for laser 3 - rater 2 (cm)
-1.5
-1
-.5
0
.5
1
1.5
155
160
165
170
175
180
185
190
195
200
Average of Laser 3 (test - rater 2) and Laser 3 (retest - rater 2) (cm)

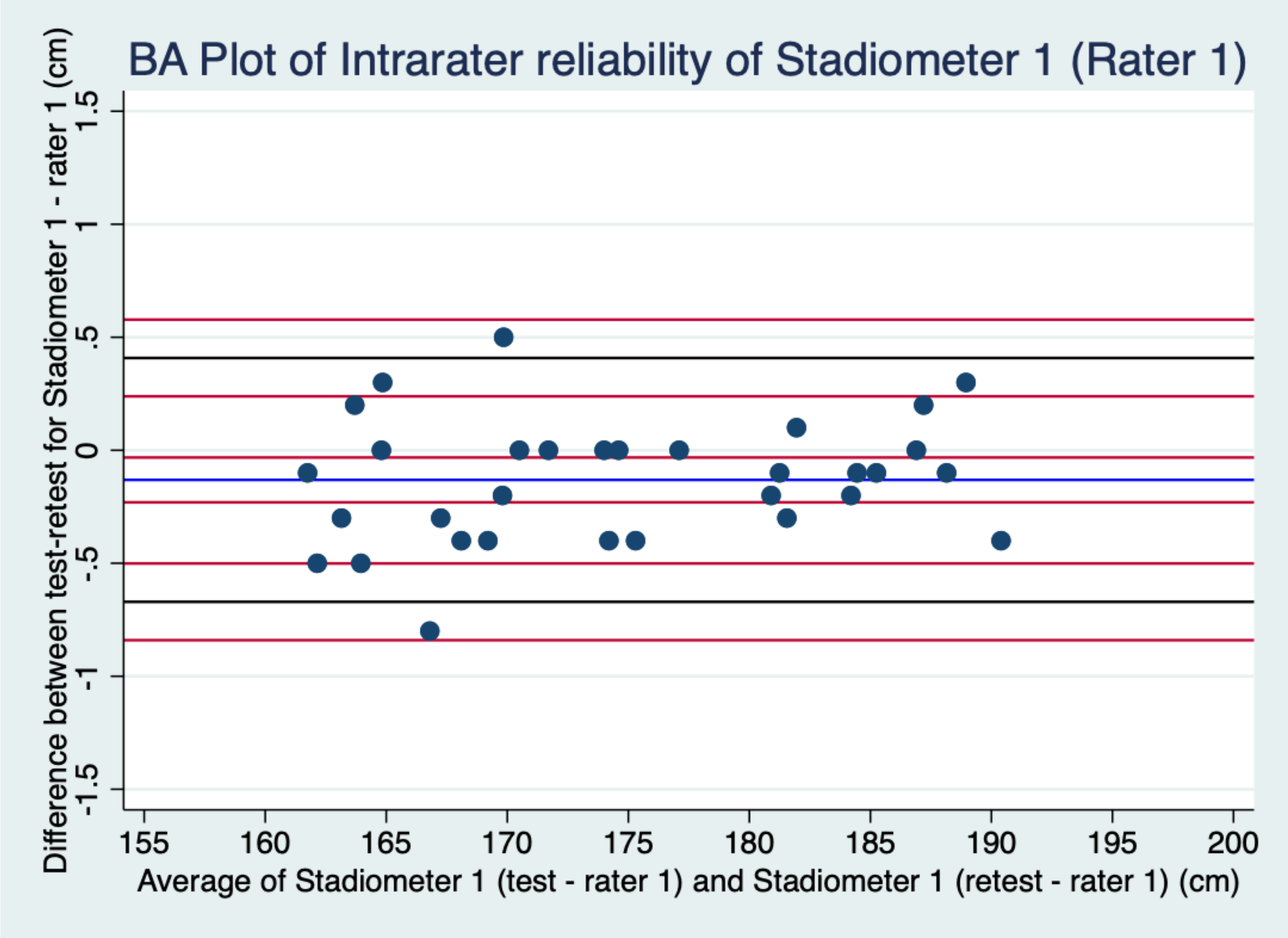

BA Plot of Intrarater reliability of Stadiometer 1 (Rater 1)
Difference between test-retest for Stadiometer 1 - rater 1 (cm)
-1.5
-1
-.5
0
.5
1
1.5
155
160
165
170
175
180
185
190
195
200
Average of Stadiometer 1 (test - rater 1) and Stadiometer 1 (retest - rater 1) (cm)

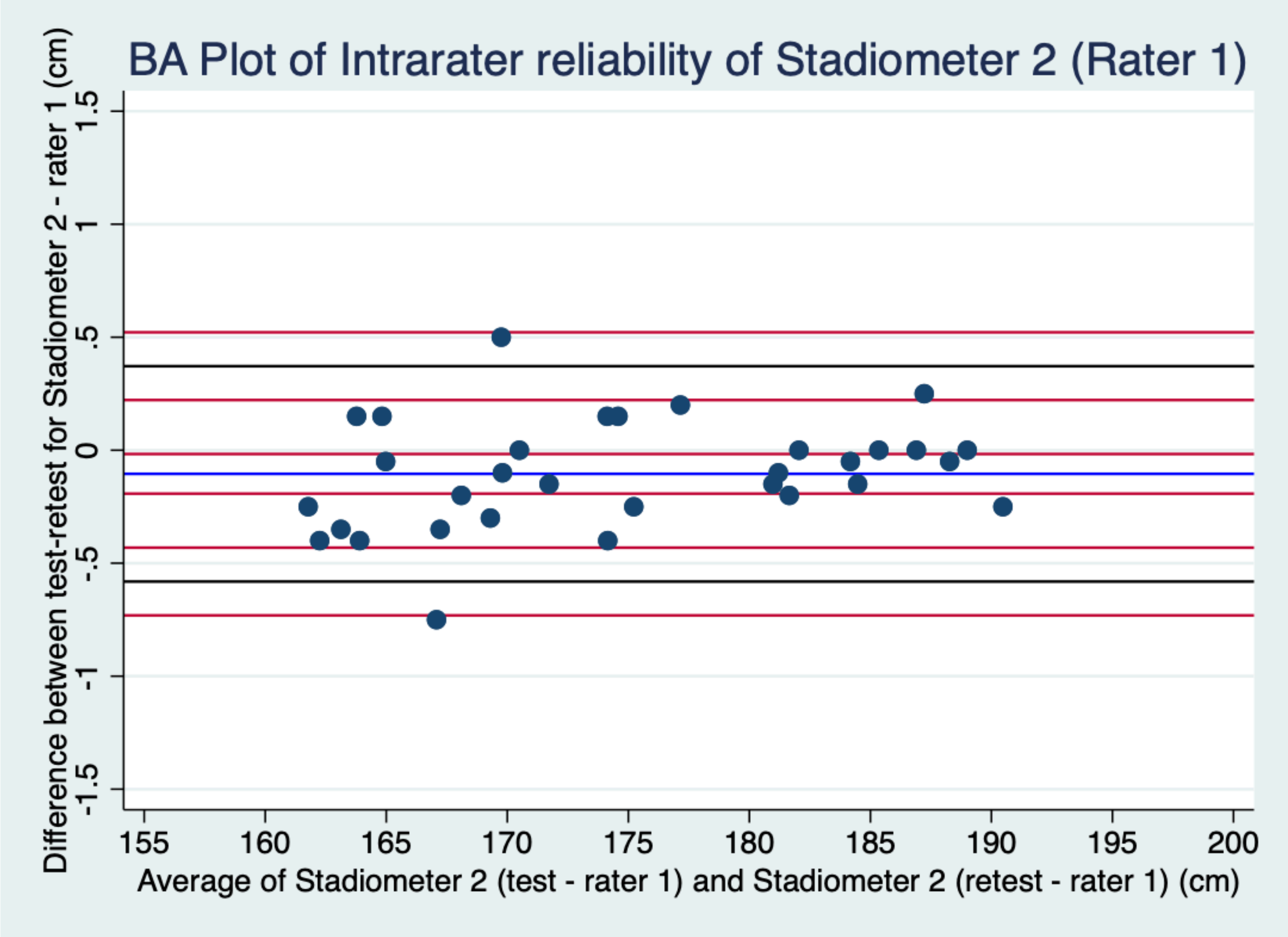
BA Plot of Intrarater reliability of Stadiometer 2 (Rater 1)
Difference between test-retest for Stadiometer 2 - rater 1 (cm)
Average of Stadiometer 2 (test - rater 1) and Stadiometer 2 (retest - rater 1) (cm)
-1.5
-1
-.5
0
.5
1
1.5
155
160
165
170
175
180
185
190
195
200

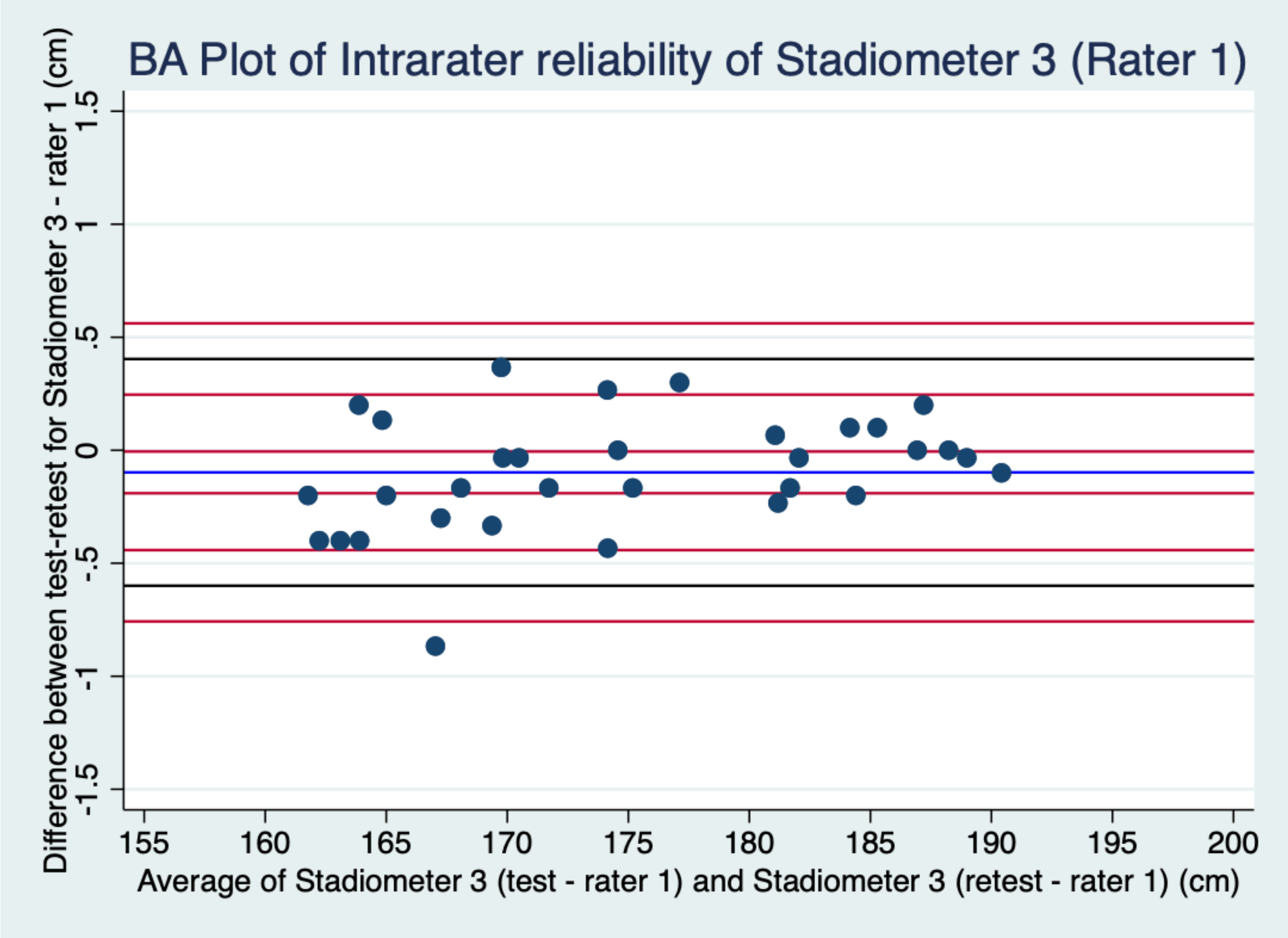
BA Plot of Intrarater reliability of Stadiometer 3 (Rater 1)
Difference between test-retest for Stadiometer 3 - rater 1 (cm)
-1.5
-1
-.5
0
.5
1
1.5
155
160
165
170
175
180
185
190
195
200
Average of Stadiometer 3 (test - rater 1) and Stadiometer 3 (retest - rater 1) (cm)

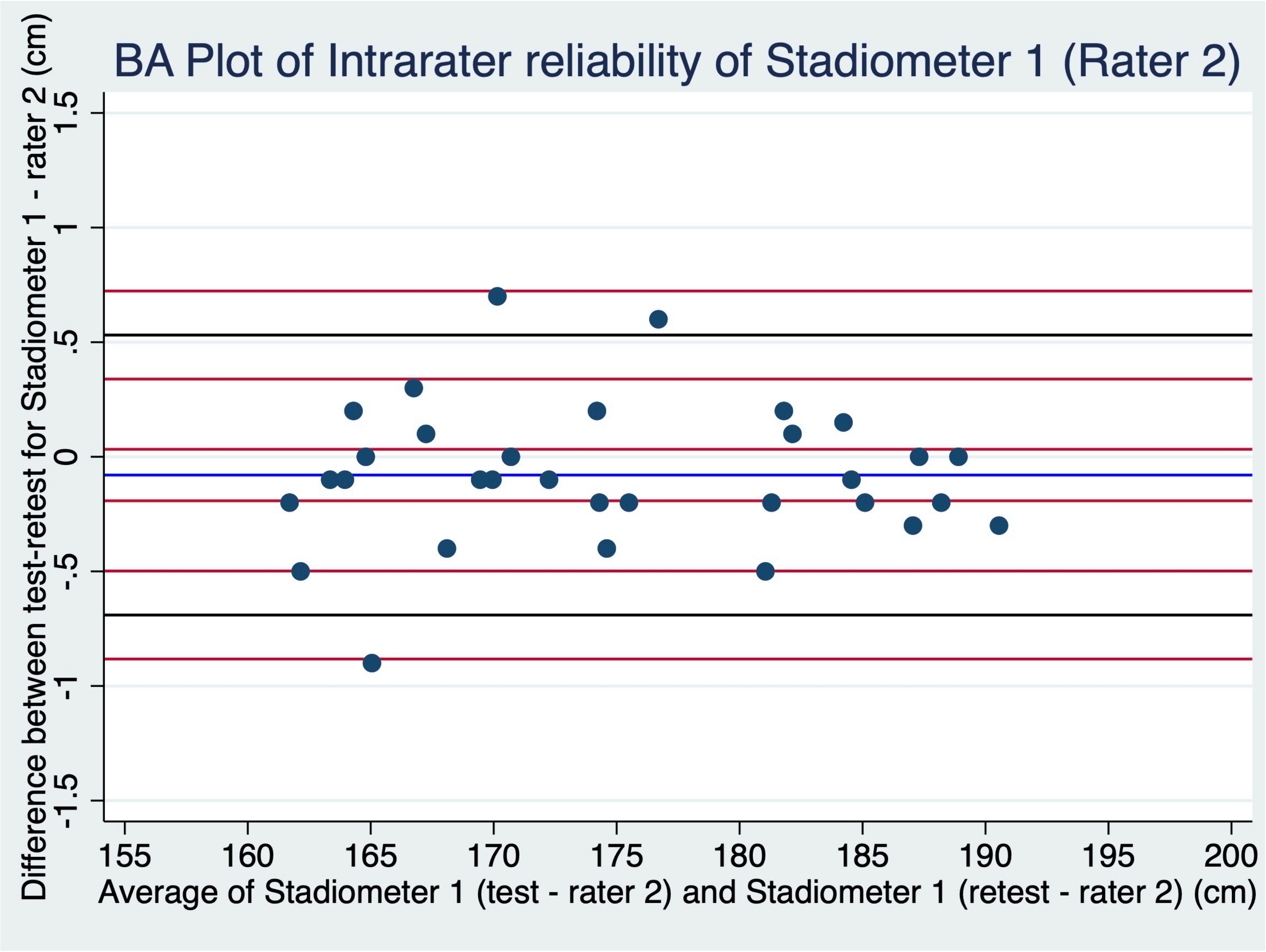
BA Plot of Intrarater reliability of Stadiometer 1 (Rater 2)
Difference between test-retest for Stadiometer 1 - rater 2 (cm)
1.5
1
.5
0
-.5
-1
-1.5
155
160
165
170
175
180
185
190
195
200
Average of Stadiometer 1 (test - rater 2) and Stadiometer 1 (retest - rater 2) (cm)

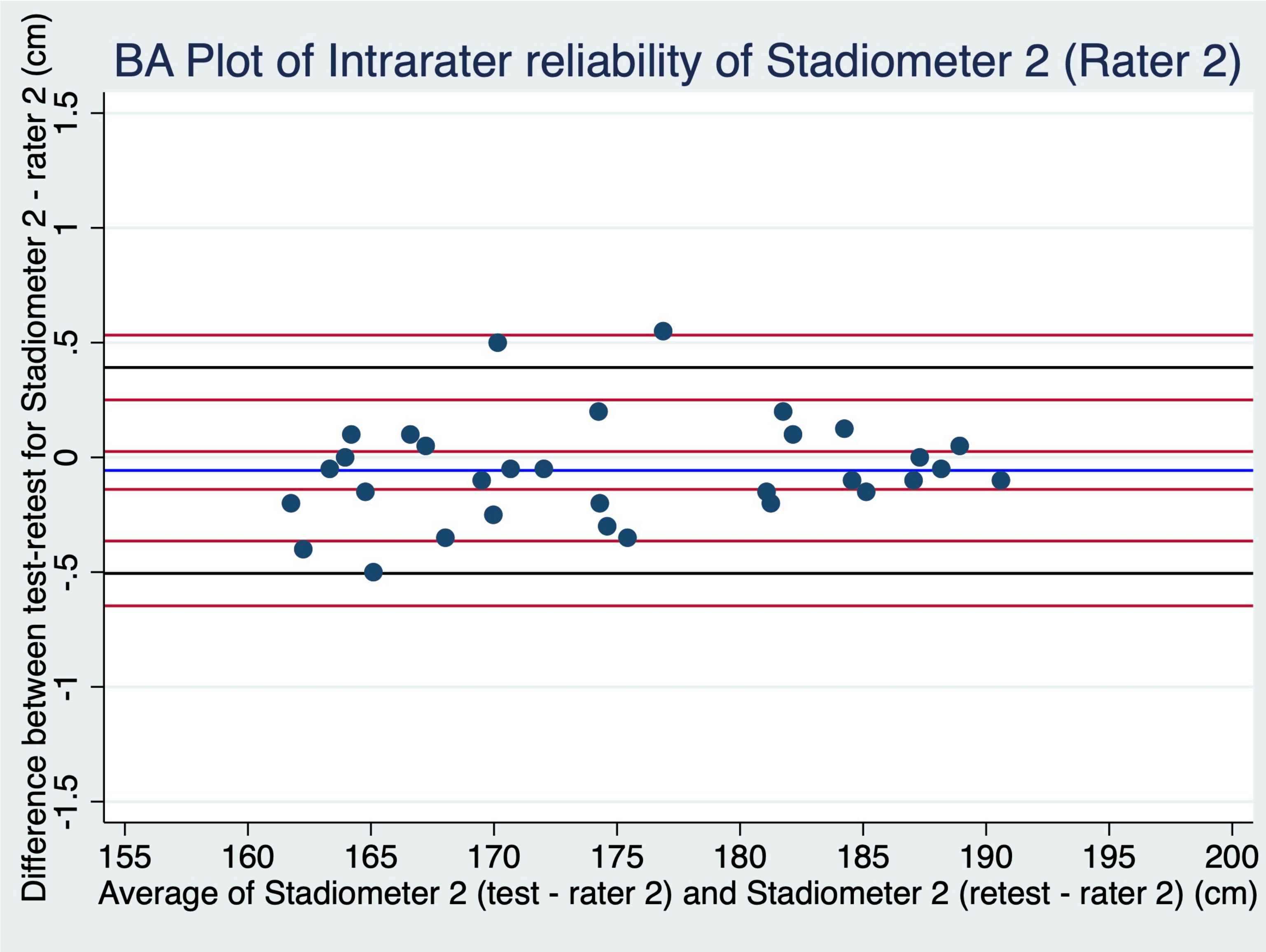

BA Plot of Intrarater reliability of Stadiometer 2 (Rater 2)
Difference between test-retest for Stadiometer 2 - rater 2 (cm)
-1.5
-1
-.5
0
.5
1
1.5
155
160
165
170
175
180
185
190
195
200
Average of Stadiometer 2 (test - rater 2) and Stadiometer 2 (retest - rater 2) (cm)

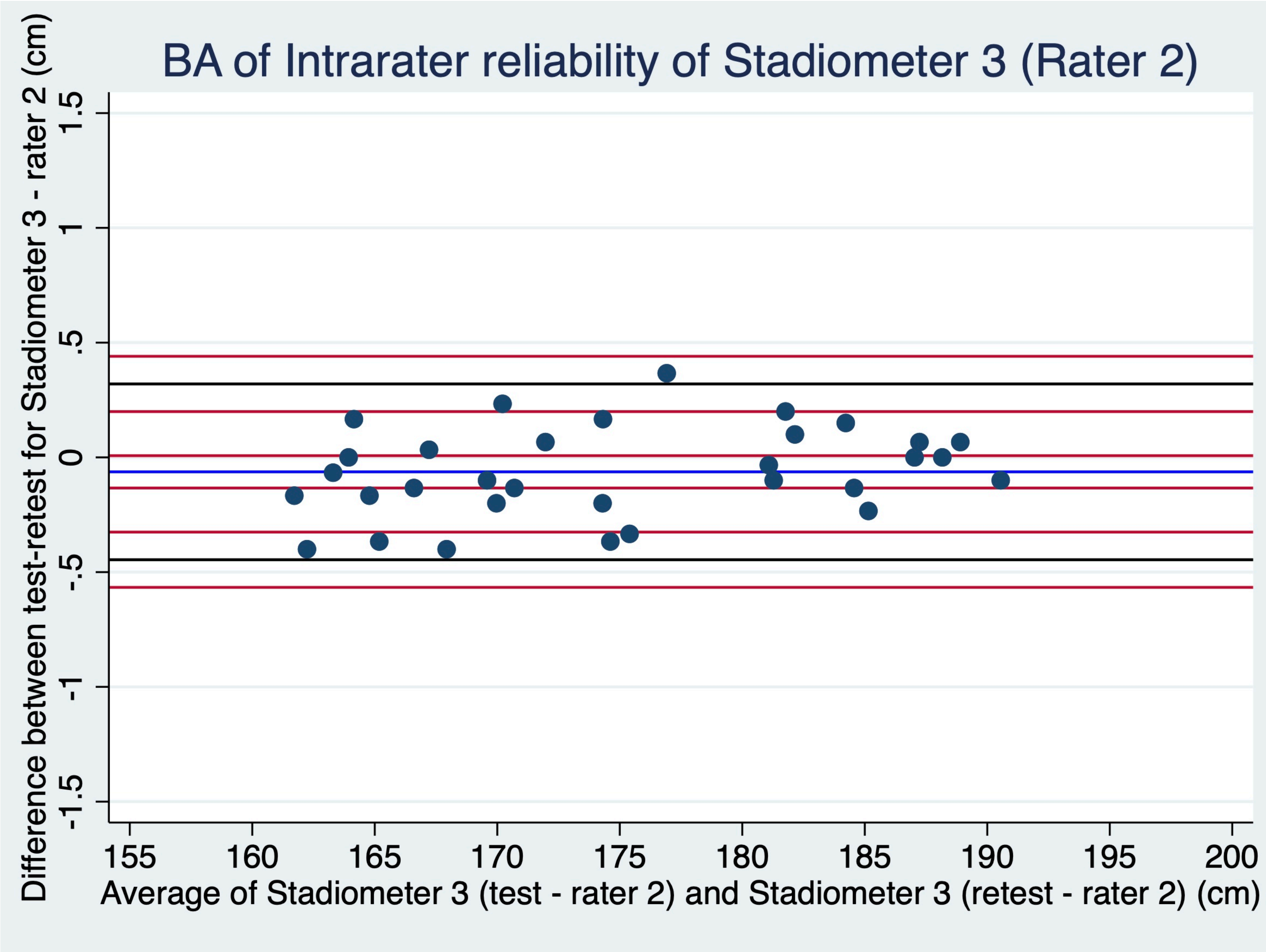
BA of Intrarater reliability of Stadiometer 3 (Rater 2)
Difference between test-retest for Stadiometer 3 - rater 2 (cm)
1.5
1
.5
0
-.5
-1
-1.5
155
160
165
170
175
180
185
190
195
200
Average of Stadiometer 3 (test - rater 2) and Stadiometer 3 (retest - rater 2) (cm)

Interrater reliability

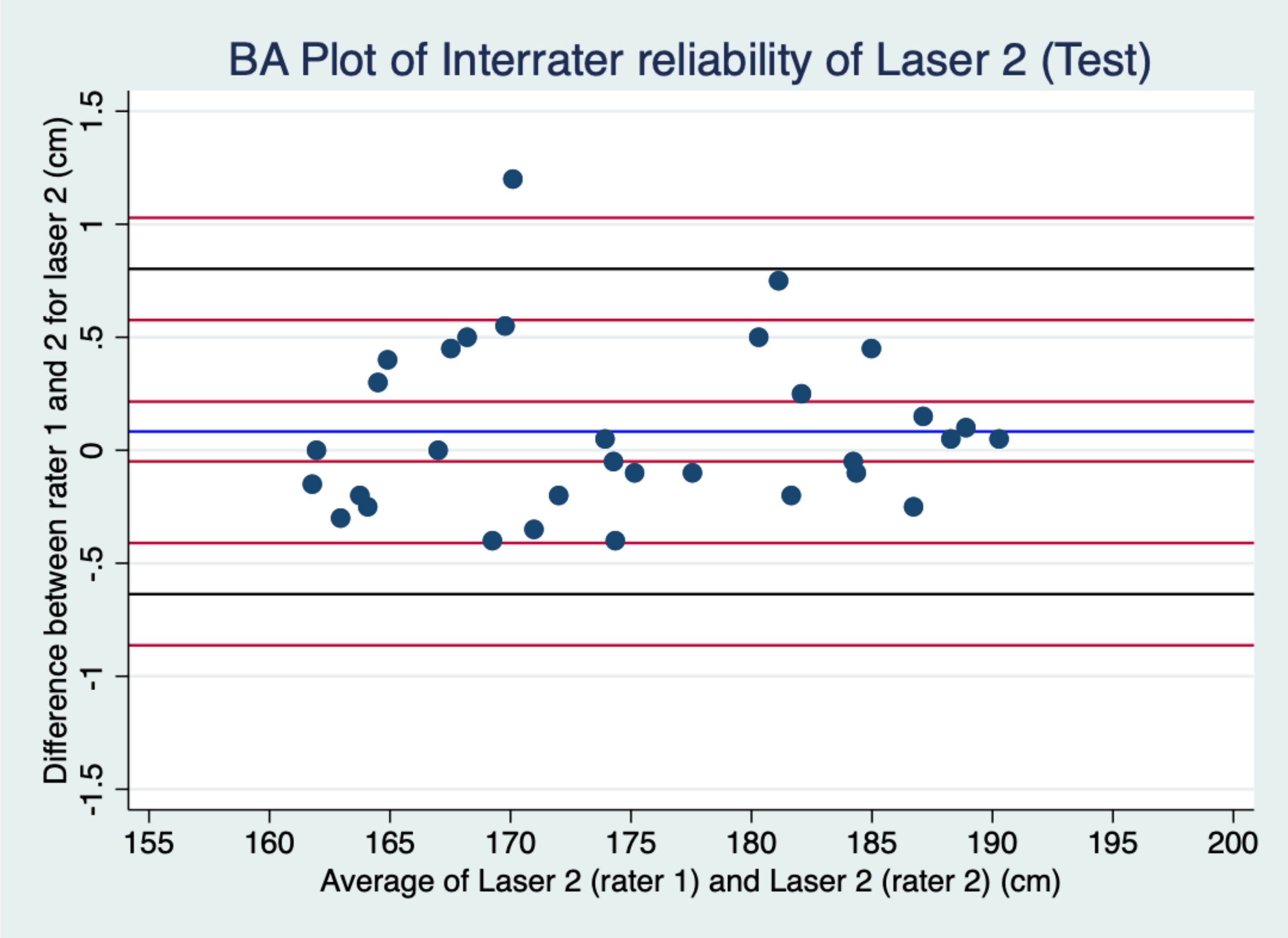

BA Plot of Interrater reliability of Laser 2 (Test)
Difference between rater 1 and 2 for laser 2 (cm)
1.5
1
.5
0
-.5
-1
-1.5
155
160
165
170
175
180
185
190
195
200
Average of Laser 2 (rater 1) and Laser 2 (rater 2) (cm)

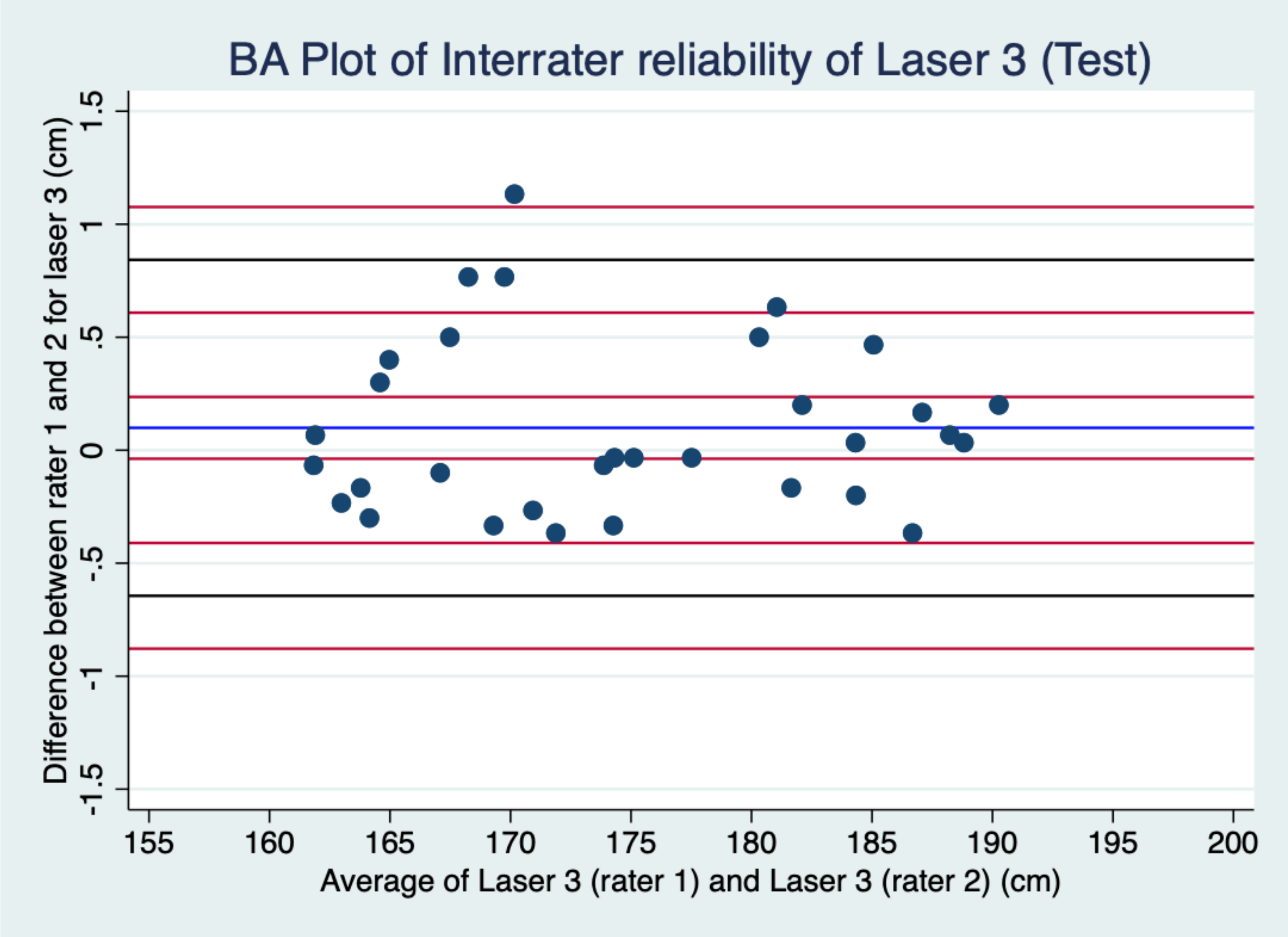
BA Plot of Interrater reliability of Laser 3 (Test)
Difference between rater 1 and 2 for laser 3 (cm)
-1.5
-1
-.5
0
.5
1
1.5
155
160
165
170
175
180
185
190
195
200
Average of Laser 3 (rater 1) and Laser 3 (rater 2) (cm)

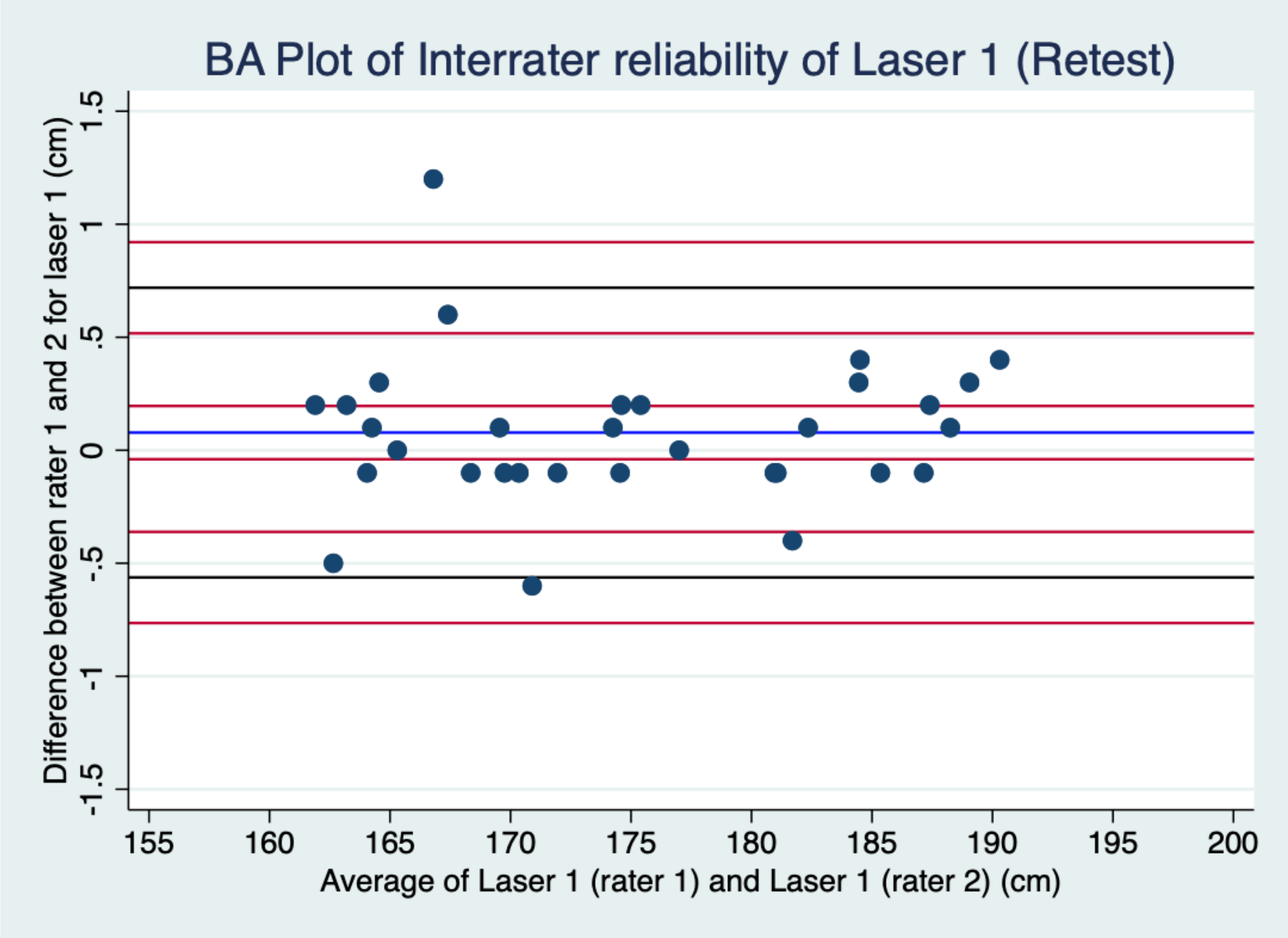
BA Plot of Interrater reliability of Laser 1 (Retest)
Difference between rater 1 and 2 for laser 1 (cm)
-1.5
-1
-.5
0
.5
1
1.5
155
160
165
170
175
180
185
190
195
200
Average of Laser 1 (rater 1) and Laser 1 (rater 2) (cm)

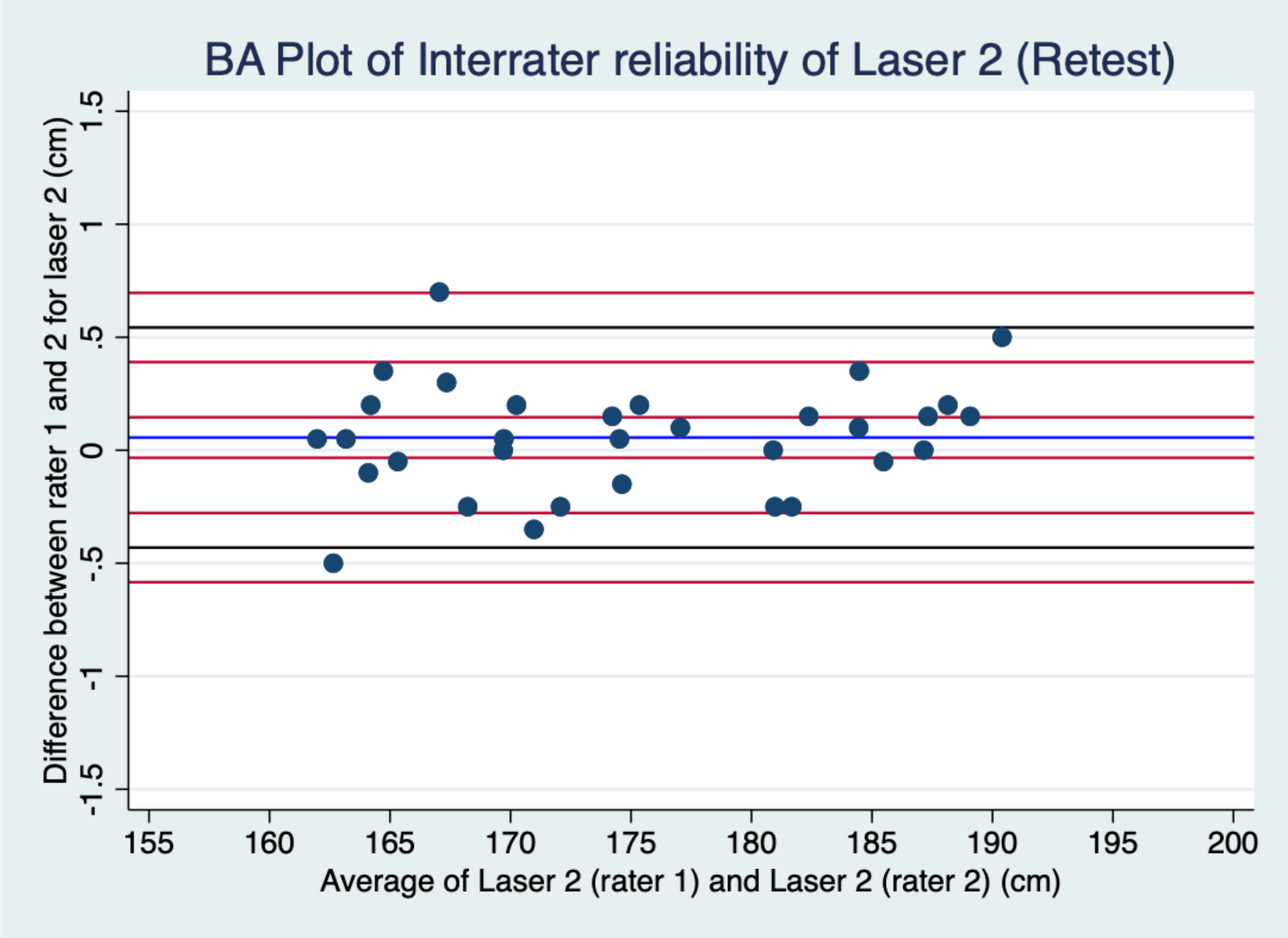

BA Plot of Interrater reliability of Laser 2 (Retest)
Difference between rater 1 and 2 for laser 2 (cm)
1.5
1
.5
0
-.5
-1
-1.5
155
160
165
170
175
180
185
190
195
200
Average of Laser 2 (rater 1) and Laser 2 (rater 2) (cm)

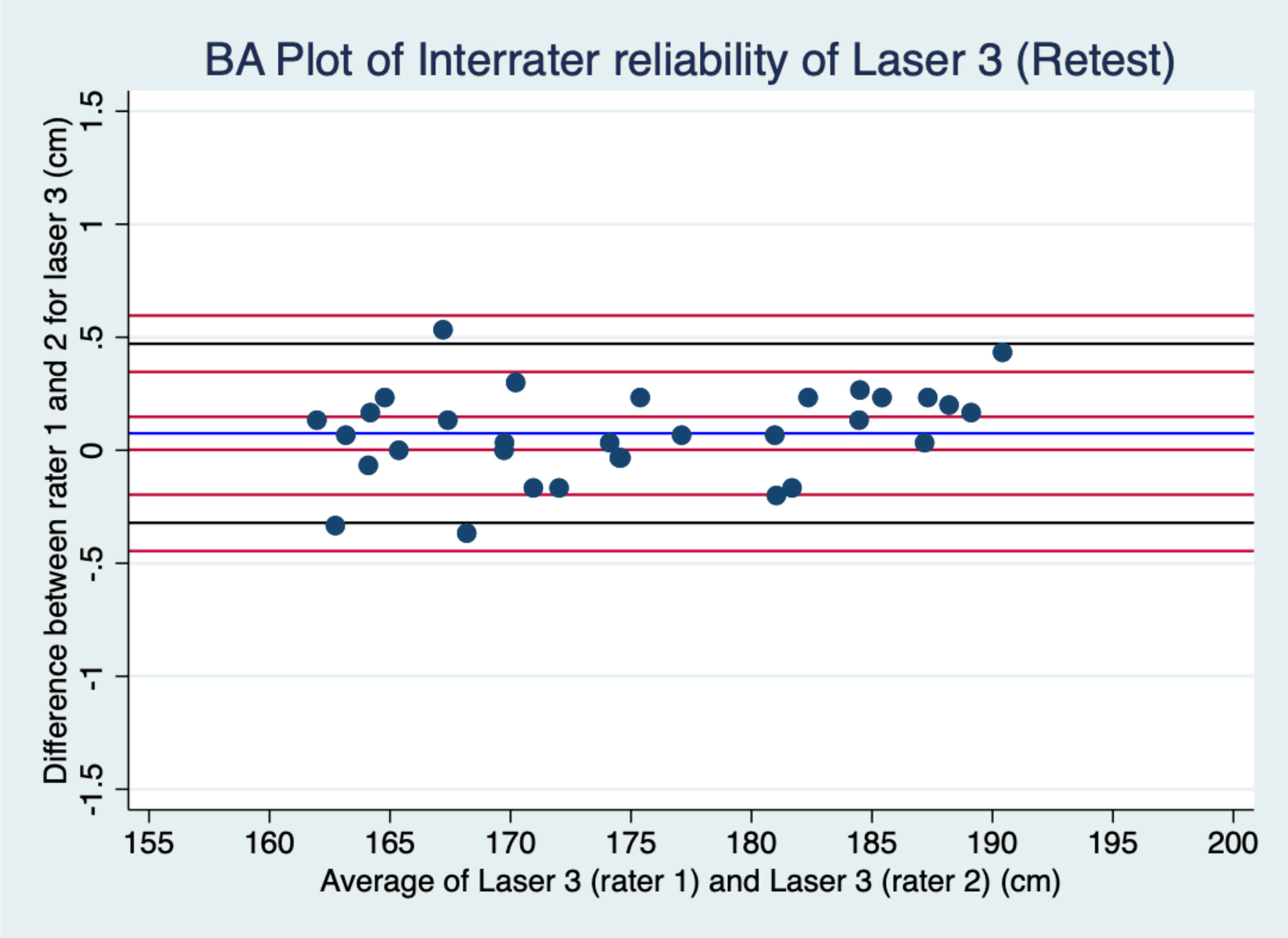
BA Plot of Interrater reliability of Laser 3 (Retest)
Difference between rater 1 and 2 for laser 3 (cm)
-1.5
-1
-.5
0
.5
1
1.5
155
160
165
170
175
180
185
190
195
200
Average of Laser 3 (rater 1) and Laser 3 (rater 2) (cm)

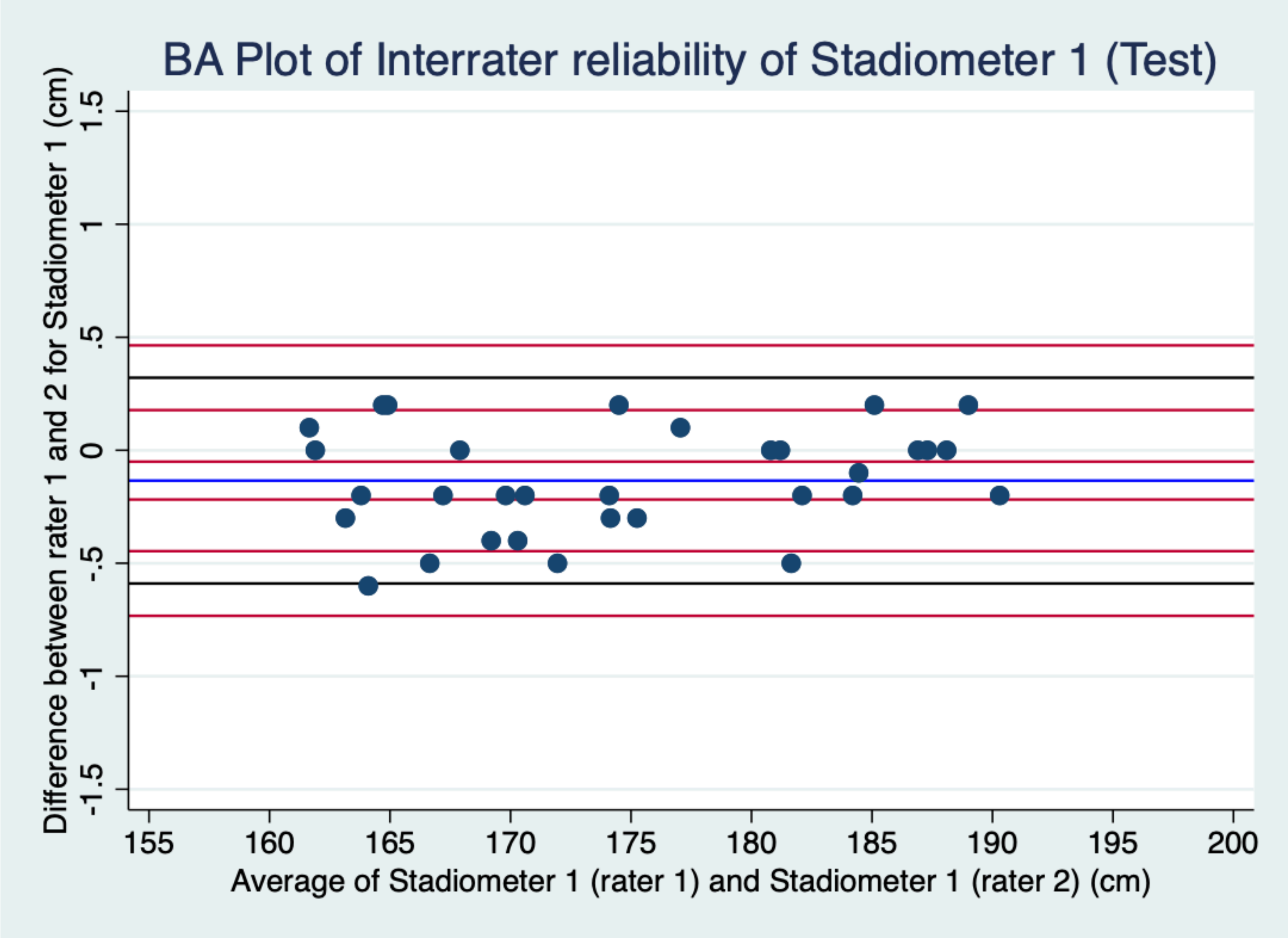

BA Plot of Interrater reliability of Stadiometer 1 (Test)
Difference between rater 1 and 2 for Stadiometer 1 (cm)
1.5
1
.5
0
-.5
-1
-1.5
155
160
165
170
175
180
185
190
195
200
Average of Stadiometer 1 (rater 1) and Stadiometer 1 (rater 2) (cm)

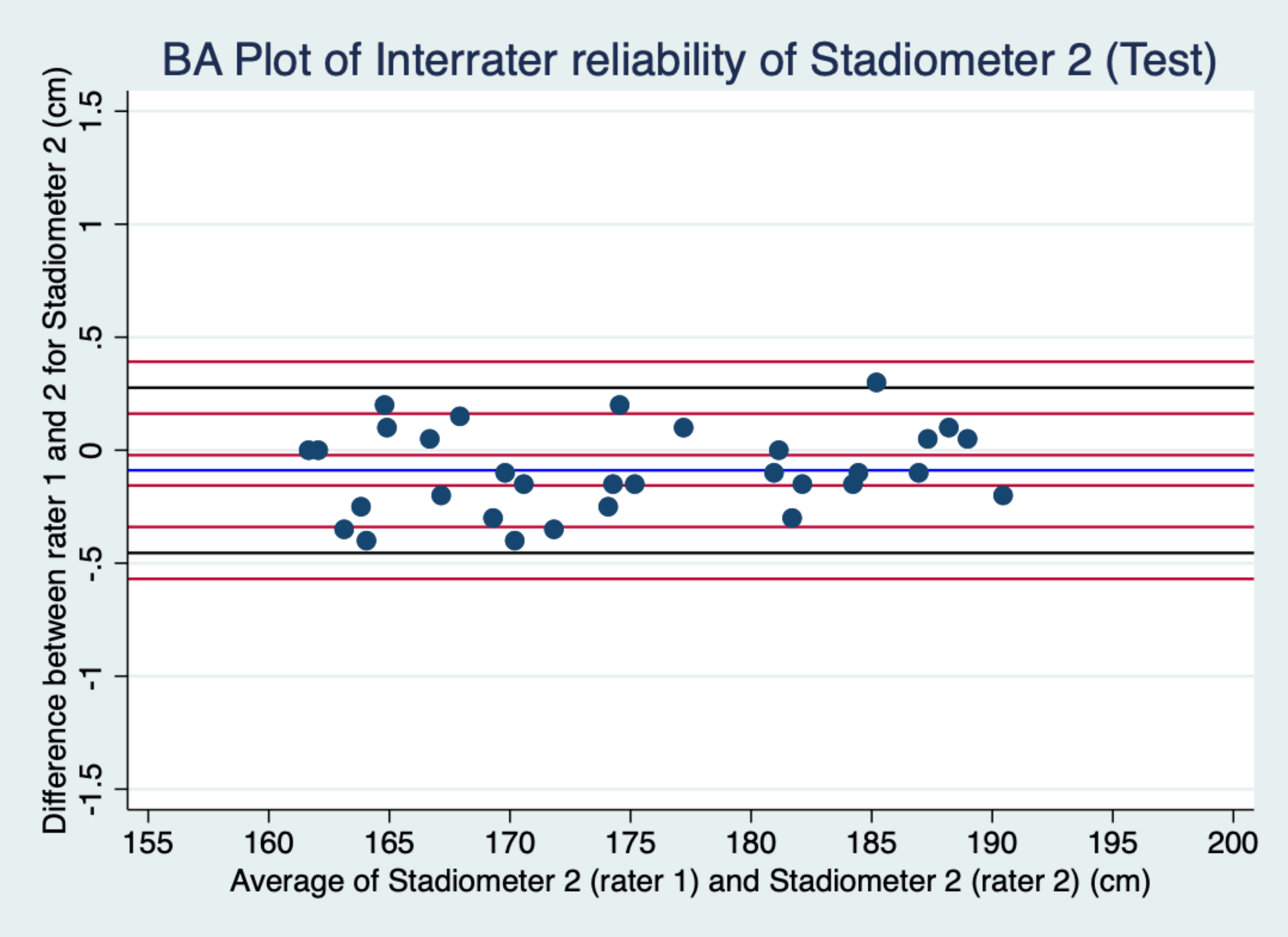
BA Plot of Interrater reliability of Stadiometer 2 (Test)
Difference between rater 1 and 2 for Stadiometer 2 (cm)
1.5
1
.5
0
-.5
-1
-1.5
155
160
165
170
175
180
185
190
195
200
Average of Stadiometer 2 (rater 1) and Stadiometer 2 (rater 2) (cm)

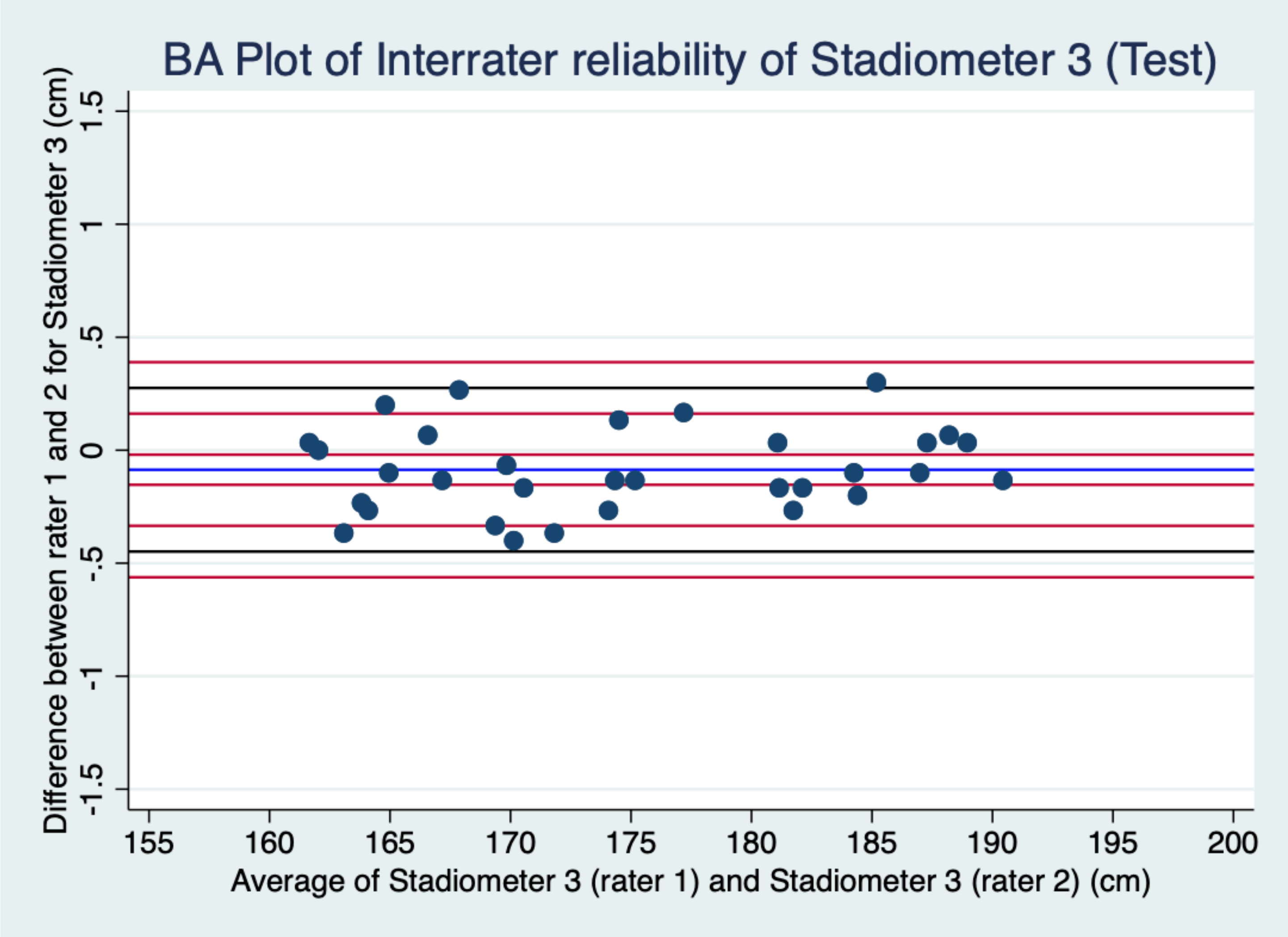
BA Plot of Interrater reliability of Stadiometer 3 (Test)
Difference between rater 1 and 2 for Stadiometer 3 (cm)
-1.5
-1
-.5
0
.5
1
1.5
155
160
165
170
175
180
185
190
195
200
Average of Stadiometer 3 (rater 1) and Stadiometer 3 (rater 2) (cm)

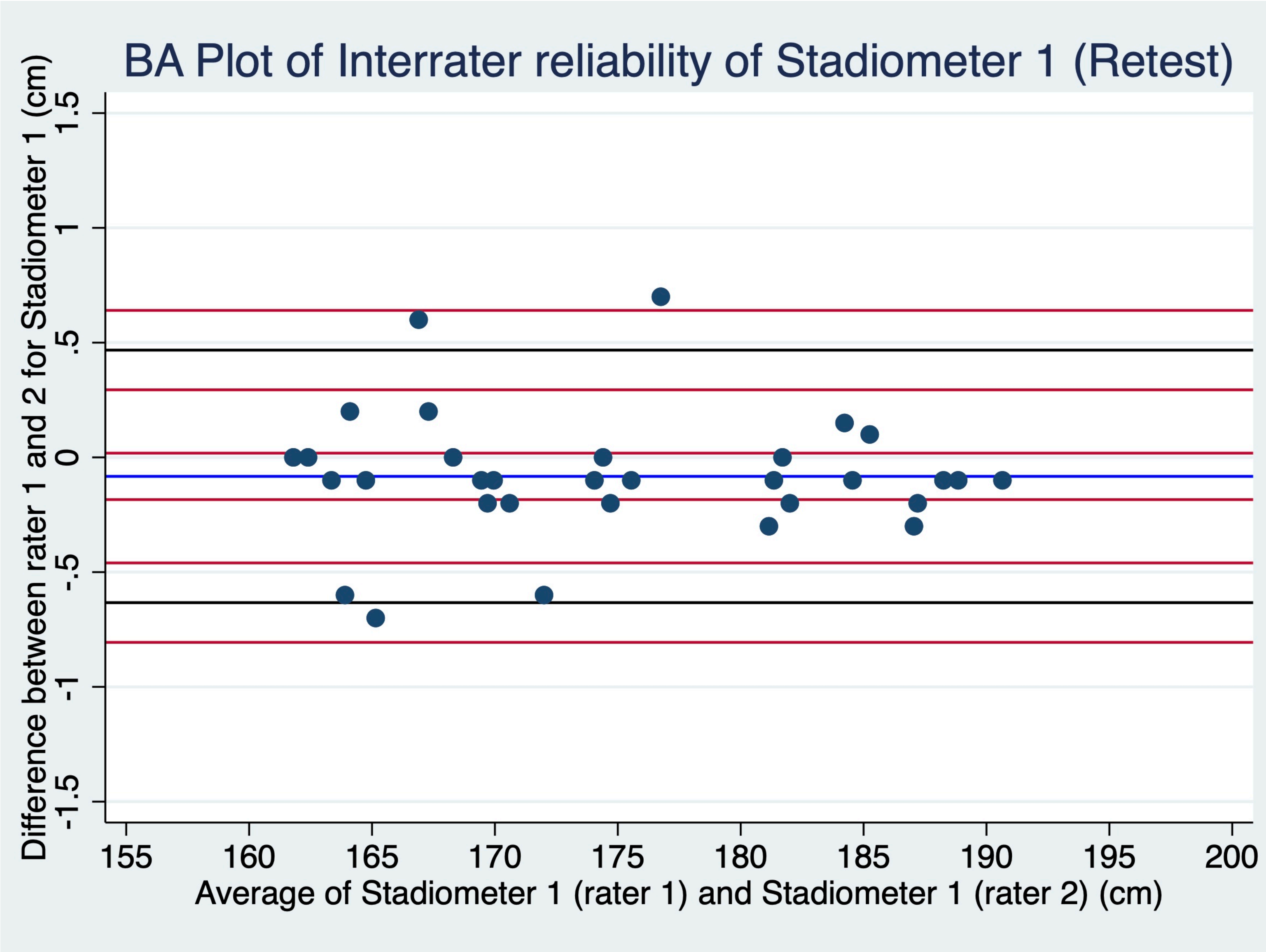
BA Plot of Interrater reliability of Stadiometer 1 (Retest)
Difference between rater 1 and 2 for Stadiometer 1 (cm)
1.5
1
.5
0
-.5
-1
-1.5
155
160
165
170
175
180
185
190
195
200
Average of Stadiometer 1 (rater 1) and Stadiometer 1 (rater 2) (cm)

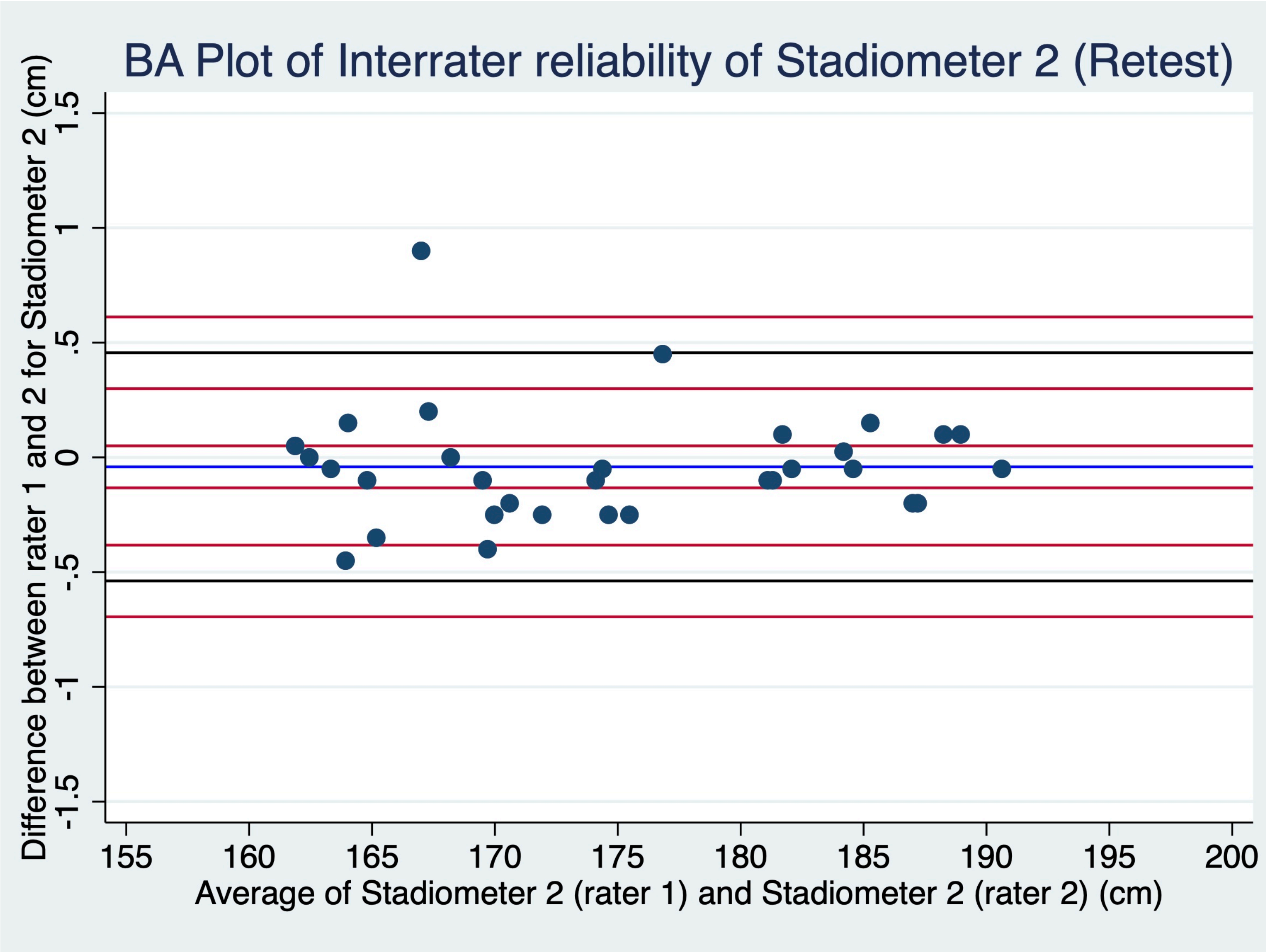
BA Plot of Interrater reliability of Stadiometer 2 (Retest)
Difference between rater 1 and 2 for Stadiometer 2 (cm)
1.5
1
.5
0
-.5
-1
-1.5
155
160
165
170
175
180
185
190
195
200
Average of Stadiometer 2 (rater 1) and Stadiometer 2 (rater 2) (cm)

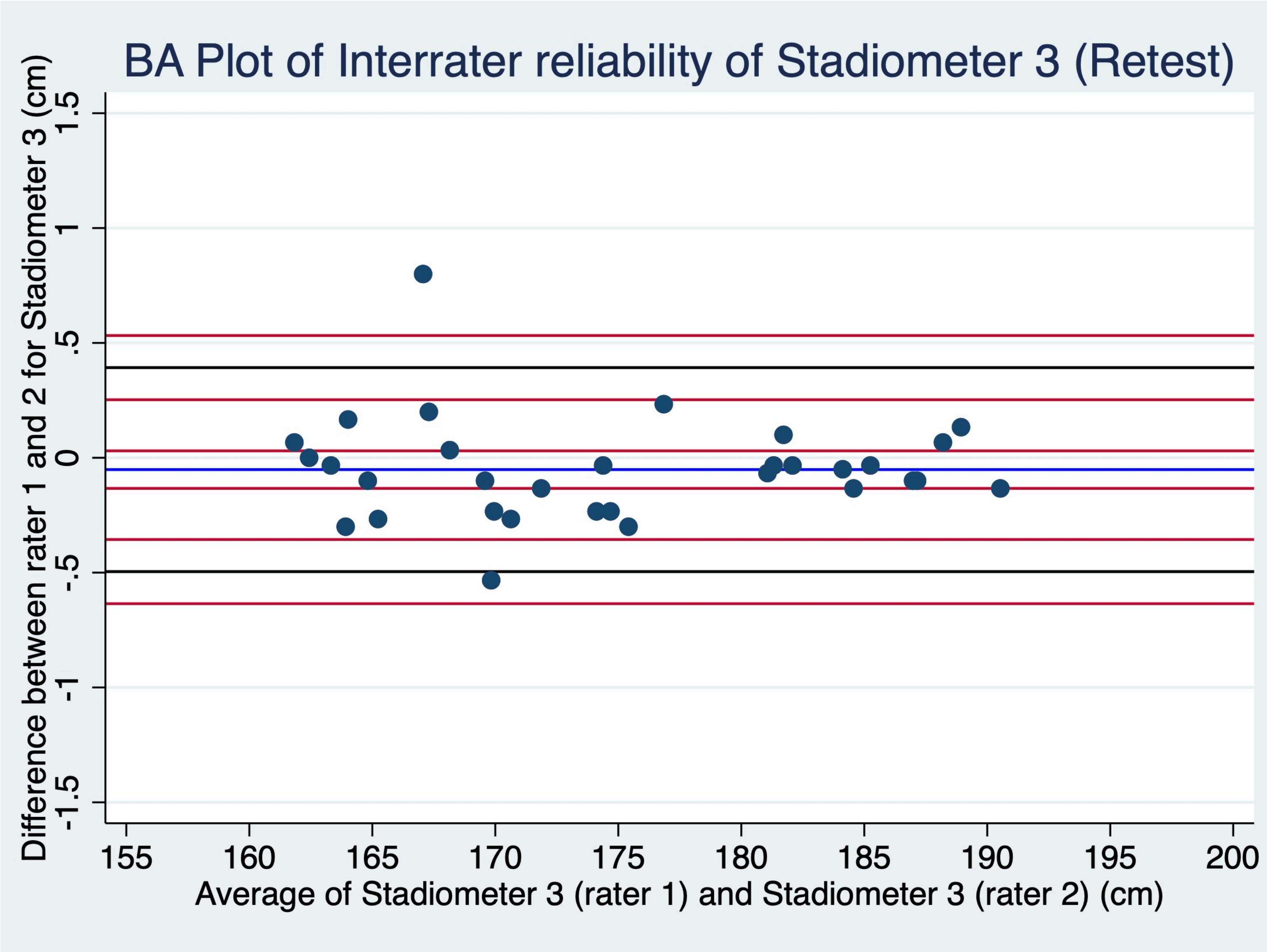
BA Plot of Interrater reliability of Stadiometer 3 (Retest)
Difference between rater 1 and 2 for Stadiometer 3 (cm)
1.5
1
.5
0
-.5
-1
-1.5
155
160
165
170
175
180
185
190
195
200
Average of Stadiometer 3 (rater 1) and Stadiometer 3 (rater 2) (cm)

Method comparison

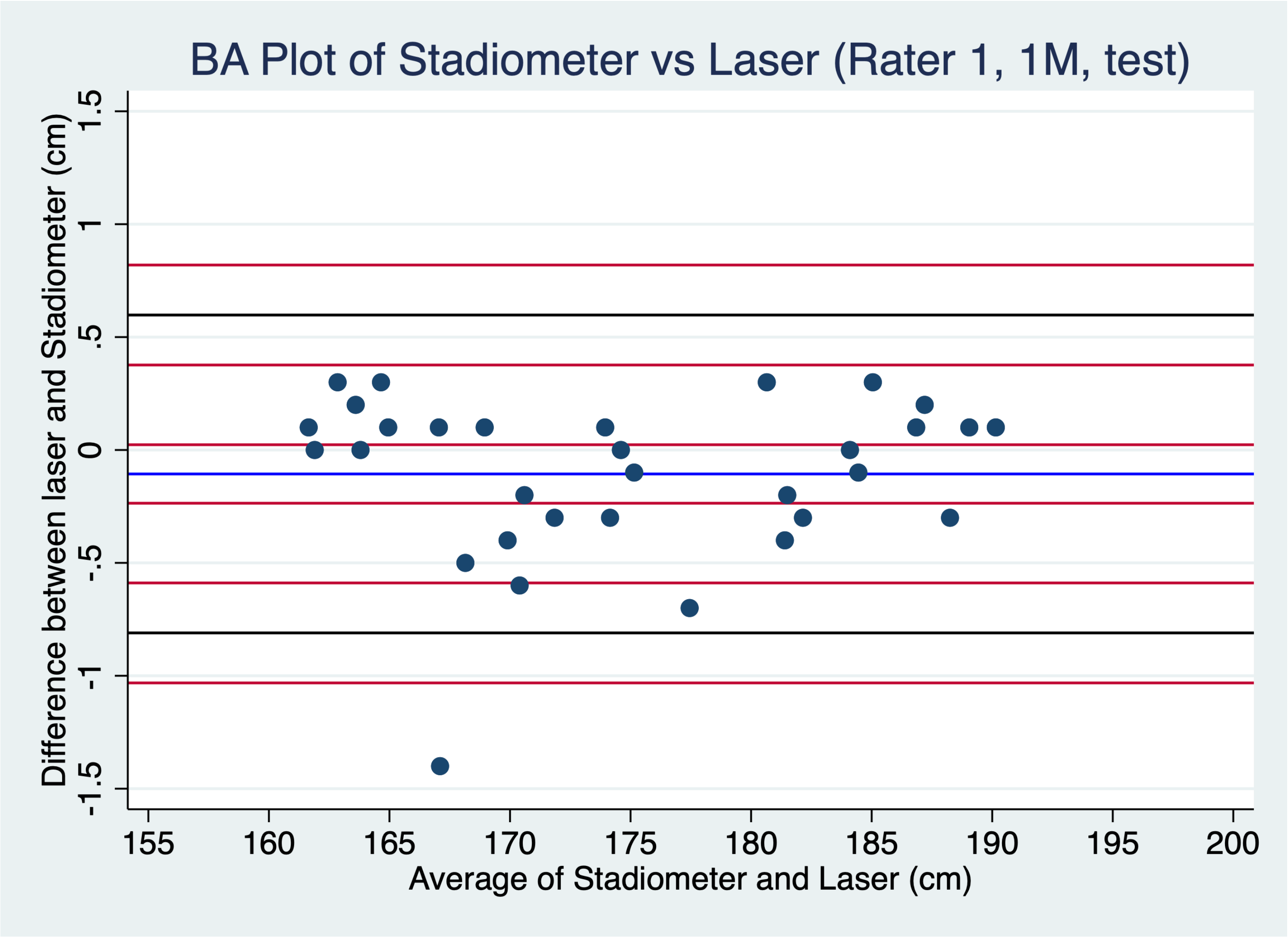

BA Plot of Stadiometer vs Laser (Rater 1, 1M, test)
Difference between laser and Stadiometer (cm)
-1.5
-1
-.5
0
.5
1
1.5
155
160
165
170
175
180
185
190
195
200
Average of Stadiometer and Laser (cm)

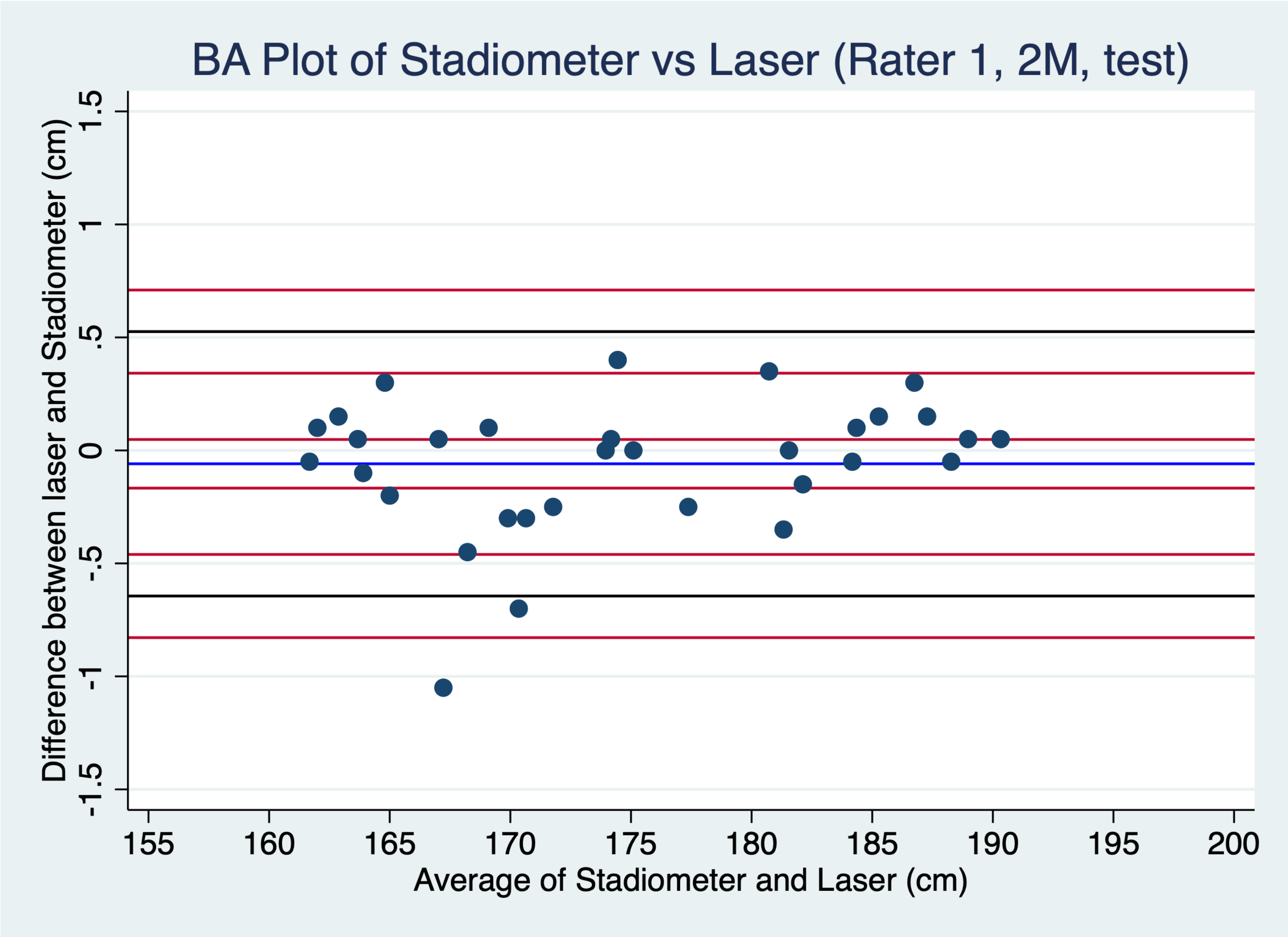

BA Plot of Stadiometer vs Laser (Rater 1, 2M, test)
Difference between laser and Stadiometer (cm)
-1.5
-1
-.5
0
.5
1
1.5
155
160
165
170
175
180
185
190
195
200
Average of Stadiometer and Laser (cm)

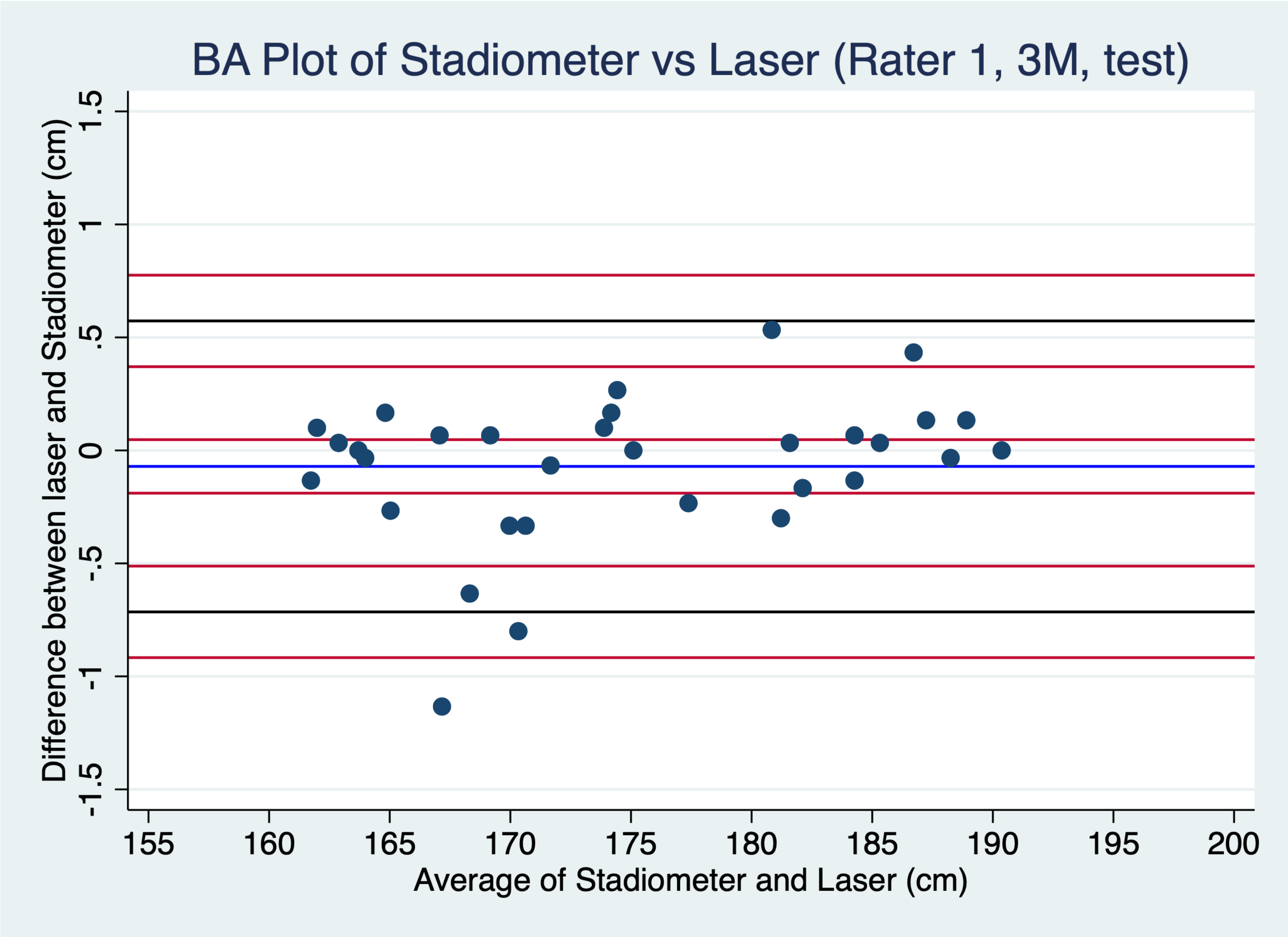
BA Plot of Stadiometer vs Laser (Rater 1, 3M, test)
Difference between laser and Stadiometer (cm)
-1.5
-1
-.5
0
.5
1
1.5
155
160
165
170
175
180
185
190
195
200
Average of Stadiometer and Laser (cm)

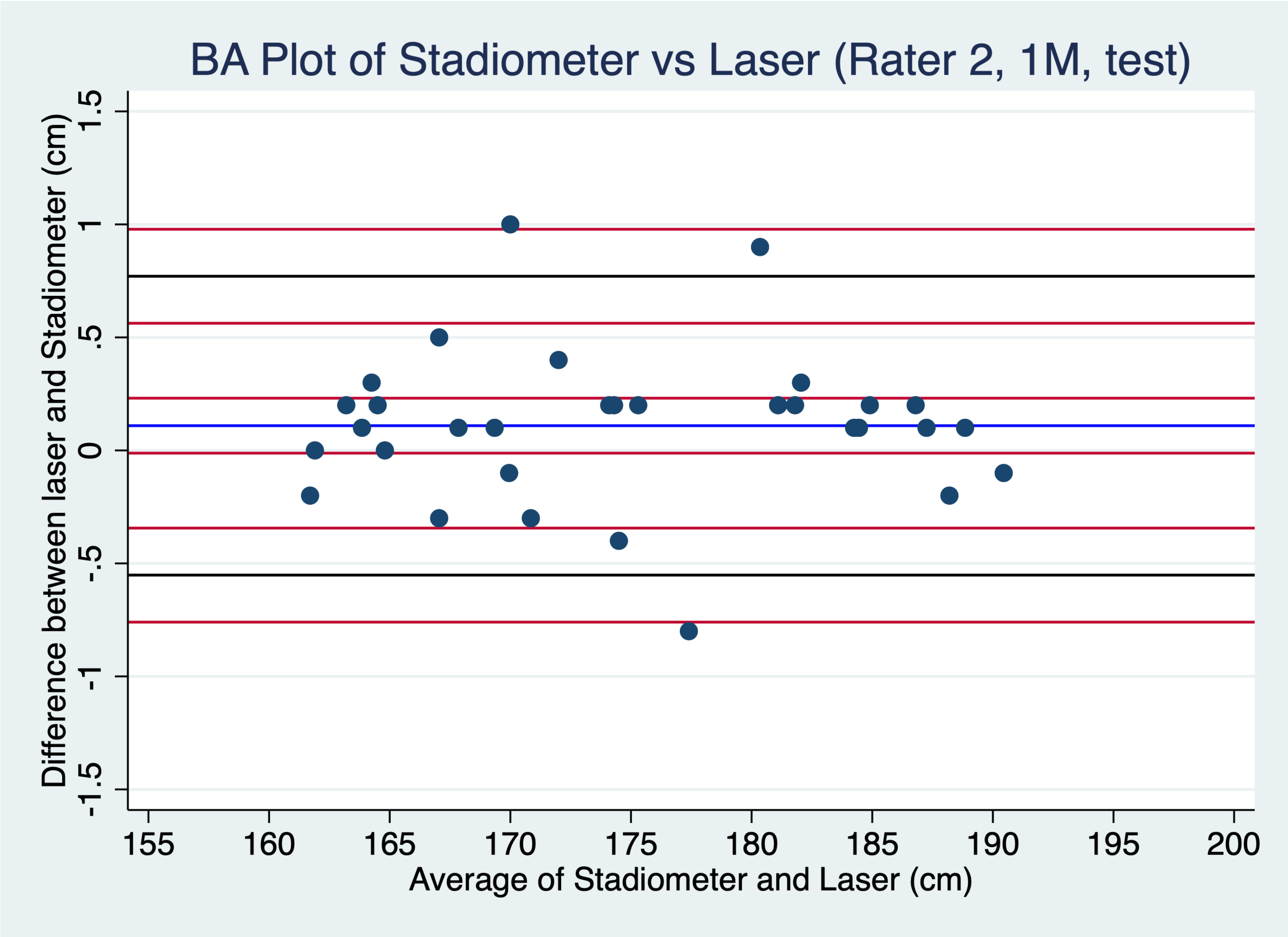
BA Plot of Stadiometer vs Laser (Rater 2, 1M, test)
Difference between laser and Stadiometer (cm)
-1.5
-1
-.5
0
.5
1
1.5
155
160
165
170
175
180
185
190
195
200
Average of Stadiometer and Laser (cm)

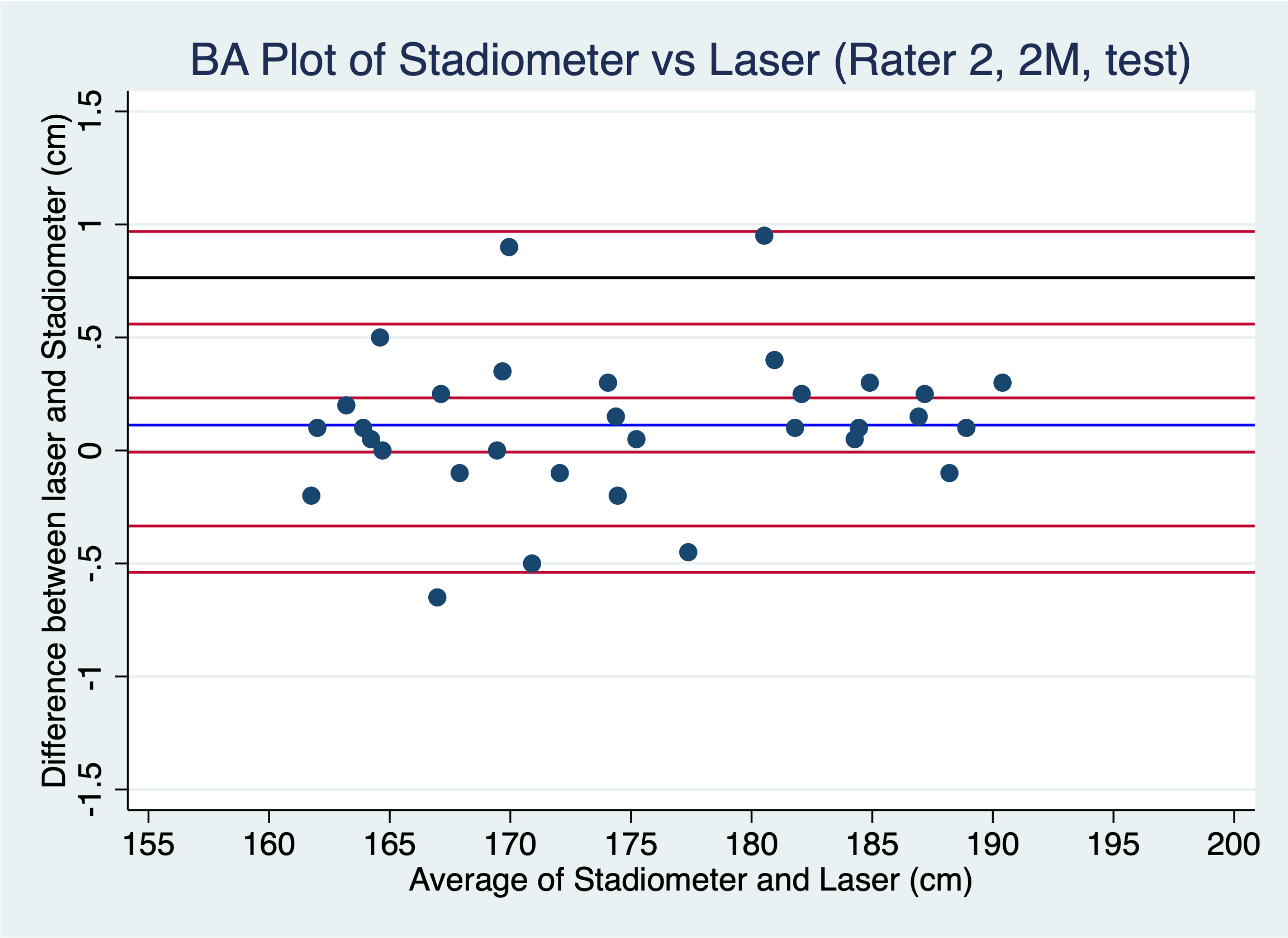

BA Plot of Stadiometer vs Laser (Rater 2, 2M, test)
Difference between laser and Stadiometer (cm)
-1.5
-1
-.5
0
.5
1
1.5
155
160
165
170
175
180
185
190
195
200
Average of Stadiometer and Laser (cm)

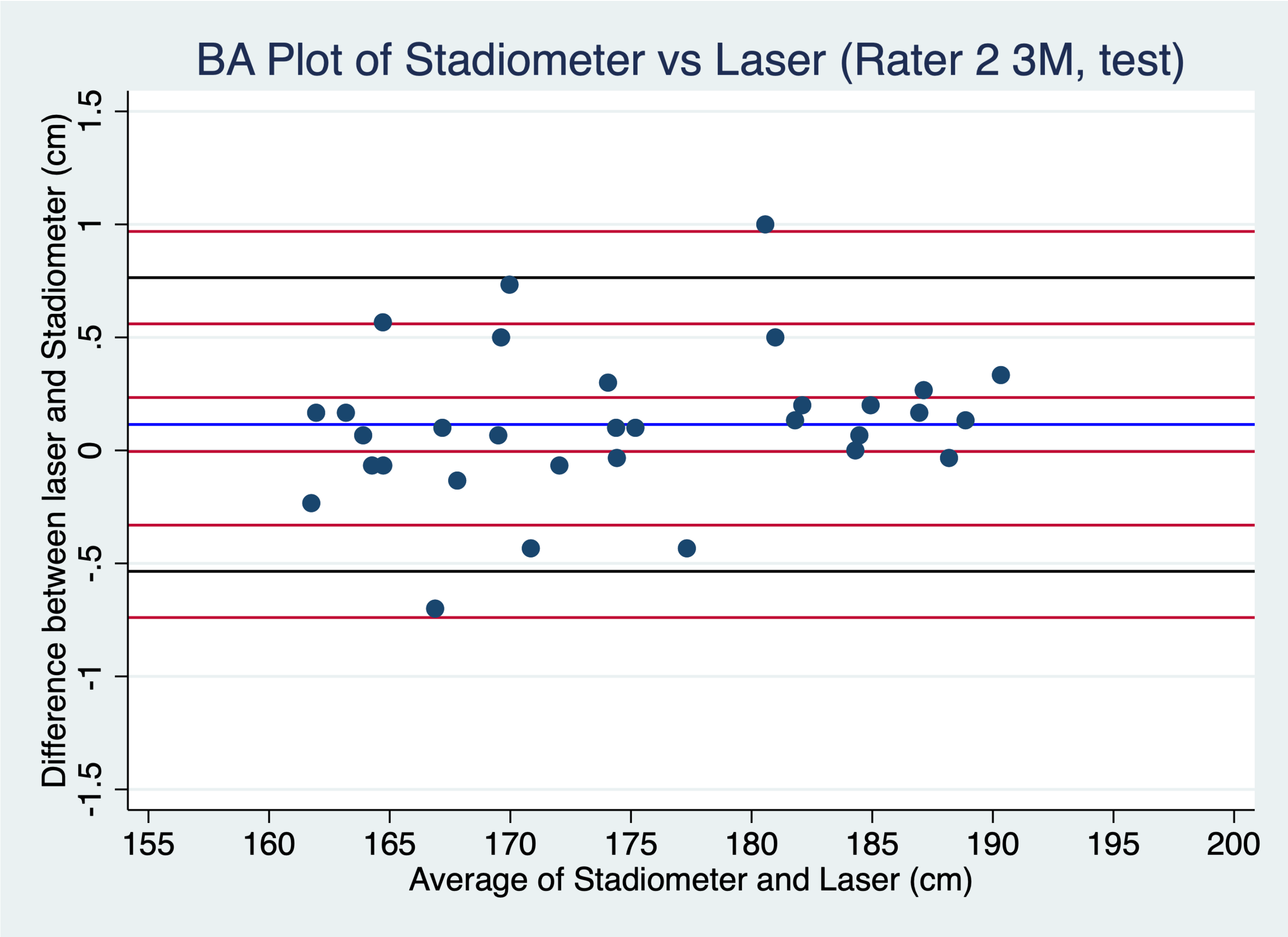

BA Plot of Stadiometer vs Laser (Rater 2 3M, test)
Difference between laser and Stadiometer (cm)
-1.5
-1
-.5
0
.5
1
1.5
155
160
165
170
175
180
185
190
195
200
Average of Stadiometer and Laser (cm)

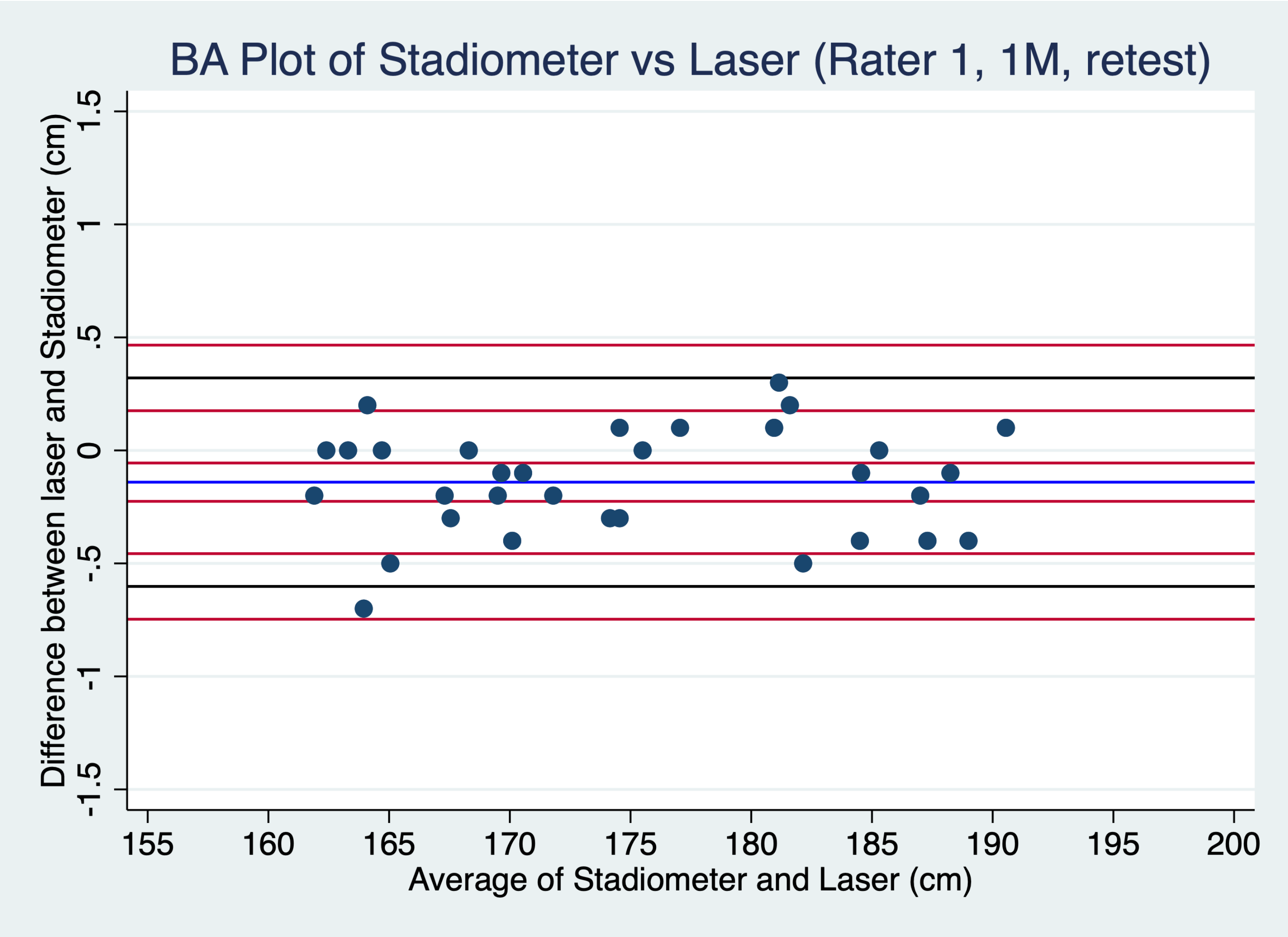
BA Plot of Stadiometer vs Laser (Rater 1, 1M, retest)
Difference between laser and Stadiometer (cm)
1.5
1
.5
0
-.5
-1
-1.5
155
160
165
170
175
180
185
190
195
200
Average of Stadiometer and Laser (cm)

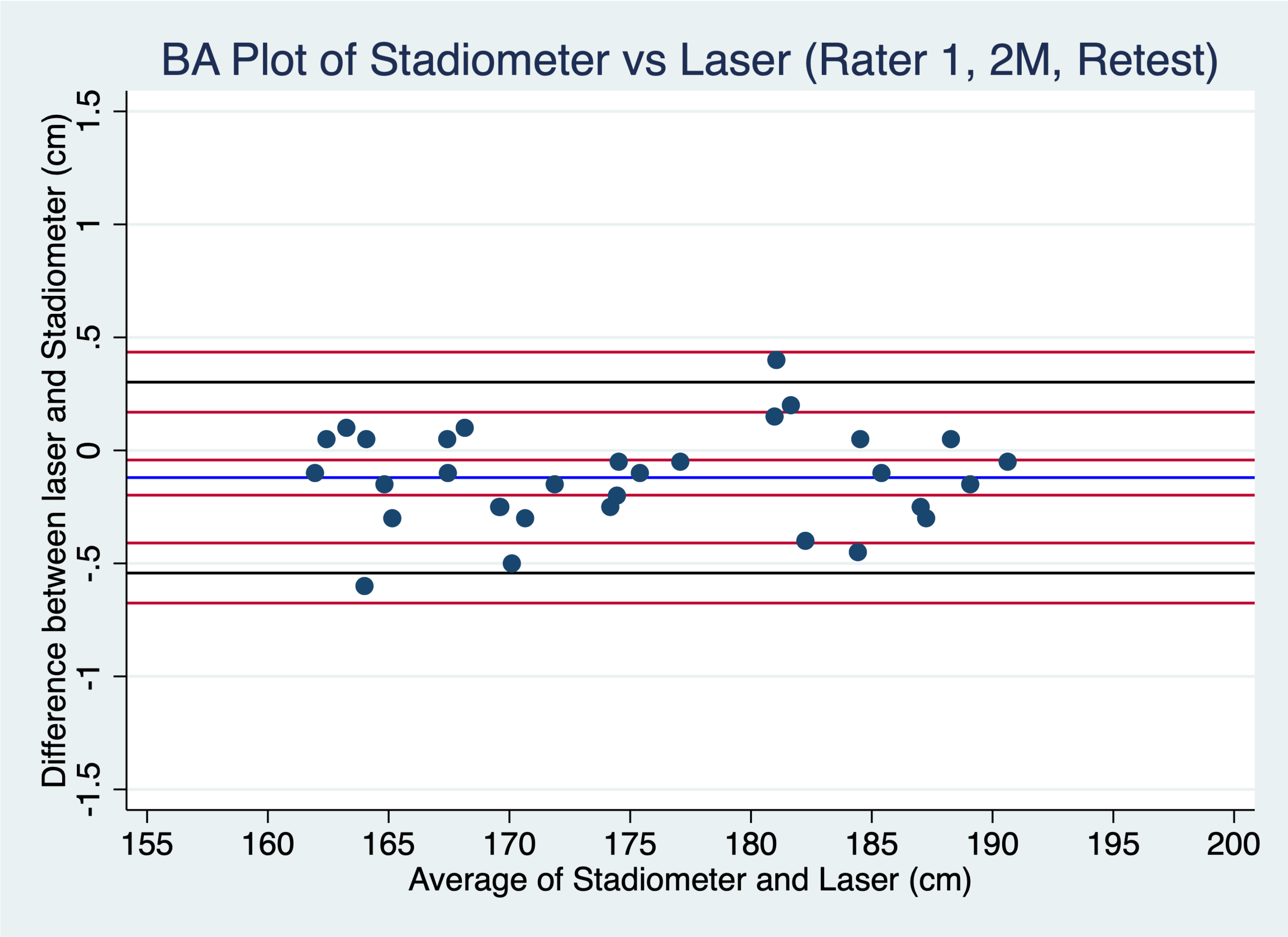
BA Plot of Stadiometer vs Laser (Rater 1, 2M, Retest)
Difference between laser and Stadiometer (cm)
-1.5
-1
-.5
0
.5
1
1.5
155
160
165
170
175
180
185
190
195
200
Average of Stadiometer and Laser (cm)

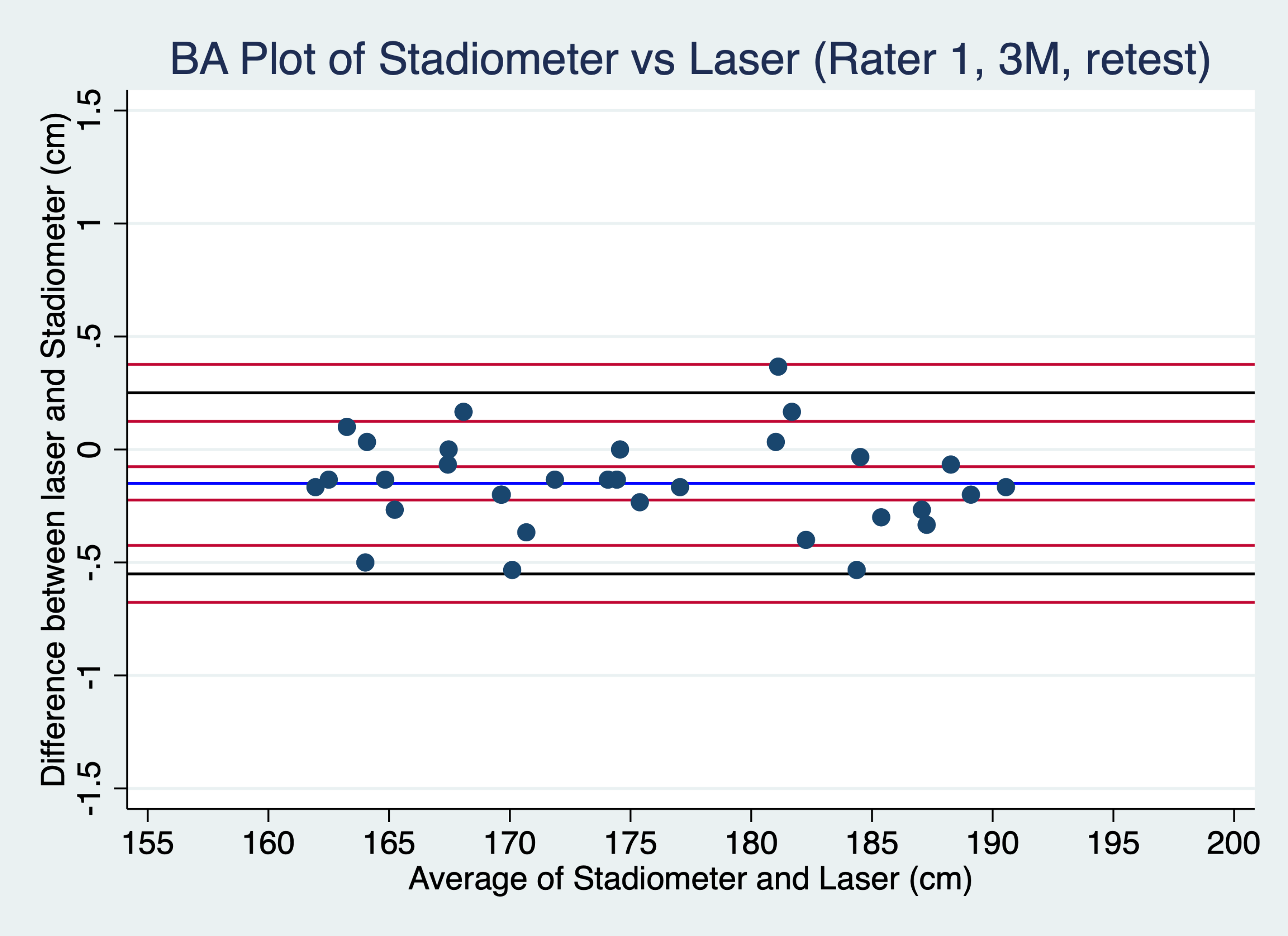

BA Plot of Stadiometer vs Laser (Rater 1, 3M, retest)
Difference between laser and Stadiometer (cm)
1.5
1
.5
0
-.5
-1
-1.5
155
160
165
170
175
180
185
190
195
200
Average of Stadiometer and Laser (cm)

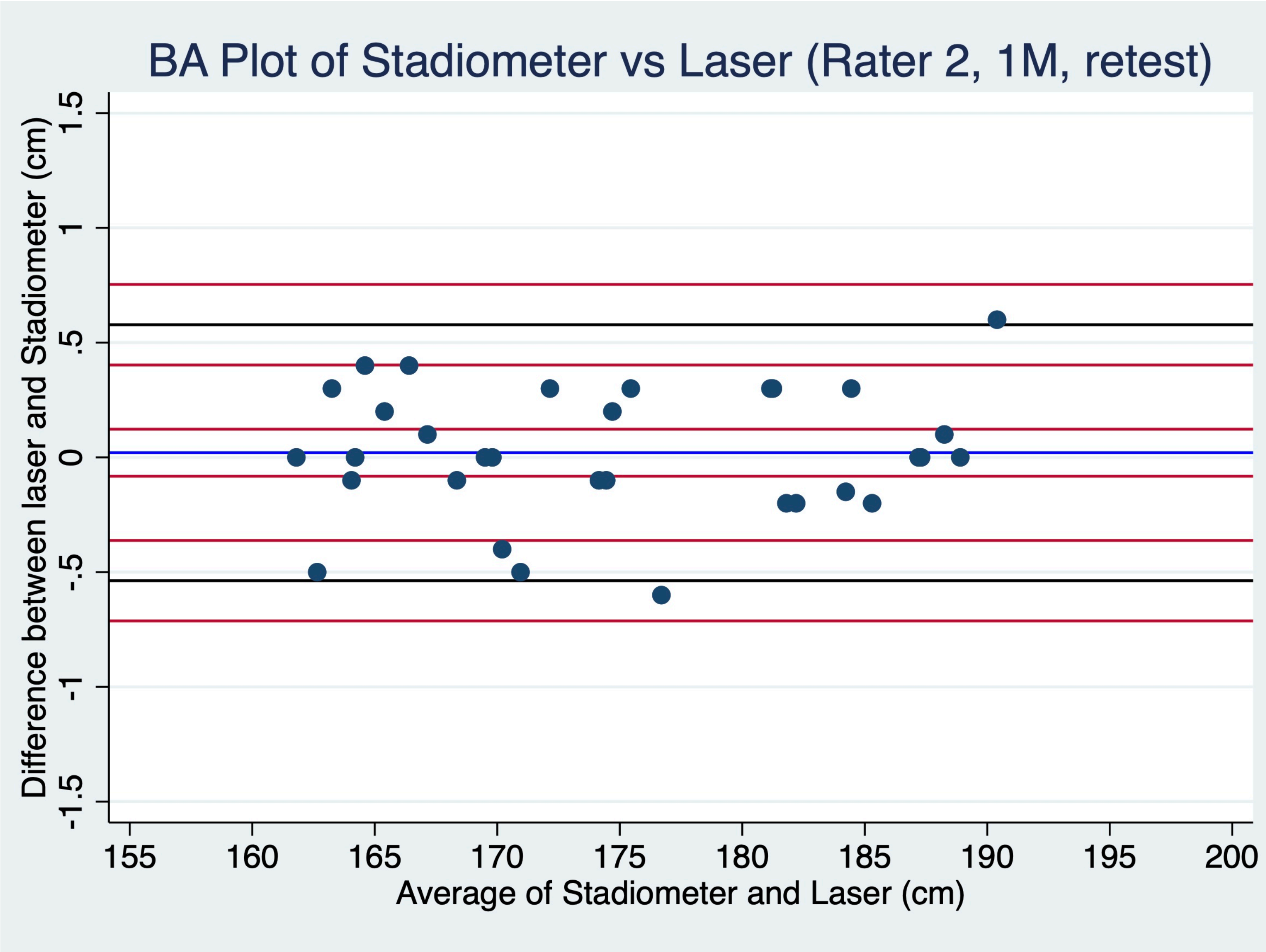
BA Plot of Stadiometer vs Laser (Rater 2, 1M, retest)
Difference between laser and Stadiometer (cm)
1.5
1
.5
0
-.5
-1
-1.5
155
160
165
170
175
180
185
190
195
200
Average of Stadiometer and Laser (cm)

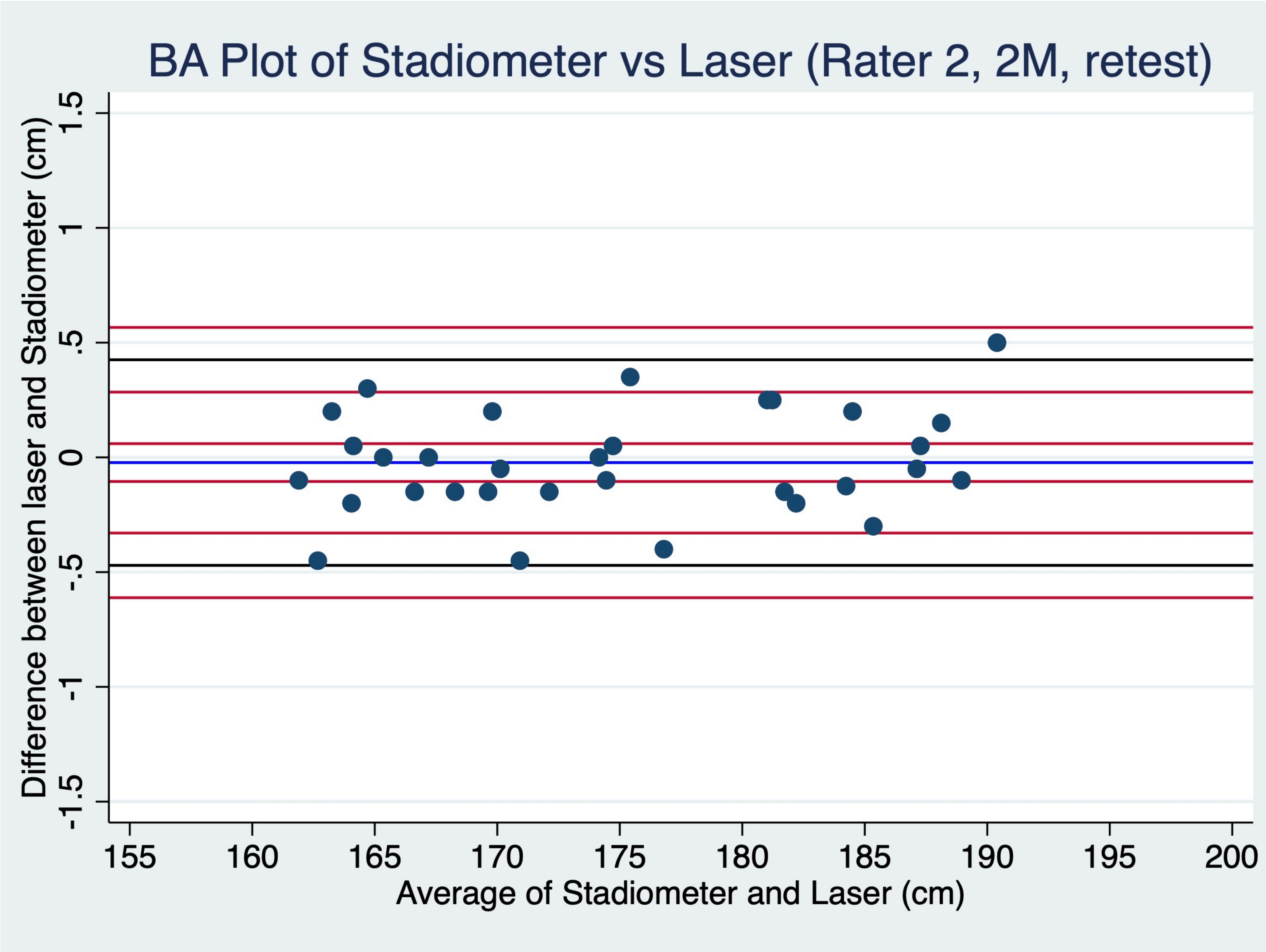

BA Plot of Stadiometer vs Laser (Rater 2, 2M, retest)
Difference between laser and Stadiometer (cm)
-1.5
-1
-.5
0
.5
1
1.5
155
160
165
170
175
180
185
190
195
200
Average of Stadiometer and Laser (cm)

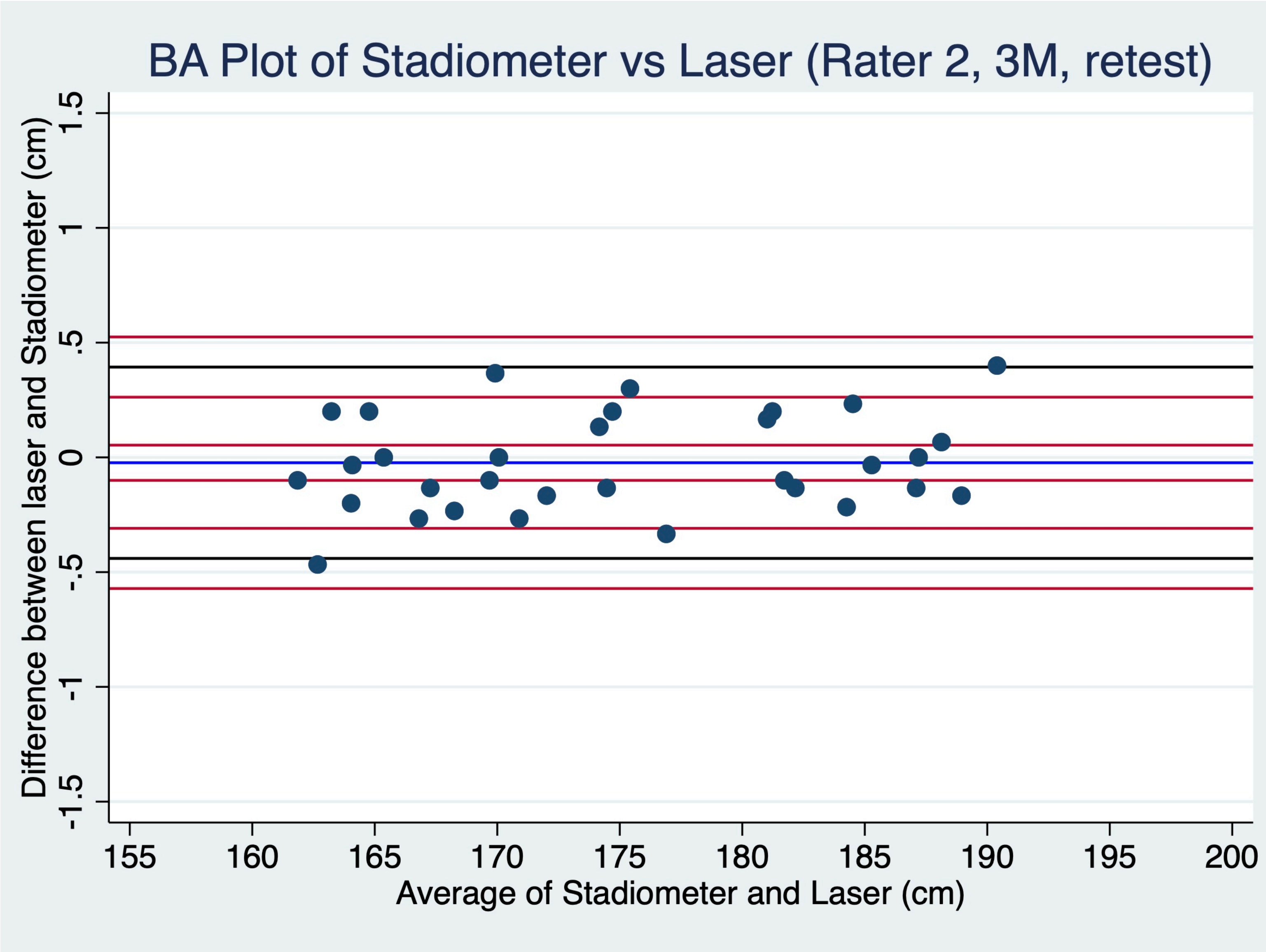
BA Plot of Stadiometer vs Laser (Rater 2, 3M, retest)
Difference between laser and Stadiometer (cm)
1.5
1
.5
0
-.5
-1
-1.5
155
160
165
170
175
180
185
190
195
200
Average of Stadiometer and Laser (cm)
